# Supplementary material for: A natural transdifferentiation event involving mitosis is empowered by integrating signaling inputs with conserved plasticity factors
Source: Cell Rep. 2022 Sep 20;40(12):111365. doi: 10.1016/j.celrep.2022.111365 (PMC9513805; doi:10.1016/j.celrep.2022.111365)
Supplement: Document S1. Figures S1–S13 and Tables S1–S3 [file mmc1.pdf]

Cell Reports, Volume *40*

Supplemental information

A natural transdifferentiation event involving mitosis is empowered by integrating signaling inputs with conserved plasticity factors

Claudia Riva, Martina Hajduskova, Christelle Gally, Shashi Kumar Suman, Arnaud Ahier, and Sophie Jarriault

Fig. S1.

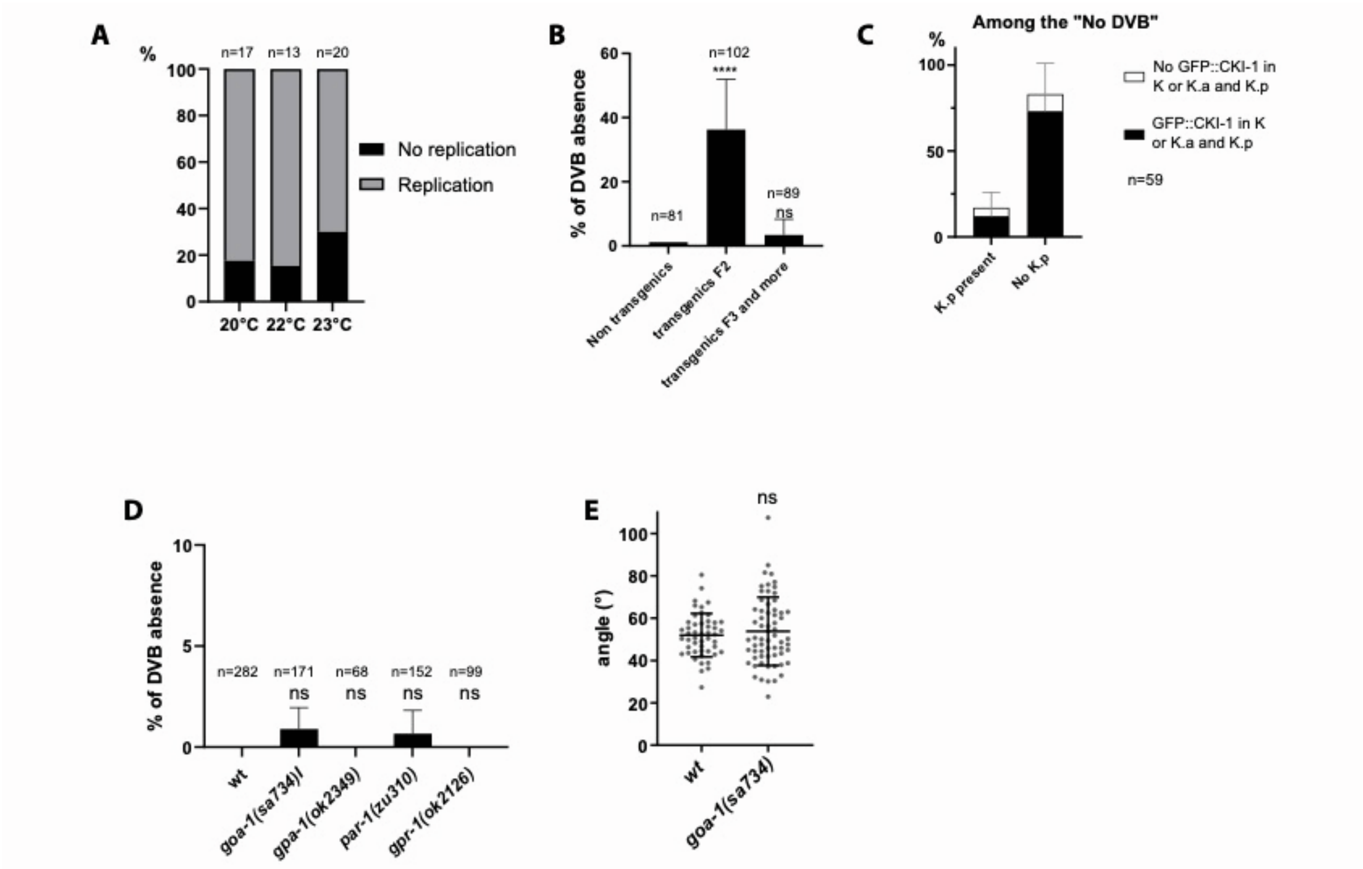

Figure S1. While DNA replication in K is not sufficient for DVB formation and division orientation mutants do not impact K-to-DVB, K division is required for K-to-DVB. Related to Figure 2.

(A) Histogram summarizing the percentage of animals in which K DNA underwent replication in the *lin-5(ev571ts)* mutant at different restrictive temperatures.

(B) Histogram showing the percentage of animals with a "NO DVB" defect, and without (Non transgenics) or with (transgenics) overexpression of *gfp::cki-1* in the rectal cells. Note that for the transgenics, the F2 generation of the transgenic lines was first scored and displayed more penetrant defects than the following generations, due to transgene silencing.

(C) Histogram showing the percentage animals displaying an absence (No K.p) or presence (K.p present) of the K.p cell among the *gfp::cki-1* overexpressing animals without DVB. Note that for each category, the GFP expression was assessed in K, K.a and K.p, when present. Solid bar: GFP expression was observed; white bar, No GFP expression. Error bars, SD of the GFP-expressing animals.

(D) Histogram showing the percentage of worms without DVB in mutants for the Gα and *gpr-1/LGN* genes involved in spindle orientation in *C. elegans* zygote and for the Par gene *par-1*. The low penetrance of DVB absence is due to an impairment in K cytokinesis.

(E) Dot plot representing K division angle in the *goa-1(sa734)* mutant. n=64.

For all the histograms, ns, not significant; n, total animal scored.

Fig. S2.

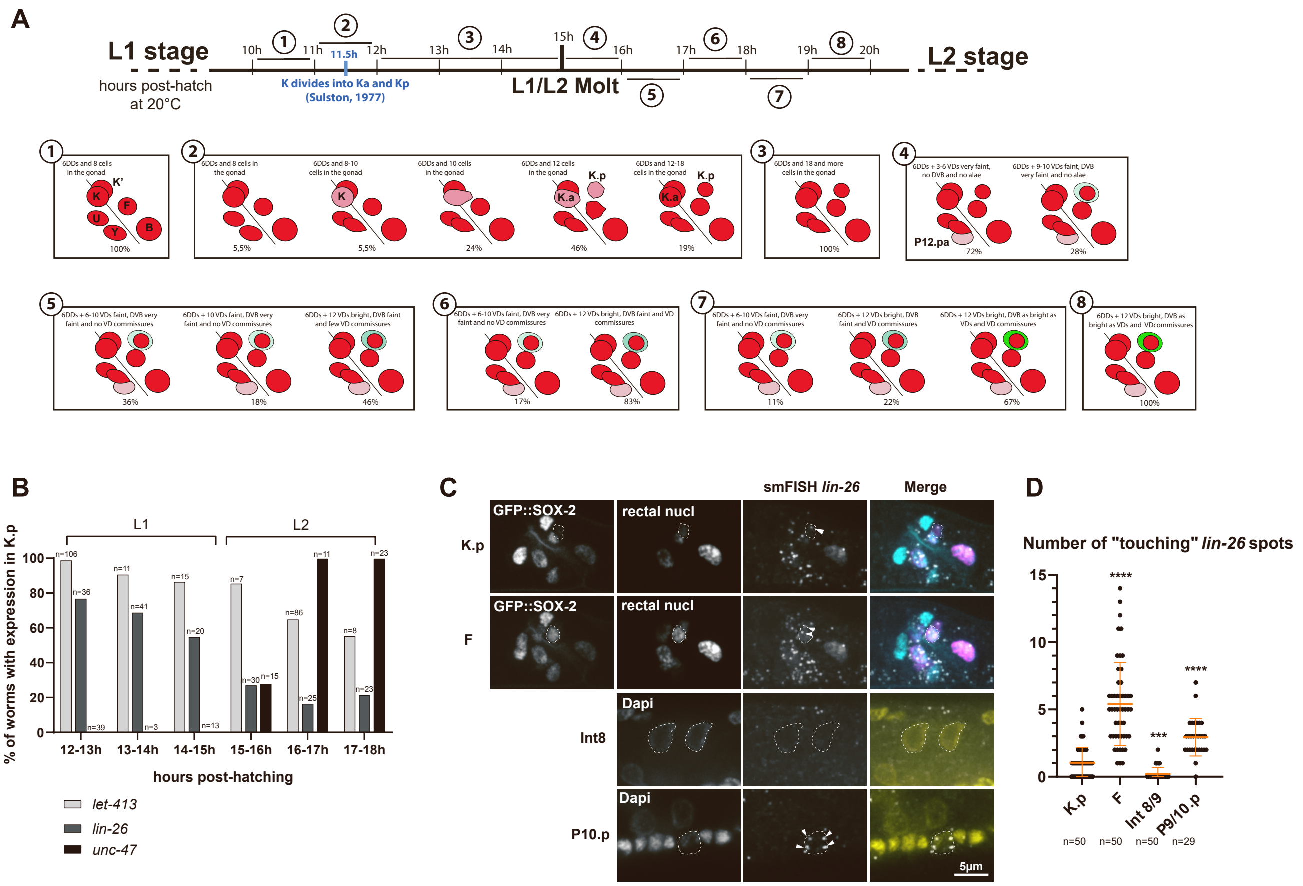

Figure S2. K divides into K.a and K.p in late L1 stage and K.p still shows epithelial features after division. Related to Figure 2.

(A) Time course of the cellular events during K division and DVB formation along the developmental timeline. The *col-34p::mCherry* positive rectal cells and the rectal slit (line) are represented. Each box corresponds to a point of one hour where different characteristic landmarks have been observed in addition to K division: number of GABAergic neurons, VD commissures, presence of alae, number of cells in the gonad, *col-34p::mCherry* and *unc-47p::GFP* intensities, rectal cell shapes. The percentage of animals (n%) with the corresponding landmarks is indicated at each particular time point. Worms were synchronized by hatch pulse (see Methods). Left is towards front, anterior is left, and dorsal is up.

(B) Quantification of worms with expression of *fpEx1062[let-413::gfp::pest*], *fpIs110[lin-26p::GFP]* and *oxIs12[unc-47p::GFP]* in K.p over time, in L1 and L2 grown at 20°C. n, total animal scored.

(C) *lin-26* smFISH staining (white spots on the merge) on 26h post hatching L1 larvae expressing a *gfp::sox-2* (CRISPR KI *syb737*, shown in cyan) and the rectal nuclear reporter *gaIs245*, shown in magenta. Intestinal cells and Pn.p cells in the same area were identified with the DAPI staining, in yellow. Scale bar for all in bottom right picture.

(D) Quantifications of the *lin-26* smFISH spots were performed on K.p and F rectal cells as well as on intestinal cells (Int 8/9) and Pn.p cells (P9/10.p). We considered only the spots in contact with the nuclei to reflect the most recently transcribed mRNAs. Statistical test compares the number of spots in the K.p cell to the others. Very few *lin-26* mRNA molecules are detected in close proximity to the nucleus in epithelial cells generally (from 3 spots to 5 spots on average; P9/10.p where *lin-26* has been shown to be expressed (Labouesse et al., 1996), 3 spots; rectal F cell, 5 spots; K.a, which is on the left side, was very difficult to image because of photobleaching (n=9) and exhibits 4 spots on average). Intestinal cells, where *lin-26* is not expressed, show no *lin-26* mRNA spots (0, 22 spots on average).

Fig. S3.

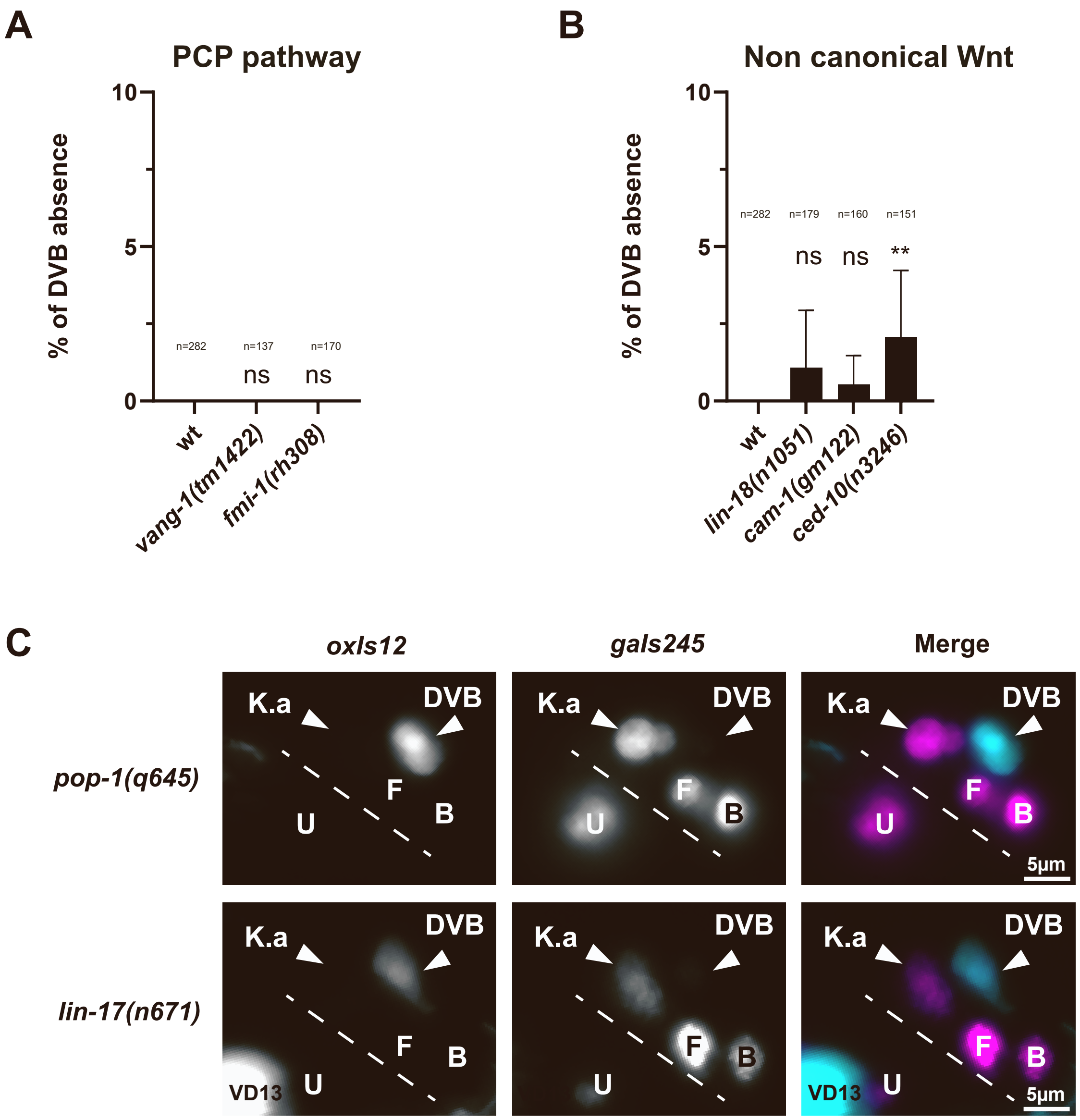

Figure S3. The non-canonical Wnt pathways are not required for K-to-DVB. Related to Figure 3.

(A) Histograms showing the percentage of "No DVB" worms in mutant backgrounds for genes of the PCP pathway.

(B) Histograms showing the percentage of "No DVB" worms in mutant backgrounds for the non-canonical Wnt-dependent pathways (*lin-18* and *cam-1*) or their downstream effectors (*ced-10*). n, total animal scored;.ns, non-statistically significant; **, p<0.005.

(C) When present, in a small percentage of *pop-1/TCF* (top) or *lin-17/FZD* (bottom) L4 mutants, the DVB neuron is formed from K posterior daughter. The positions of DVB, as observed with o*xIs12,* and of the K.a, U, F and B rectal cells, as observed using *gaIs245*, are indicated. VD13, GABAergic neuron. Dash line, rectal slit. Anterior is to the left and ventral to the bottom.

Fig. S4.

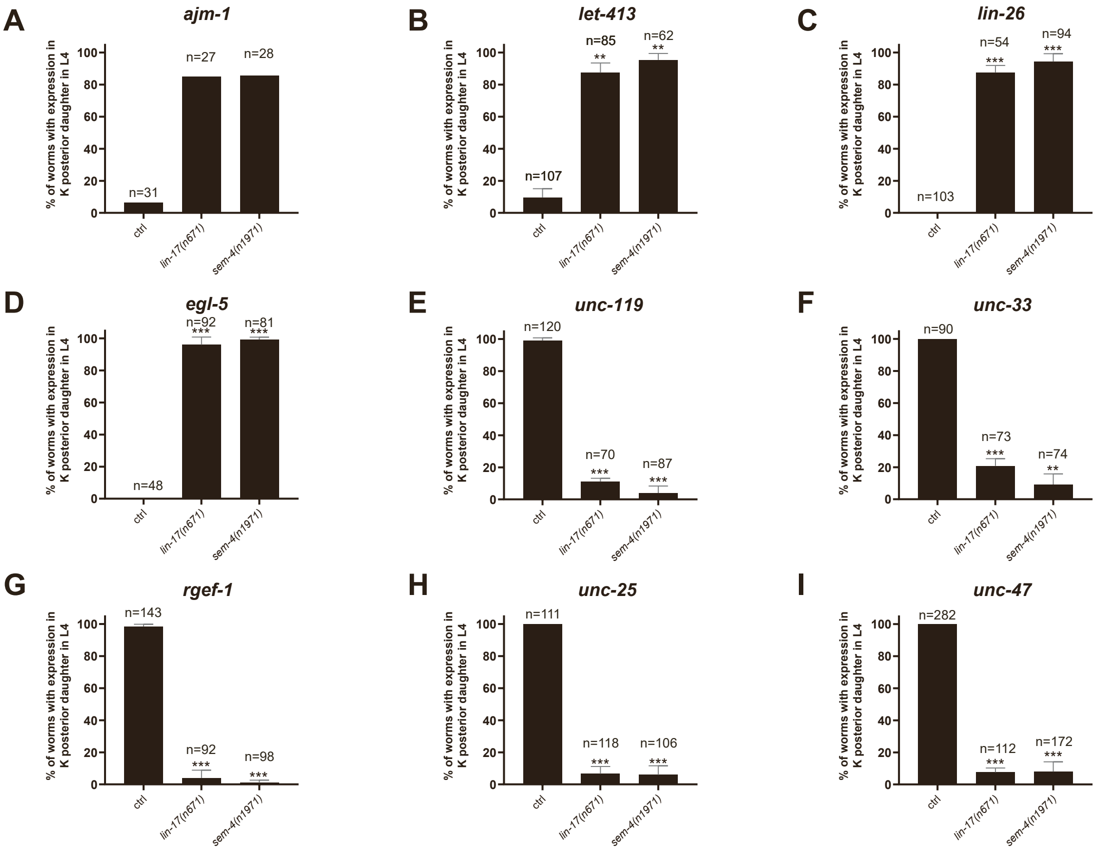

Figure S4. K.p cell remains rectal-epithelial in *lin-17/FZD* and *sem-4/SALL* mutants. Related to Figure 3 and Figure 4.

Quantification of the % of animals expressing (**A-C**) epithelial (*ajm-1, let-413* and *lin-26*), (**D**) rectal (*egl-5*), (**E-G**) pan-neuronal (*unc-119*, *unc-33* and *rgef-1*) and (**H, I**) GABAergic (*unc-25, unc-47*) reporters in K posterior daughter in *lin-17/F*ZD and *sem-4/SALL* mutant backgrounds, or DVB in wild type, in L4 larvae. n, total animal scored.

Fig. S5.

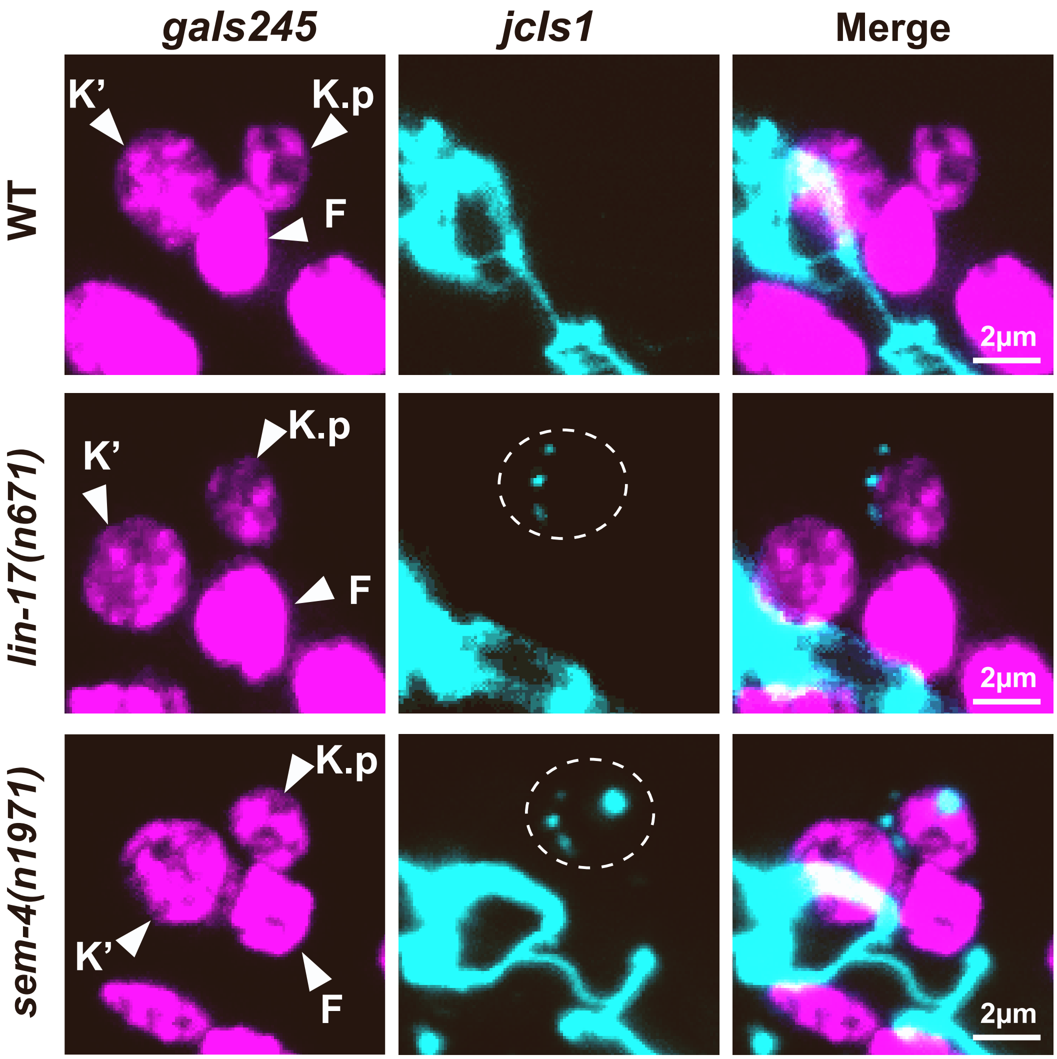

Figure S5. K.p cell expresses the apical junction protein AJM-1 in *lin-17/FZD* and *sem-4/SALL* mutants. Related to Figure 3 and Figure 4.

Confocal images of wild-type, *lin-17/FZD* and *sem-4/SALL* mutant backgrounds in L3 larvae carrying *gaIs245[col-34p::his-24::mcherry]* to visualize the rectal cell nuclei and *jcIs1[ajm-1::GFP]*. Patches of AJM-1 proteins are present in the K.p cell (dashed oval) in the mutant backgrounds, consistently with the mutant K.p retaining its epithelial identity.

Fig. S6.

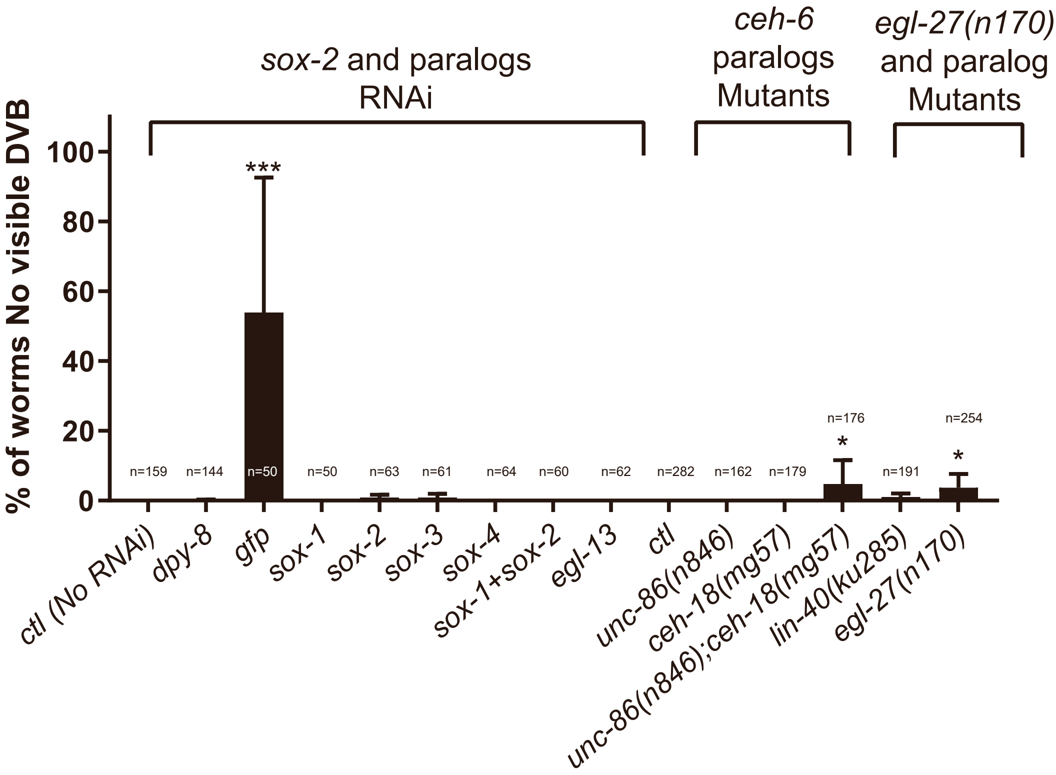

Figure S6. *sox-2*, *ceh-6* and *egl-27* paralogs do not seem to be required to form DVB. Related to Figure 4. Quantification of DVB defective L4 animals (as observed by *unc-47* expression) using RNAi in a sensitized *rrf*-3 mutant background to target *sox-2* paralogs (*dpy-8* and *gfp* RNAi represent controls). Mutants were used for paralogs of *ceh-6* (*unc-86(n846)* and *ceh-18(mg57)*) and *egl-27* (*lin-40(ku285)*). No obvious defects were observed, although RNAi was found to work poorly in the rectal cells. n, total animal scored.

Fig. S7.

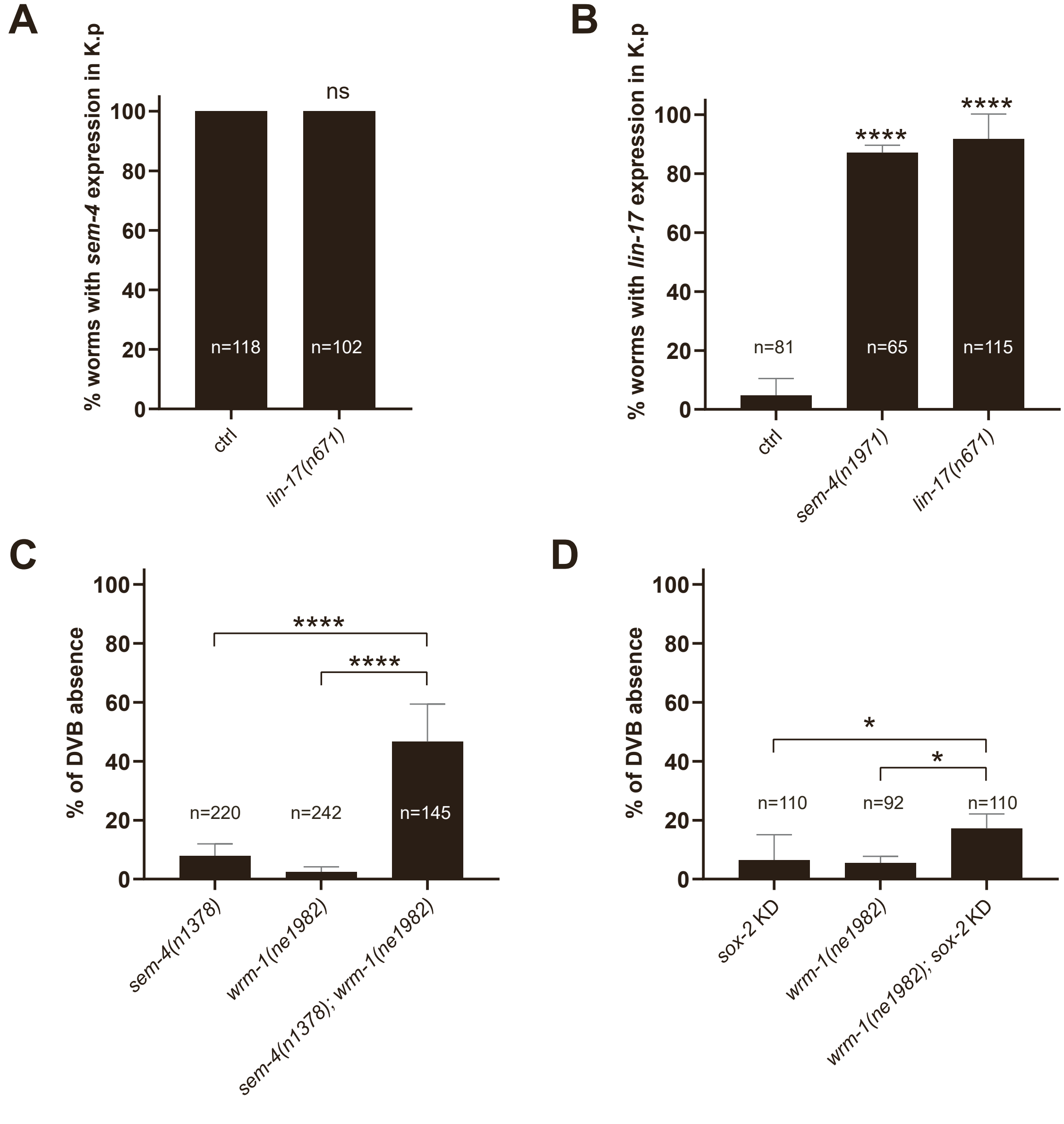

Figure S7. *sem-4/SALL* and the Wnt signaling pathway act in parallel to drive K-to-DVB Td. Related to Figure 5.

(A) Quantification of *sem-4/SALL* expression in K.p in wild type L4s and in *lin-17/Frizzled* mutant L4s.

(B) Quantification of *lin-17/FZD* expression in K.p in wild type L4s *vs sem-4/SALL* and *lin-17/FZD* mutants.

(C-D) Quantification of DVB defective L4 animals (as observed by *unc-47* expression using *krIs6* in A and *oxIs12* in B) in simple *sem-4(n1378)* (C), *sox-2* knock-down (using a nanobody strategy, D) and *wrm-1(n1982)* (C, D) mutants, or in *sem-4(n1378);wrm-1(n1982)* (C) and *wrm-1(n1982);sox-2* KD (D) double mutants, all raised at 25°C. n, total animal scored.

Fig. S8.

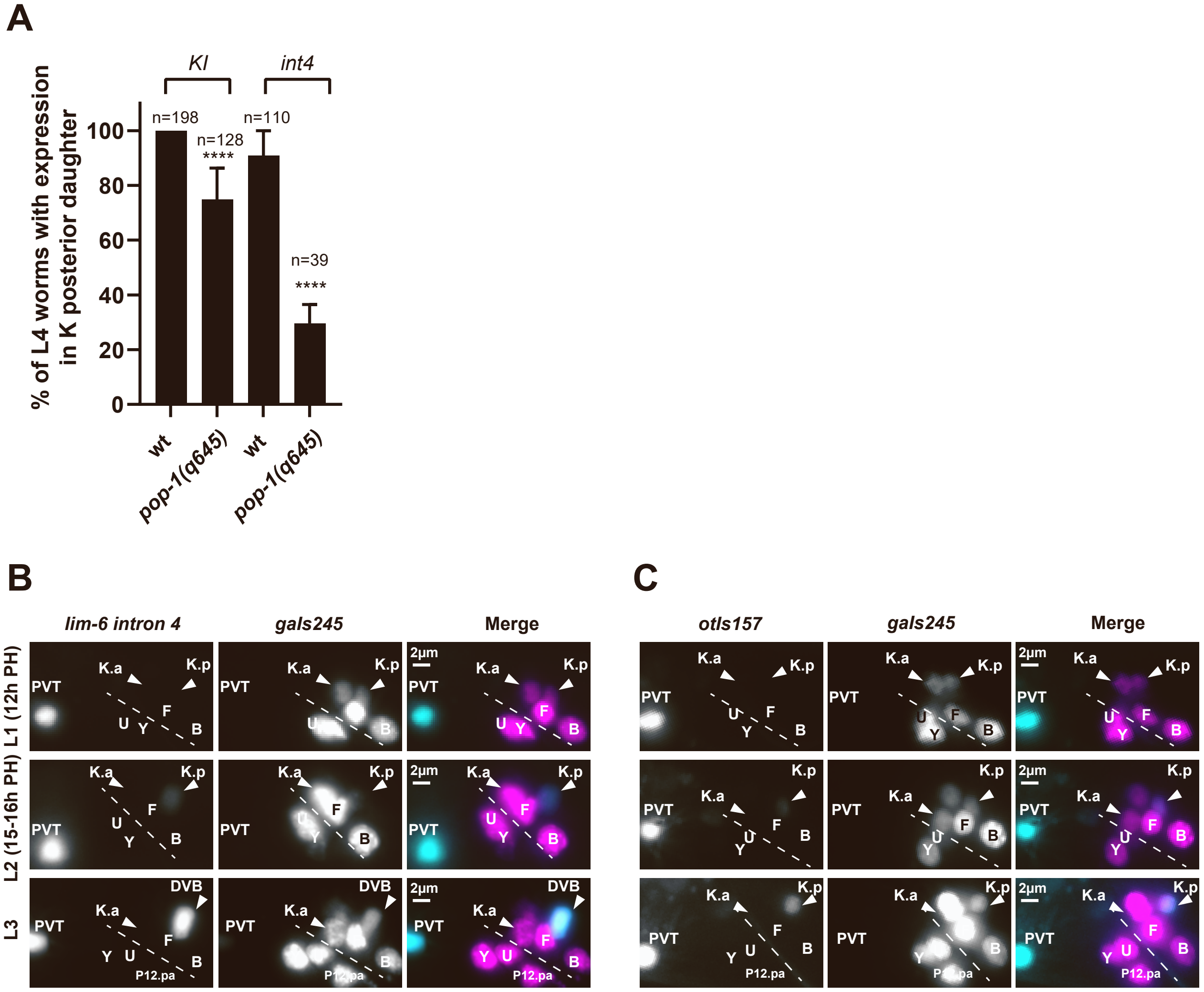

Figure S8. ***lim-6*** **is expressed early in K.p and its expression is affected in** ***pop-1/TCF*** **mutant. Related to Figure 5.**

(A) *lim-6* expression is impaired in *pop-1* mutant. Quantification of the % of L4 larvae expressing *lim-6::gfp* CRISPR (KI) and *lim-6 intron 4* transcriptional reporter (*int4*) in wild-type (DVB) and *pop-1(q645)* mutant (persistent K.p) backgrounds. Note that for the *pop-1(q645)* mutant, only viable homozygote (not balanced) mutant worms were analyzed. n, total animal scored.

(B-C) Time course expression of *lim-6(int4)::gfp (fpEx1111)* (B) and *lim-6r::gfp (otIs157)* (C) reporters in K.p/DVB (Cyan) in L1, 1h after the division (top), in an early L2 animal (middle) and in an L3 larva (bottom) where rectal cells are visualized with *gaIs245 (col-34p::his-24::mcherry;* magenta*)*.

For all pictures, dashed line, rectal slit. Anterior is to the left and ventral to the bottom.

Fig. S9.

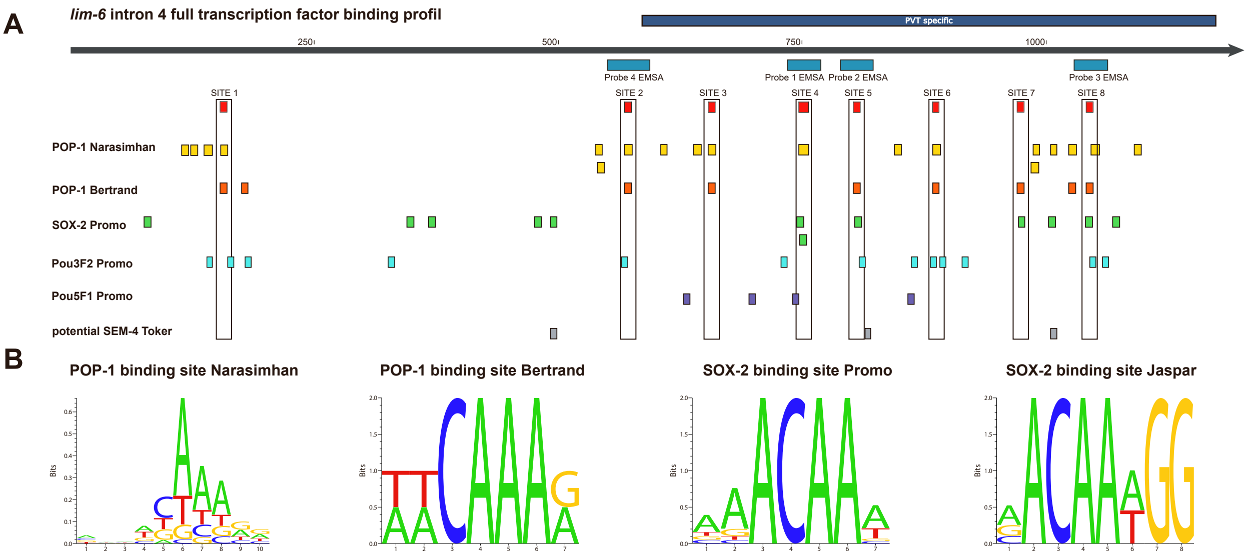

C

Site	Sequence	1st nucleotide Position in intron 4 (bp)	Identification method	Mutation in pSJ759
Site 1	TTCAAAG	154	Fimo: Narasimhan, 2015<br>Bertrand, 2009<br>MatInspector	
Site 2	ATCAAAA	569	Fimo: Narasimhan, 2015<br>Bertrand, 2009<br>MatInspector	ATCGTTA
Site 3	TTCAAAG	655	Fimo: Narasimhan, 2015<br>Bertrand, 2009<br>MatInspector	TTCGTTG
Site 4	ACAACAAACA	748	Fimo: Narasimhan, 2015	ACACCATGGA
Site 5	CTTTGTT	804 (rev)	Fimo: Narasimhan, 2015<br>Bertrand, 2009<br>MatInspector	CGCTAGC
Site 6	ATCAAAG	885	Fimo: Narasimhan, 2015<br>Bertrand, 2009<br>MatInspector	ATCCTCG
Site 7	CTTTGTT	972 (rev)	Bertrand, 2009<br>MatInspector	CCCGCGG
Site 8	AACAAAA	1043	Bertrand, 2009<br>MatInspector	CGGCCGA

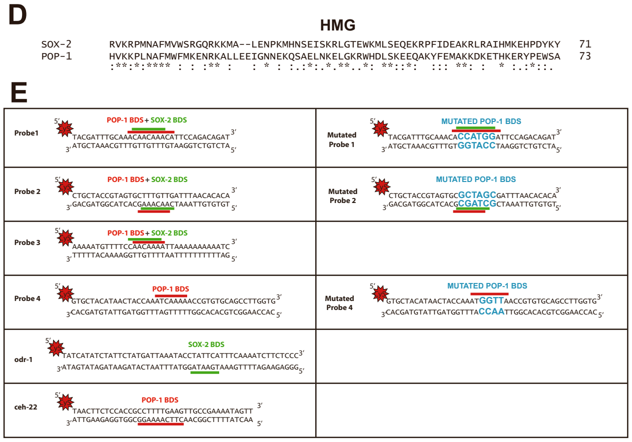

Figure S9. Analysis of SOX-2 and POP-1/TCF binding sites in *lim-6* intron 4. Related to Figure 5.

(A) The intron 4 of *lim-6* was analyzed using different tools (See Methods). Here the POP-1 binding site prediction using the Matrix of Narasimhan and the consensus of Bertrand, 2009, are represented. The SOX-2, POU3F2 and POU5F1 binding sites were predicted by Promo and the SEM-4 consensus site published in Toker, 2003 was used.

(B) Sequence logo for POP-1 and SOX-2 binding sites showing the sequence similarities.

(C) Table summarizing the binding sites on which we have focused our efforts in this study. Binding sites predicted by more than one approach, or because of the presence of two consecutive binding sites for SOX-2 (site 4), were selected. The mutations introduced into the *lim-6* transcriptional reporter to abolish POP-1 binding are presented on the right. Note that these mutations most probably abolish also SOX-2 binding due to the very close similarity of their predicted binding sites.

(D) This binding site similarity can be explained by the sequence similarity of the HMG domains present in SOX-2 and POP-1.

(E) Sequence of the probes used in this study for the gel shift experiments. Note that probe 3 displayed poor annealing due to its AT rich sequence and therefore was not further used.

Fig. S10.

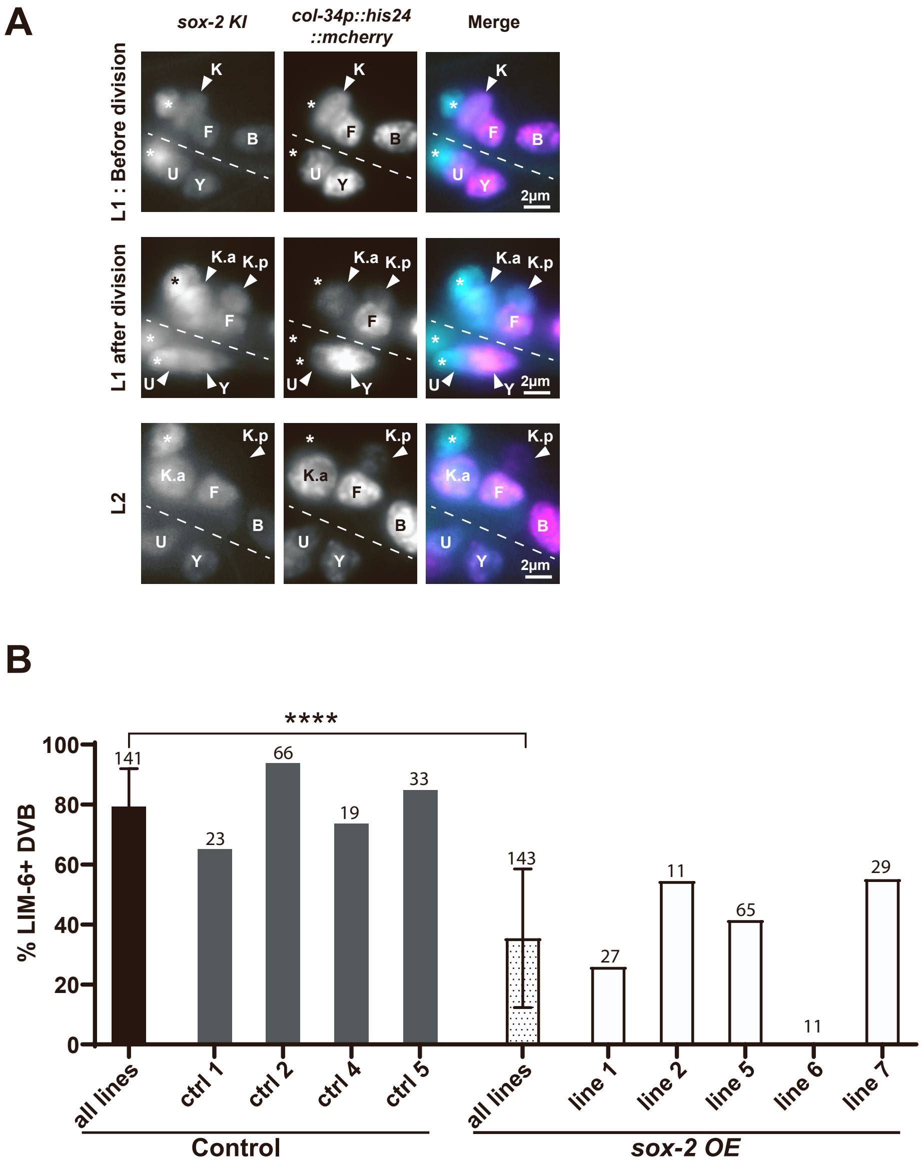

Figure S10. ***sox-2*** **is expressed in K.p after K division and it is subsequently downregulated during DVB differentiation. Overexpression of** ***sox-2*** **prevents** ***lim-6*** **expression. Related to Figure 5A.**

(A) Fluorescent images of *gfp::sox-2* KI and *col-34p::his-24::mcherry* in the rectum of a wild-type L1 animal before K division (top), in an L1 animal after K division (14 cells in the gonad; middle) and in an L2 animal (bottom). Note that K.a continues to express *sox-2* over time whereas expression fades away in K.p during its conversion. White stars indicate the rectal gland cells; the rectal cell position is indicated on the pictures; dashed line, rectal slit; anterior is left and ventral is bottom.

(B) The rectal *col-34* promoter was used to overexpress (OE) SOX-2 in K.p along with a co-injected *lim-6(int4)::mCherry* reporter. The % of L4 animals displaying *lim-6* expression in DVB are represented. Black bar, all results obtained for the control lines (*lim-6(int4)::mCherry* alone) and dark grey bars, each individual line data respectively; Dotted bar, all results obtained for the SOX-2 overexpressing lines, followed by each individual SOX-2(OE) line data (white bars). Note that transgenic lines overexpressing SOX-2 are difficult to retrieve and maintain, and throw few transgenic animals: transgenics in the F2 generation were usually the only animals that could be scored. The total number of animals scored is indicated above each bar. ****, $p<0.0001$.

Fig. S11.

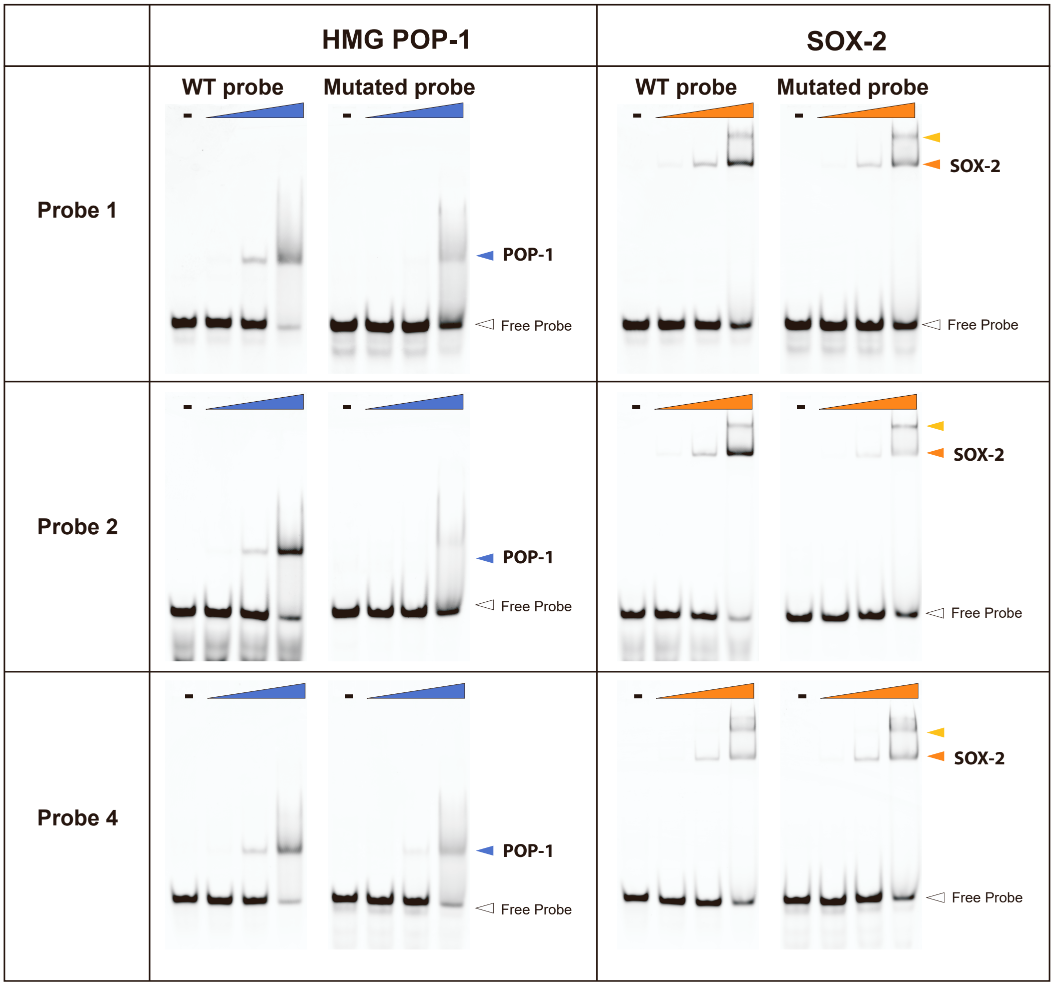

Figure S11. Gel shift experiments show independent binding capacities of POP-1 and SOX-2 to probes 1, 2 and 4. Related to Figure 5E, F.

Increasing concentration (5nm, 50nM and 500nM) of purified HMG-POP-1 and SOX-2 were incubated with wild type or mutated probes 1, 2 and 4 bound to the Cy5 fluorophore. Note that the probe 4 which does not bear canonical SOX-2 binding site is able to bind SOX-2. As, in addition, the mutation of the POP-1 binding site does not seem to affect this binding, it is likely that a non-predicted SOX-2 binding site is present. Probe 3 was also able to bind both SOX-2 and HMG-POP-1, although results for are not presented because this probe annealed poorly, most probably due to its AT-rich sequence. Blue arrowhead, POP-1 bound to the probe; orange arrowhead, SOX-2 bound to the probe; light orange arrowhead, a second SOX-2 shifted band appears at high SOX-2 concentrations; open arrowhead, unbound probe.

Fig. S12.

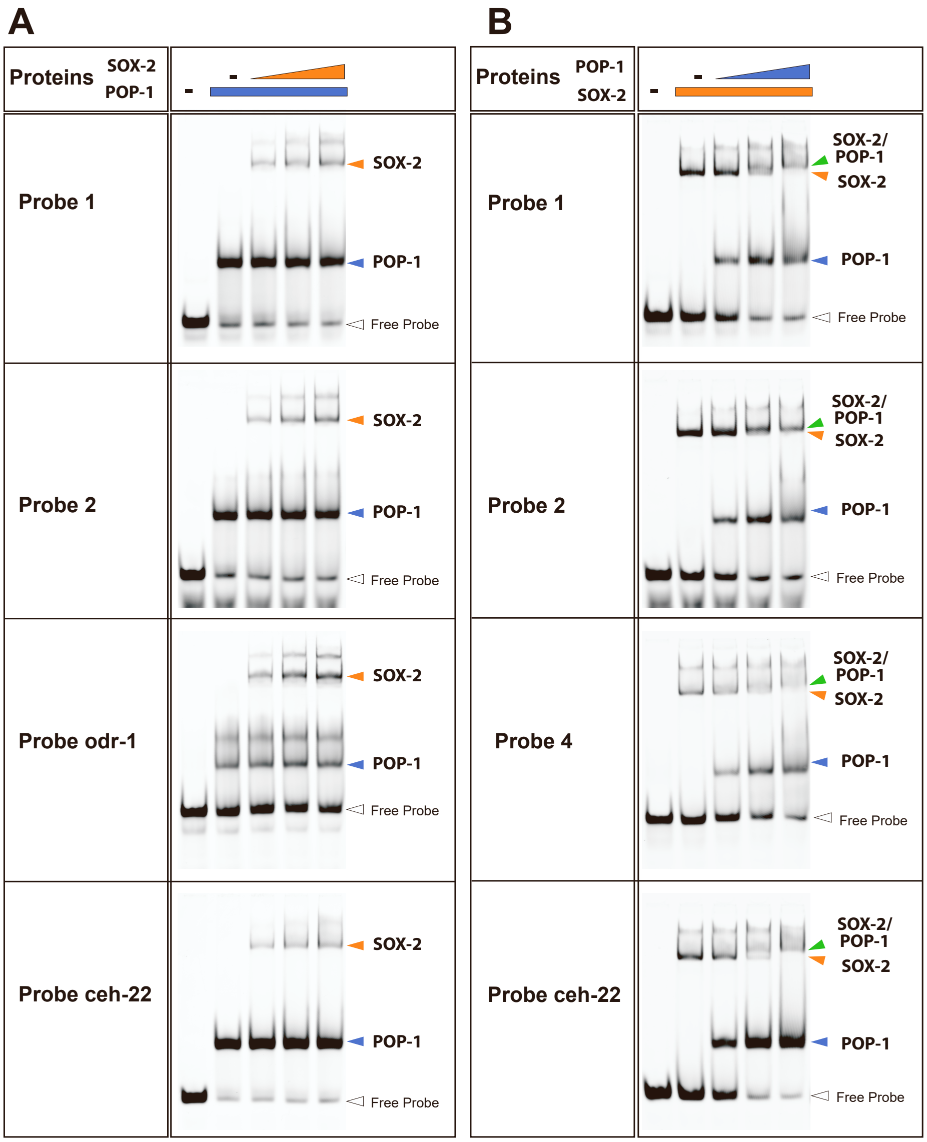

Figure S12. Gel shift experiments show binding capacities of POP-1 and SOX-2 when co-incubated. Related to Figure 5E, F.

(**A**) Increasing quantity of SOX-2 (125nM-250nM-500nM) was added to a mix of HMG-POP-1 and Cy-5-dsProbe #1, #2, *odr-1* (known SOX-2 target, Alqadah, 2015) and *ceh-22* (known POP-1 target, Lam, 2007, Bhambhani, 2014). (**B**) Increasing quantity of HMG-POP-1 (125nM-250nM-500nM) was added to a mix of SOX-2 and Cy-5-dsProbe #1,# 2, #4 and *ceh-22*. Increasing quantity of HMG-POP-1 shows an increasing binding to all the probes as well as an upper shift, most probably corresponding to a HMG-POP-1-SOX-2-Probe complex.

Blue arrowhead, POP-1 bound to the probe; orange arrowhead, SOX-2 bound; green arrowhead, POP-1 and SOX-2 bound; open arrowhead, unbound probe.

Fig. S13.

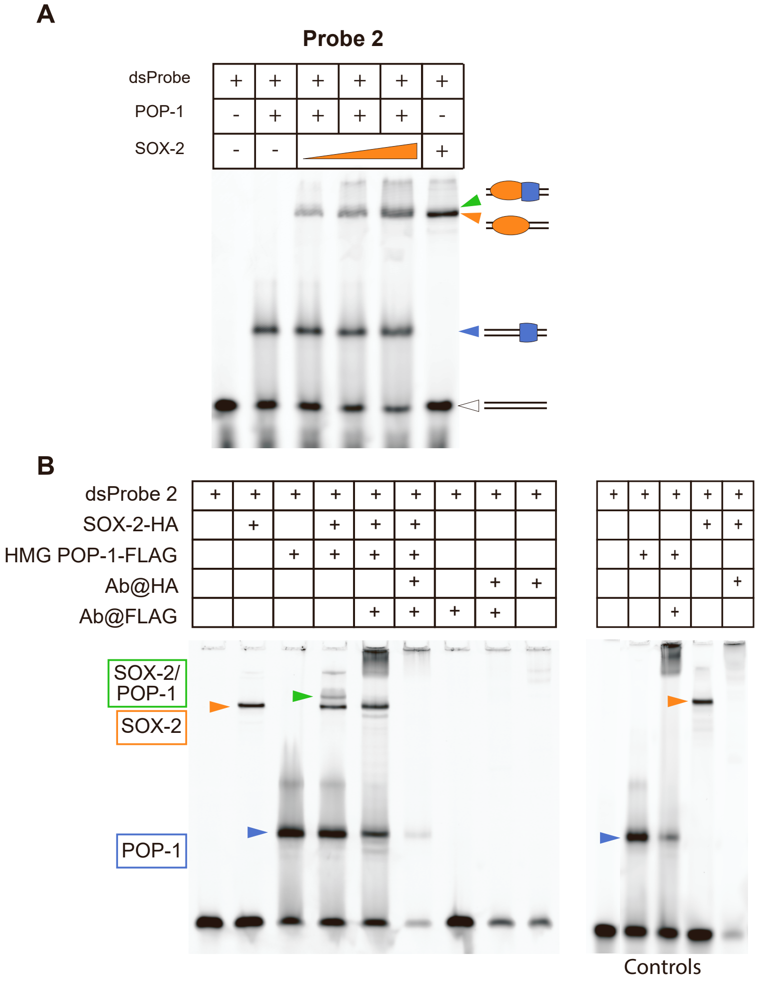

Figure S13. Antibody supershift EMSA analysis of SOX-2 and POP-1 co-binding. Related to Figure 5E, F.

(**A**) Representative EMSA assay on Probe 2 revealing single binding of SOX-2 (orange arrow head) and HMG-POP-1 (blue arrow head) as well as co-binding (upper band, green arrow head).

(**B**) This upper band was totally upshifted after pre-incubation of the complex with an anti-FLAG antibody (Ab@FLAG) against HMG-POP-1-FLAG.

Various combinations of antibodies/protein/probe complexes were used for controls as indicated.

Table S1. Summary of all cell markers expression.

C. elegans genes	Human ortholog	Reporter	WT				*sem-4*	*lin-17*	References reporter	Ref observation
			L1	early L2		L4	L4	L4		
			K	K.a	K.p	DVB	K post. daughter	K post. daughter		
**Epithelial markers**										
dlg-1	*DLG*	*mcIs46[dlg-1::rfp]*	+	+	-	-	N.D	N.D	Diogon et al. (2007)	This study
ajm-1	*AJM1*	*jcIs1[ajm-1::gfp]*	+	+	-	-	+	+	Mohler et al. (1998)	This study; Mohler et al. (1998)
hmr-1	Cadherin	*fpIs17[hmr-1::gfp]*	+	+	-	-	N.D	N.D	This study	This study
let-413	*SCRIB*	*fpEx1062[let-413a::gfp::pest]*	+	+	+	-	+	+	This study	This study
lin-26	Zinc-finger transcription factor	*fpIs110[lin-26rectalp::gfp]*	+	+	+	-	+	+	Labouesse et al. (1996); this study	Labouesse et al. (1996); this study
**Rectal markers (also in other cells, to visualise the rectal cells)**										
sem-4	*SALL* transcription factor	*syb1287[sem-4::gfp]*	+	+	+	+	ND	+	This study	This study
sox-2	*SOX* transcription factor	*syb737[gfp::sox-2]*	+	+	+*	-	+	+	This study	This study
ceh-6	*POU* transcription factor	*syb972[gfp::ceh-6]*	+	+	+*	-	+	+	This study	This study
egl-5	*HOX* transcription factor	*bxIs7[egl-5::gfp]*	+	+	+	-	ND	ND	Teng et al. (2004)	This study
col-34	Cuticle collagen gene	*gaIs245[col-34p::his-24::mcherry]*	+	+	+	-	+	+	Zuryn et al. (2014)	This study
got-1.2	*GOT1*	*sIs11174[rCesT01C8.5::gfp+p Ceh361]*	+	+	+	-	ND	ND	McKay et al. (2003)	This study
**Pan-neuronal markers**										
unc-33	*DPYS*	*otIs117[unc-4(+) ; unc-33p::GFP]*<br>*otIs118[unc-33::GFP; unc-4(+)]*	-	-	-	+	-	-	McKay et al. (2003)	This study
unc-119	*UNC119*	*edIs6[unc-119::gfp; rol-6]*	-	-	-	+	-	-	Maduro and Pilgrim (1995); Praitis et al. (2001)	This study
rgef-1	RASGRP3	*otIs173 [F25B3.3::DsRed2; ttx-3promB::GFP]*	-	-	-	+	-	-	Benard et al. (2009)	This study
**DVB Terminal selector**										
lim-6	*LMX1B*	*syb971[lim-6::gfp]*<br>*otIs157[lim-6r::GFP]*	-	-	+	+	-	-	This study	Hobert et al. (1999); this study
**GABAergic markers**										
unc-47	*SLC32A1*	*oxIs12[unc-47p::gfp]*<br>*krIs6[unc-47p::DsRed2]*	-	-	-	+	-	-	McIntire et al. (1997)<br>Teuliere et al. (2011)	McIntire et al. (1997); this study<br>Teuliere et al. (2011)
unc-25	*GAD*	*juIs8[unc-25p::gfp]*	-	-	-	+	-	-	Jin et al. (1999)	Jin et al. (1999) ; this study

*, expression is seen in K.p after its birth, and disappears as *lim-6* expression appears (see Fig. 5A).

Table S2. Strain list.

C. elegans strain	Identifier
rrf-3(pk1426) II ; oxIs12[unc-47::gfp; lin-15(+)] X	IS17
lin-5(ev571) II; gaIs245[col-34p::his-24::mcherry; unc-119(+)] V ; oxIs12[unc-47p::gfp; lin-15(+)] X	IS1118
sem-4(n1971) bxIs7[egl-5p(6,5kb)::gfp; lin-15(+)] I; otIs173[rgef-1p::dsred2; ttx-3p::gfp] III	IS1208
sem-4(n197) I; otIs117[unc-4(+); unc-33p::gfp] IV; gaIs245[col-34p::his-24::mcherry; unc-119(+)] V	IS1210
gaIs245[col-34p::his-24::mcherry; unc-119(+)] V; oxIs12[unc-47p::gfp; lin-15(+)] X	IS1299
egl-5(n945) III; gaIs245[col-34p::his-24::mcherry; unc-119(+)] V; oxIs12[unc-47p::gfp; lin-15(+)] X	IS1332
lin-17(n671) I; gaIs245[col-34p::his-24::mcherry; unc-119(+)] V; oxIs12[unc-47p::gfp; lin-15(+)] X	IS1370
fpIs17[hmr-1::gfp]; gaIs245[col-34p::his-24::mcherry; unc-119(+)] V	IS1374
wrm-1(ne1982) III; gaIs245[col-34p::his-24::mcherry; unc-119(+)] V; oxIs12[unc-47p::gfp; lin-15(+)] X	IS1432
sem-4(n1971) I; gaIs245[col-34p::his-24::mcherry; unc-119(+)] V; oxIs12[unc-47p::gfp; lin-15(+)] X	IS2968
unc-86(n846) III; gaIs245[col-34p::his-24::mcherry; unc-119(+)] V; oxIs12[unc-47p::gfp; lin-15(+)] X	IS3097
fpIs110[lin-26p::gfp; rol-6(su1006)] IV; gaIs245[col-34p::his-24::mcherry; unc-119(+)] V	IS3107
egl-27(ok1670) II; gaIs245[col-34p::his-24::mcherry; unc-119(+)] V ; oxIs12[unc-47p::gfp; lin-15(+)] X	IS3113
gaIs245[col-34p::his-24::mcherry; unc-119(+)] V; fpEx1062[let-413a::gfp::pest; myo-2p::gfp]	IS3119
gaIs245[col-34p::his-24::mcherry; unc-119(+)] V; oxIs12[unc-47p::gfp; lin-15(+)] X; fpEx955[Δ(-2846pb to -102)ceh-6p::gfp::ceh-6; odr-1::rfp]	IS3120
ceh-6(gk665) I; gaIs245[col-34p::his-24::mcherry; unc-119(+)] V; oxIs12[unc-47p::gfp; lin-15(+)] X; fpEx955[Δ(-2846pb to -102)ceh-6p::gfp::ceh-6; odr-1::rfp]	IS3122
gaIs245[col-34p::his-24::mcherry; unc-119(+)] V; oxIs12[unc-47p::gfp; lin-15(+)] X; fpEx788[egl-5p(1,3kb)::sox-2(antisens); rol-6(su1006)]	IS3142
lin-40(ku285) V; oxIs12[unc-47p::gfp; lin-15(+)] X	IS3146
sem-4(n1971) I; gaIs245[col-34p::his-24::mcherry; unc-119(+)] V; fpEx1062[let-413a::gfp::pest; myo-2p::gfp]	IS3176
gaIs245[col-34p::his-24::mcherry; unc-119(+)] V; juIs8[unc-25p::gfp; lin-15(+)]	IS3298
edIs6[unc-119p::gfp; rol-6(su1006)] IV; gaIs245[col-34p::his-24::mcherry; unc-119(+)] V	IS3327
lin-17(n671)I; edIs6[unc-119p::gfp; rol-6(su1006)] IV; gaIs245[col-34p::his-24::mcherry; unc-119(+)] V	IS3328

gaIs245[col-34p::his-24::mcherry; unc-119(+)] V; otIs118[unc-33p::gfp; unc-4(+)]	**IS3329**
lin-17(n671)I; gaIs245[col-34p::his-24::mcherry; unc-119(+)] V; otIs118[unc-33p::gfp; unc-4(+)]	**IS3330**
lin-17(n671)I; gaIs245[col-34p::his-24::mcherry; unc-119(+)] V; juIs8 [unc-25p::gfp; lin-15(+)]	**IS3335**
jcIs1[ajm-1::gfp; rol-6(su1006)] IV; gaIs245[col-34p::his-24::mcherry; unc-119(+)] V	**IS3339**
lin-17(n671) I; fpIs110[lin-26p::gfp; rol-6(su1006)] IV; gaIs245[col-34p::his-24::mcherry; unc-119(+)] V	**IS3349**
lin-17(n671) I; jcIs1[ajm-1::gfp; rol-6(su1006)] IV; gaIs245[col-34p::his-24::mcherry; unc-119(+)] V	**IS3357**
gaIs245[col-34p::his-24::mcherry; unc-119(+)] V; fpEx1111[lim-6int4::gfp; coel::dsred]	**IS3379**
lin-17(n671) I; gaIs245[col-34p::his-24::mcherry; unc-119(+)] V; fpEx1062[let-413a::gfp::pest; myo-2p::gfp]	**IS3383**
lin-17(n671) I; gaIs245[col-34p::his-24::mcherry; unc-119(+)] V; fpEx1111[lim-6int4::gfp; coel::dsred]	**IS3420**
gaIs245[col-34p::his-24::mcherry; unc-119(+)] V; sox-2(syb737[gfp::linker::sox-2]) X	**IS3423**
wyIs75[unc-47p::dsred; exp-1p::gfp; odr-1p::rfp] III; vang-1(tm1422)X	**IS3433**
unc-73(e936) dpy-5(e61) I ; gaIs245[col-34p::his-24::mcherry; unc-119(+)] V ; oxIs12[unc-47p::gfp; lin-15(+)] X	**IS3452**
lin-17(n671) I; gaIs245[col-34p::his-24::mcherry; unc-119(+)] V; sox-2(syb737[gfp::linker::sox-2]) X	**IS3457**
sem-4(n1971) I; gaIs245[col-34p::his-24::mcherry; unc-119(+)] V; sox-2(syb737[gfp::linker::sox-2]) X	**IS3458**
dsh-1(ok1445) II; gaIs245[col-34p::his-24::mcherry; unc-119(+)] V; oxIs12[unc-47p::gfp; lin-15(+)] X	**IS3464**
egl-27(ok1670) II; wyIs75[unc-47p::dsred; exp-1p::gfp; odr-1p::rfp] III; him-5(e1490)V	**IS3469**
egl-5(n945) III; syIs50[cdh-3p::gfp; dpy-20(+)]	**IS3475**
egl-20(n585) IV ; gaIs245[col-34p::his-24::mcherry; unc-119(+)] V; oxIs12[unc-47p::gfp; lin-15(+)] X	**IS3485**
lin-44(n1792)I; gaIs245[col-34p::his-24::mcherry; unc-119(+)] V; oxIs12[unc-47p::gfp; lin-15(+)] X	**IS3486**
lin-44(n1792) I ; egl-20(n585) IV ; gaIs245[col-34p::his-24::mCherry; unc-119(+)] V ; oxIs12[unc-47p::gfp; lin-15(+)] X	**IS3487**
gaIs245[col-34p::his-24::mcherry; unc-119(+)] V; lim-6(nr2073) oxIs12[unc-47p::gfp; lin-15(+)] X	**IS3490**
par-1(zu310) gaIs245[col-34p::his-24::mcherry; unc-119(+)] V; oxIs12[unc-47p::gfp; lin-15(+)] X	**IS3491**
fmi-1(rh308) gaIs245[col-34p::his-24::mcherry; unc-119(+)] V; oxIs12[unc-47p::gfp; lin-15(+)] X	**IS3511**
dsh-1(ok1445) mig-5(tm2639) II; oxIs12[unc-47p::gfp; lin-15(+)] X	**IS3512**
wyIs75[unc-47p::dsred; exp-1p::gfp; odr-1p::rfp] III; gaIs245[col-34p::his-24::mcherry; unc-119(+)] V; sox-2(syb737[gfp::linker::sox-2]) X; fpEx1156[egl-5p(6.5kb)::nanobodyGFP::zif-1; coel::gfp; pBSK]	**IS3521**

gpr-1(ok2126) III; gaIs245[col-34p::his-24::mcherry; unc-119(+)] V; oxIs12[unc-47p::gfp; lin-15(+)] X	**IS3530**
sem-4(n1971)I; edIs6[unc-119p::gfp; rol-6(su1006)] IV; gaIs245[col-34p::his-24::mcherry; unc-119(+)] V	**IS3537**
sem-4(n1971)I; gaIs245[col-34p::his-24::mcherry; unc-119(+)] V; otIs118[unc-33p::gfp; unc-4(+)]	**IS3539**
gaIs245[col-34p::his-24::mcherry; unc-119(+)] V; ceh-6(syb972[gfp::linker::ceh-6]) X	**IS3540**
lin-17(n671) bxIs7[egl-5(6.5kb)::gfp; lin-15(+)] I; otIs173[rgef-1p::dsred2; ttx-3pB::gfp]III	**IS3583**
hT2[bli-4(e937) let-?(q782) qIs48] (I;III)/pop-1(q645)I ; gaIs245[col-34p::his-24::mcherry; unc-119(+)] V; oxIs12[unc-47p::gfp; lin-15(+)] X	**IS3596**
hT2[bli-4(e937) let-?(q782) qIs48] (I;III)/pop-1(q645)I; gaIs245[col-34p::his-24::mcherry; unc-119(+)] V; fpEx1111[lim-6int4::gfp; coel::dsred]	**IS3600**
otIs173[rgef-1p::dsred2; ttx-3pB::gfp]III; oxIs12[unc-47p::gfp; lin-15(+)] X	**IS3604**
lin-5(ev571)II; gaIs245[col-34p::his-24::mcherry; unc-119(+)] V; fpIs101[col-34p::ph::gfp; odr-1p::dsRed] X	**IS3619**
sem-4(n1971)I; gaIs245[col-34p::his-24::mcherry; unc-119(+)] V; lim-6(syb971[lim-6::linker::gfp])X	**IS3632**
lin-17(n671)I; gaIs245[col-34p::his-24::mcherry; unc-119(+)] V; lim-6(syb971[lim-6::linker::gfp])X	**IS3669**
gaIs245[col-34p::his-24::mcherry; unc-119(+)] V; lim-6(syb971[lim-6::linker::gfp])X	**IS3677**
lin-17(n671) I; gaIs245[col-34p::his-24::mcherry; unc-119(+)] V; ceh-6(syb972[gfp::linker::ceh-6]) X	**IS3702**
sys-1(q544) I; gaIs245[col-34p::his-24::mcherry; unc-119(+)] V; oxIs12[unc-47p::gfp; lin-15(+)] X	**IS3718**
krIs6[unc-47::DsRed2; lin-15(+)] II ; gaIs245[col-34p::HIS-24::mCherry; unc-119(+)] V; fpEx1295(pcr fragment col-34p::gfp::cki-1(gDNA), myo-2p::mCherry]	**IS3950**
krIs6[unc-47::DsRed2; lin-15(+)] II ; gaIs245[col-34p::HIS-24::mCherry; unc-119(+)] V; fpEx1296(pcr fragment col-34p::gfp::cki-1(gDNA), myo-2p::mCherry]	**IS3951**
krIs6[unc-47::DsRed2; lin-15(+)] II ; gaIs245[col-34p::HIS-24::mCherry; unc-119(+)] V; fpEx1297(pcr fragment col-34p::gfp::cki-1(cDNA), myo-2p::mCherry]	**IS3952**
krIs6[unc-47::DsRed2; lin-15(+)] II ; gaIs245[col-34p::HIS-24::mCherry; unc-119(+)] V; fpEx1298(pcr fragment col-34p::gfp::cki-1(gDNA), myo-2p::mCherry]	**IS3972**
krIs6[unc-47::DsRed2; lin-15(+)] II ; gaIs245[col-34p::HIS-24::mCherry; unc-119(+)] V; fpEx1299(pcr fragment col-34p::gfp::cki-1(gDNA), myo-2p::mCherry]	**IS3973**
krIs6[unc-47::DsRed2; lin-15(+)] II ; gaIs245[col-34p::HIS-24::mCherry; unc-119(+)] V; fpEx1300(pcr fragment col-34p::gfp::cki-1(gDNA), myo-2p::mCherry]	**IS3974**

Table S3. Oligonucleotides list.

Oligo name	Sequence	Use
**BDT950**	CTGAATCCGGATCCATCATCATGTCGACTGCAGAATTCGAAGCTT GTCGACGGAGCTC	*sox-2* antisense construct
**BDT952**	CTTGGAGGGTACCTAGGAGCTCGATATCTAGAAGAGGTAACATG GGATTGGGA	*sox-2* antisense construct
**EB110F**	AGAAGACCGCCCCTCTTTTGA	Genotyping *ceh-6(syb972)*
**EB110R**	GGCTGCCTCCATCTCGTTCT	Genotyping *ceh-6(syb972)*
**EB5F**	TCCAGTCTCTTCAGGTCAGTGATCT	Genotyping *egl-27(ok1670)*
**EB5R**	CGAGATTTCCAAATTCTTACCCGACTG	Genotyping *egl-27(ok1670)*
**EB6R**	GTGTAATTGACAGCGATGATGATGAAGG	Genotyping *egl-27(ok1670)*
**LIN5 FW 01**	GACAAGACCAAGTTATCGGC	Genotyping *lin-5(ev571),* digest w/ BglII
**LIN5 RV 01**	CCCATTGACTGAAATTCTTCG	Genotyping *lin-5(ev571),* digest w/ BglII
**mcm124F**	GAACTACAACTACTTTGGTCAACCATTGGGCCCTGCCACGTTTCC CCCAT	Genotyping *egl-5(n945),* digest w/ NcoI
**mcm124R**	CGTAAGATAGCATATAGGGTCAGACG	Genotyping *egl-5(n945),* digest w/ NcoI
**mcm125F**	CCGCGCCATTGACACCGATTTGGTAC	Genotyping *sem-4(n1971),* digest w/ Acc65I
**mcm125R**	CCTAACAAGCTAGCCTTTTCAGTTACAAAAACATCTCTCTTAACTG GGTA	Genotyping *sem-4(n1971),* digest w/ Acc65I
**oCG347 rev début GFP**	CCACTGACAGAAAATTTGTGCCC	Genotyping *sem-4 (syb1287)* and sequencing
**oCG368 sens PEST**	CTTAGCCATGGCTTCCCGCCGGCGGTGGCGGCGCAGGATGATG GCACGCTGCCCATGTCTTGTGCCCAGGAGAGCGGGATGGACCGT CACCCTGCAGCCTGTGCTTCTGCTAGGATCAAT	PEST sequence fw
**oCG369 rev PEST**	ATTGATCCTAGCAGAAGCACAGGCTGCAGGGTGACGGTCCATCC CGCTCTCCTGGGCACAAGACATGGGCAGCGTGCCATCATCCTGC GCCGCCACCGCCGGCGGGAAGCCATGGCTAAG	PEST sequence rv
**oCG370 sens MW PEST**	GGATTACACATGGCATGGATGAACTATACAAACTTAGCCATGGCT TCCCGCCGGCGGTGGC	Cloning *pest* sequence into pML801
**oCG371 rev MW PEST**	GGTAGCGACCGGCGCTCAGTTGGAATTCTACGAATGCTACACATT GATCCTAGCAGAAGCACAGGCTG	Cloning *pest* sequence into pML801
**oCG381 lin-26p f**	CAACTTGGAAATGAAATAAGCTTGCATGGATCCGACGTCTTCCCA TTGTCTTCCATTCATCTT	Cloning of the lin-26 rectal specific promoter into pPD97.82
**oCG382 lin-26p r**	GCTGAAAAGTGTCTAGAGTCGACCAAGGCCTGCAGCTGAAAATAA TCAATTAAAAATTTTAAAAAAAGTAAGCGAGGG	Cloning of the lin-26 rectal specific promoter into pPD97.82
**oCG390 for NLS1 kpnI**	AGGGTACCGAGCTCAGAAAAAATGACAGC	Cloning *2nls* into pPD95.75
**oCG391 rev GFP XhoI**	GGGTATCTCGAGAAGCATTGAACACCATAACAGAAAG	Cloning *2nls* into pPD95.75
**oCG411 sens egr-1 ku285**	GCCCCAAAAGCCTGAAAAAGCCCAAAATTTCTCAATTTCCA	Genotyping *egr-1(ku285),* digest w/ Hpy188III
**oCG412 rev egr-1 ku285**	GACGTCTCCGCAGAAGCTTCGGTGGC	Genotyping *egr-1(ku285),* digest w/ Hpy188III
**oCG444 lim-6 3int sens**	GGATACGCTAACAACTTGGAAATGAAATAGGCGCCCTTCTTGAGA TTGCG	Cloning *lim-6 intron4* into pPD95.75
**oCG445 lim-6 3int rev**	CGACCTGCAGGCATGCAAGCTAAAGATTGACATATTGGAGACATC TGCC	Cloning *lim-6 intron4* into pPD95.75
**oCG461 sens sox-2 CRISPR**	GGTTGTCTTTTGCAGTGTCCGG	Genotyping *sox-2(syb737)*

**oCG462 rev sox-2 CRISPR**	CAGAGCCATTTTCTTCCGCTGTC	Genotyping *sox-2(syb737)*
**oCG463 sens sem-4 CRISPR**	GACGACGAATCTTCGATGTGGC	Genotyping *sem-4 (syb1287)*
**oCG464 rev sem-4 CRISPR**	GGGGGAAAGAGGGAAAATTAGCTG	Genotyping *sem-4 (syb1287)*
**oCG556 sens HMG**	GACAGCCCAGATCTGGGTACCCAAGGAGGTGGAAAAGCGAAGA	cloning of POP-1 HMG
**oCG557 rev HMG**	GACGGAGCTCGAATTCGGATCCTTAACTCTTATCCCTTCGTTTCTTCG	cloning of POP-1 HMG
**oCR017 ceh-6 fw**	GGCGGATGCAAGATTTTACG	Genotyping *ceh-6(gk665)*
**oCR018 ceh-6 rv wt**	GGATGACGACGAAGGTATGAG	Genotyping *ceh-6(gk665)*
**oCR019 ceh-6 rev gk665**	CTGTGACAATGTTCCCGGAG	Genotyping *ceh-6(gk665)*
**oCR029 fw ceh-18(mg57)**	CCCACACCAGTTTCCACAAATGGC	Genotyping *ceh-18(mg57)*
**oCR030 rv ceh-18(mg57)**	AGGCTAGAAAGTTCTACGGG	Genotyping *ceh-18(mg57)*
**oCR036 rv ceh-18 wt**	GCTCGCCGCCTCAATTCTTGAT	Genotyping *ceh-18(mg57)*
**oCR061 fw AfeI EpiDeg**	GAGGGTACCAGAGCTCAAGCGCTATTCACCTGGCACCGACTAC	Cloning AfeI and XhoI sites into pOD1988
**oCR062 rv XhoI EpiDeg**	CCAGACTCCACCAGTTGGACTTGATCCATCTCGAGTTATCTGGAACAAAATGTAAG	Cloning AfeI and XhoI sites into pOD1988
**oCR073 AscI nanob**	ATAAAAGGCGCGCCAAAAAATGGATCAAGTCCAACTGGT	Cloning *nanobodyGFP::zif-1* into pSJ671
**oCR074 U54 ApaI**	GTAATAGGGCCCTTAACCCTCACTAAAGGGAACAAAA	Cloning *nanobodyGFP::zif-1* into pSJ671
**oCR075 tm1422 fw**	GGGCCAGAAGATTGCACCAC	Genotyping *vang-1(tm1422)*
**oCR076 tm1422 rv wt**	GCATGCTGAAGCCGAAACGT	Genotyping *vang-1(tm1422)*
**oCR077 tm1422 rv**	CGCAATCGGTAGAATTGAAAATTTCGG	Genotyping *vang-1(tm1422)*
**oCR087 nr2073 fw**	GTAATGCGCGAAGCTTCCTG	Genotyping *lim-6(nr2073)*
**oCR088 nr2073rv wt**	GGGAGCCTATAGGTCAGCTCT	Genotyping *lim-6(nr2073)*
**oCR089 nr2073 rv**	CCTCCGCTTGGAAGGACAAAA	Genotyping *lim-6(nr2073)*
**oCR092 rh308 fw**	GTGATAATGCTCGTATTGTCTATTCCATTGATTCCTAT	Genotyping *fmi-1(rh308)*, digest w/ AseI
**oCR093 rh308 rv**	GTGGATGAGATCCGCCGTCAG	Genotyping *fmi-1(rh308)*, digest w/ AseI
**oCR094bis**	CCTCTTAAAAACTTACCTCTCAAATTTGAACTTATTCAAGC	Genotyping *egl-20(n585)*, digest w/ HindIII
**oCR095 n585 rv**	GAACATTGGCATTTGTGGGTTCAAAC	Genotyping *egl-20(n585)*, digest w/ HindIII
**oCR096 n1792 fw**	CTTCAAAACTGTGCGAATCGTTTGAGATTTCAGCCCT	Genotyping *lin-44(n1792)*, digest w/ AvrII
**oCR097 n1792 rv**	CCTTTTGACCCTACCCGCCGAAC	Genotyping *lin-44(n1792)*, digest w/ AvrII
**oCR105 zu310 fw**	CCCACATTCATCCATCGATCTTTCATAAT	Genotyping *par-1(zu310)*, digest w/ SspI
**oCR106 zu310 rv**	GTCTCTGCTGTTCAATATTTGCATTCG	Genotyping *par-1(zu310)*, digest w/ SspI
**oCR107 ok2126fw wt**	CTGAACTGCCTGCTGCCAGA	Genotyping *gpr-1(ok2126)*
**oCR108 ok2126rv**	CACGAAAGTCATCAACGTATGTAGTAAAG	Genotyping *gpr-1(ok2126)*
**oCR109 ok2126fw mu**	CCAAGGCTCGACGGTTTGC	Genotyping *gpr-1(ok2126)*
**oCR113 ga80 fw**	GCATAGTGAGTTCTGGAATTGCTCGAACTGTGTTATACTGCCC	Genotyping *bar-1(ga80)*, digest w/ BclI
**oCR114 ga80 rv**	CATCCATGGCCGACTATGAGCCGATCCCCACTCTTTCTGAT	Genotyping *bar-1(ga80)*, digest w/ BclI

**oCR122 q645 fw**	CGATGGATTTCGACCGGCACC	Genotyping *pop-1(q645),* digest w/ ClaI
**oCR123 q645 rv**	GATATAAAAATACACAAAAATGATGGCCGACGAAGAGCTCATCGA	Genotyping *pop-1(q645),* digest w/ ClaI
**oCR128 n1378 fw**	CAACACCGAATCCAAAAAACGAAAATCCACTGCTTGGCATG	Genotyping *sem-4(n1378),* digest w/ SphI
**oCR129 n1378 rv**	CCACGAGTTGTGAATGCGCGTCCAC	Genotyping *sem-4(n1378),* digest w/ SphI
**oCR138 syb971fw**	GACATTCGAAGCTCTGATGATG	Genotyping *lim-6(syb971)*
**oCR139 syb971rv wt**	GTGCAAAGATTAGAGCTCTGAC	Genotyping *lim-6(syb971)*
**oCR140 syb971rv mu**	GGGTATCTCGAGAAGCATTG	Genotyping *lim-6(syb971)*
**oCR144 n1051 fw**	CACTACAGAGTTATGGCAAACATCGACTACCTCTCGTTCCCAT	Genotyping *lin-18(n1051),* digest w/ NcoI
**oCR145 n1051 rv**	CCTGTCGCAATTTCACTTTCAACGGCTC	Genotyping *lin-18(n1051),* digest w/ NcoI
**oCR149 gm122 fw**	GACCACGATTTACTTCGGCAACG	Genotying *cam-1(gm122),* digest w/ BclI
**oCR150 gm122 rv**	CATCATATGTATAAAGTTTGCGAATCGGATTCTAATGAT	Genotying *cam-1(gm122),* digest w/ BclI
**oCR151 q544 fw**	CCTGTTGGCGGAGGAGGTTGATCATGTGG	Genotyping *sys-1(q544),*digest w/ AflIII
**oCR152 q544 rv**	GGCAAAAAGATCCTCACATGAAACACTGCGCAAATCACGT	Genotyping *sys-1(q544),*digest w/ AflIII
**oCR153 n671**	CCGCATTTTTCGTAGATCACACC	Sequencing *lin-17(n671)*
**oCR154 n671**	CGAGCACATTCCACAGAAGATG	Sequencing *lin-17(n671)*
**oCR155 lin-17p fw**	CTGAAGCTTACACTTTGTTCGCTC	Cloning *lin-17p* reporter
**oCR156 lin-17p rv**	CGGCTGCAGTTTGGAGAAGGAGCCAGTCTCTC	Cloning *lin-17p* reporter
**oCR157 wrm-1 fw**	GATGTTCTTCCGACTGAATGC	Sequencing and genotyping *wrm-1(ne1982ts)*
**oCR158 wrm-1 rv**	CTTGTGCTCCACCCATTTG	Sequencing and genotyping *wrm-1(ne1982ts)*
**pLG7F**	ACGCGTCGACGTGAAAACATAGTGTTTCCCAGTAC	*sox-2* antisens construct
**pLG7R**	GCTCTAGAGATATTATACATATTTCATAAAGCCAAC	*sox-2* antisens construct
**oSKS-233**	ATGAACTATACAAAGCTTCGAATTCTGCAGTCGACA TGTCTTCTGCTCGTCGTTGC	To amplify cki-1 for construction of pSJ1108
**oSKS-234**	ATTCATGCATAGGCCTGCGGCCGCGCTAGCCTAGTAT GGAGAGCATGAAGATCGAGTTC	To amplify cki-1 for construction of pSJ1108
**oSKS-235**	ATGGACTACAAGGACGACGATGACAAGTAAGGATCC GAATTCGAGCTCC	To insert Flag at the C-terminal of hmg-pop1 in pSJ769, for construction of pSJ1107
**oSKS-236**	ACTCTTATCCCTTCGTTTCTTCGTC	To insert Flag at the C-terminal of hmg-pop1 in pSJ769, for construction of pSJ1107
**oSKS-237**	AACCAAAGTTCTCACTGTCAGAACCA	To delete intron from cki-1 for construction of pSJ1112
**oSKS-238**	CTGTAGAACTCCGGAACACAATTCTCT	To delete intron from cki-1 for construction of pSJ1112
**psj6094sox-2 F**	TCGACATGATGATGGATCCGGATTCAGC	*6XHis::sox-2* construct
**psj6094sox-2 R**	GTGCGGCCGCAAGCTTGGTACC	*6XHis::sox-2* construct
