# Supplementary material for: A natural transdifferentiation event involving mitosis is empowered by integrating signaling inputs with conserved plasticity factors
Source: Cell Rep. 2022 Sep 20;40(12):111365. doi: 10.1016/j.celrep.2022.111365 (PMC9513805; doi:10.1016/j.celrep.2022.111365)
Supplement: Document S2. Article plus supplemental information [file mmc2.pdf]

Cell Reports

Article

A natural transdifferentiation event involving mitosis is empowered by integrating signaling inputs with conserved plasticity factors

Graphical abstract

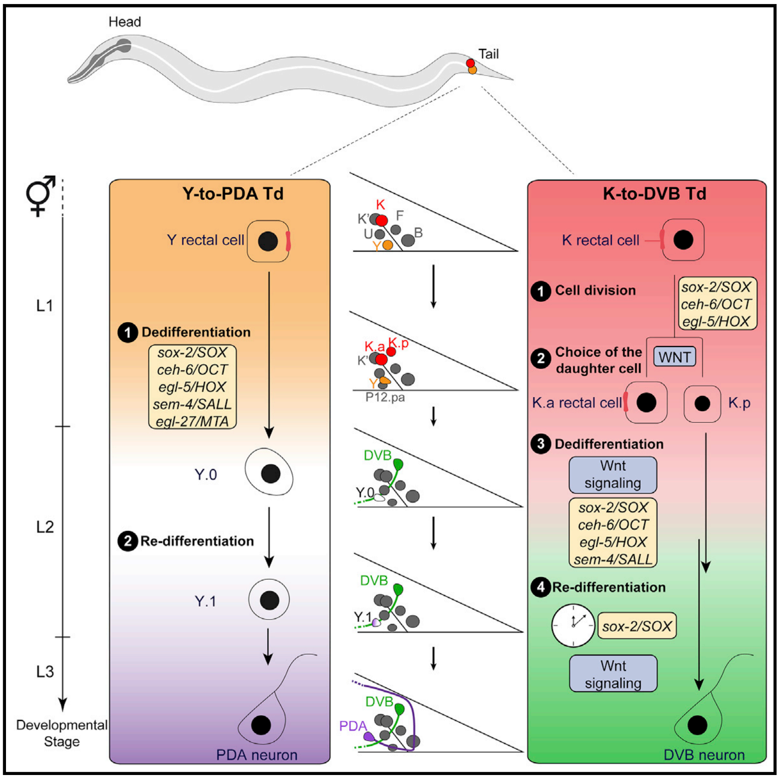

Authors

Claudia Riva, Martina Hajduskova, Christelle Gally, Shashi Kumar Suman, Arnaud Ahier, Sophie Jarriault

Correspondence

gally@igbmc.fr (C.G.),
sophie@igbmc.fr (S.J.)

In brief

Using single-cell natural transdifferentiation models in *C. elegans*, Riva et al. demonstrate that conserved and event-specific mechanisms allow transdifferentiation with or without cell division. Although evolutionary-conserved plasticity factors are required independently of cell division, Wnt signaling activity is context dependent. Dichotomic interactions between SOX-2 and Wnt may control re-differentiation timing.

Highlights

- Transdifferentiation occurs naturally with or without cell division in *C. elegans*
- Wnt selects the daughter cell which transdifferentiates in presence of division
- Conserved plasticity factors and Wnt act in parallel to erase the initial identity
- Changes in stoichiometry of SOX-2 and TCF could control timing of re-differentiation

Riva et al., 2022, Cell Reports *40*, 111365
September 20, 2022 © 2022 The Authors.
https://doi.org/10.1016/j.celrep.2022.111365

Cell Reports

CellPress
OPEN ACCESS

Article

A natural transdifferentiation event involving mitosis is empowered by integrating signaling inputs with conserved plasticity factors

Claudia Riva,[1,2,6] Martina Hajduskova,[1,3,4,6] Christelle Gally,[1,6,*] Shashi Kumar Suman,[1] Arnaud Ahier,[1,5] and Sophie Jarriault[1,7,*]
[1]Development and Stem Cells Department, IGBMC, CNRS UMR 7104, Inserm U 1258, Université de Strasbourg, 67400 Illkirch, France
[2]Present address: Department of Biomedicine, University of Basel, Mattenstrasse 28, 4058 Basel, Switzerland
[3]Present address: Charles University, Faculty of Medicine in Pilsen, Husova 3, 301 00 Plzen (Pilsen), Czech Republic
[4]Present address: Genetika Plzen, s.r.o., Parkova 11a, 32600 Plzen, Czech Republic
[5]Present address: Queensland Brain Institute, QBI Building 79, University of Queensland, St. Lucia, QLD 4072, Australia
[6]These authors contributed equally
[7]Lead contact
*Correspondence: gally@igbmc.fr (C.G.), sophie@igbmc.fr (S.J.)
https://doi.org/10.1016/j.celrep.2022.111365

SUMMARY

Transdifferentiation, or direct cell reprogramming, is the conversion of one fully differentiated cell type into another. Whether core mechanisms are shared between natural transdifferentiation events when occurring with or without cell division is unclear. We have previously characterized the Y-to-PDA natural transdifferentiation in *Caenorhabditis elegans*, which occurs without cell division and requires orthologs of vertebrate reprogramming factors. Here, we identify a rectal-to-GABAergic transdifferentiation and show that cell division is required but not sufficient for conversion. We find shared mechanisms, including erasure of the initial identity, which requires the conserved reprogramming factors SEM-4/SALL, SOX-2, CEH-6/OCT, and EGL-5/HOX. We also find three additional and parallel roles of the Wnt signaling pathway: selection of a specific daughter, removal of the initial identity, and imposition of the precise final subtype identity. Our results support a model in which levels and antagonistic activities of SOX-2 and Wnt signaling provide a timer for the acquisition of final identity.

INTRODUCTION

Until the beginning of the last century, the common belief was that the identity of differentiated cells is irreversible under physiological conditions (Merrell and Stanger, 2016), even though cellular reprogramming phenomena were observed starting in the 18th century (Trembley, 1744; Virchow, 1886). Since the 1950s, reprogramming can also be artificially induced *in vitro* and *in vivo* through somatic cell nuclear transfer, cell fusion, or using cocktails of transcription factors (TFs), noncoding RNAs (ncRNAs), and small molecules in cells from different organisms (reviewed by Rothman and Jarriault, 2019; Takahashi and Yamanaka, 2016; Xu et al., 2015).

Various types of reprogramming have been described in nature, during development, regeneration, or disease or following experimental induction (Brockes and Kumar, 2002; Thorel et al., 2010; Xu et al., 2015; Yanger et al., 2013). Differentiated cells can retro-differentiate, for instance into pluripotent stem cells (PSCs) (Takahashi and Yamanaka, 2006), or convert "directly" to another differentiated identity, also known as transdifferentiation (Td) or direct cell reprogramming (Eguchi and Kodama, 1993). The term "direct" does not refer to a potential lack of intermediate steps during the Td process: unstable, transient cellular states, with either a mixed identity or another identity, can exist (Lambert et al., 2021). Okada defined precise criteria to identify Td events: (1) the phenotypes of the initial and final cell identities are clearly defined and distinct, and (2) a lineal relationship between the two cells is established (Eguchi and Kodama, 1993; Okada, 1986, 1991). Although experimentally induced Td is often inefficient, natural Td is robust (Eguchi et al., 2011) and offers the opportunity to decipher the cellular and molecular mechanisms at play in a complex tissue during reprogramming.

Do different Td events share the same cellular steps and common mechanistic principles? For instance, the importance of cell division during reprogramming has not been fully addressed. Some Td events occur in the absence of cell division (Jarriault et al., 2008) or with a facultative cell division (e.g. Di Tullio and Graf, 2012), but both natural and induced Td often involve mitosis (Lambert et al., 2021). Most studies on the role of cell division were conducted inducing reprogramming under artificial conditions: conversion of B cells or fibroblasts into PSCs calls on multiple divisions (Hanna et al., 2009), and a rapid cell cycle is a key feature of efficient reprogramming to pluripotency (Guo et al., 2014); on

This is an open access article under the CC BY license (http://creativecommons.org/licenses/by/4.0/).

the other hand, Td of fibroblasts to neurons or of pre-B cells to macrophages does not require cell division (Di Tullio and Graf, 2012; Fishman et al., 2015; Marro et al., 2011). What the contribution of cell division is during natural Td remains an open question, and the extent of mechanistic variations among different Td events, even within a given organism, is unclear.

Our lab has been taking advantage of *C. elegans* to study natural Td *in vivo* at single-cell resolution. We previously characterized a natural Td event occurring in 100% of the animals during larval development, in which the Y rectal cell becomes the motoneuron PDA without cell division or cell fusion (Jarriault et al., 2008). Y-to-PDA Td occurs stepwise with the erasure of the initial identity, before re-differentiation (Richard et al., 2011). Through unbiased electrophoresis mobility shift (EMS) and RNAi screens, we identified genes that control the different steps of the process (Kagias et al., 2012; Jarriault et al., 2008; Richard et al., 2011; Zuryn et al., 2010, 2014) and are orthologs of factors known to have reprogramming activities or to be associated with pluripotency in mammalian cells (*ceh-6/Oct*, *sox-2/SOX*, *sem-4/SALL*, and *egl-27/MTA*; Julian et al., 2017; Malik et al., 2018; Ng and Surani, 2011). This led us to ask whether these factors could constitute a conserved plasticity cassette shared with other Td events.

In this study, we addressed the role of cell division and the involvement of core reprogramming factors by characterizing another putative Td event in the worm rectum. We reasoned that comparing two Td events in the same body region and tissue would allow us to outline event-specific modalities. We describe the Td of the K rectal cell, which divides and gives rise to the anterior daughter K.a, remaining in the rectum, and to the posterior daughter K.p that becomes the DVB GABAergic neuron later in the L2 stage. We characterized K, K.a, K.p, and DVB identities and confirmed that K-to-DVB is a bona fide Td event. We showed that K division is oriented, asymmetric, and crucial for DVB formation and that the *C. elegans* Wnt/β-catenin asymmetry pathway ensures the Td of the K.p daughter, formed at a stereotyped posterior position. Furthermore, we found that the plasticity factors required for Y-to-PDA (Kagias et al., 2012) are also required for K-to-DVB for after K division. By dissecting the relationships between plasticity factors and the Wnt signaling pathway, we found that Wnt most likely acts in parallel to SEM-4, to erase the initial identity of K.p, and in antagonism with SOX-2, providing a developmental timer to refine the re-differentiation step through direct regulation of the expression of the DVB terminal selector *lim-6*. Our study provides an integrated view of how a fully differentiated cell is naturally reprogrammed into a different cell type: core plasticity factors are required for Td independently of the presence of cell division, and parallel context-dependent signaling pathways regulate Td dynamics and the final identity.

RESULTS

K-to-DVB involves a differentiated rectal cell that gives rise to a neuron

Through the analysis of the embryonic and post-embryonic somatic cell lineage in *C. elegans* (Sulston and Horvitz, 1977; Sulston et al., 1983), we pinpointed putative cell fate changes occurring during larval development together with the already known Y-to-PDA Td (Jarriault et al., 2008). Here we focus on the K rectal cell, which gives rise to two daughter cells through a single cell division in late L1 stage (Figure 1A): K.a, which replaces K in the rectum, and K.p, which subsequently becomes a GABAergic motoneuron called DVB (McIntire et al., 1993).

K is born in the embryo and forms one of the three rectal rings with its sister K′ through adherens junctions (Sulston et al., 1983). The six *C. elegans* cells forming the rectum are differentiated, specialized epithelial cells (Altun and Hall, 2009). K rectal identity is confirmed at the ultra-structural level in early L1 larvae, where it shows a typical rectal-epithelial morphology resembling that of K′ (Figure 1B). Moreover, K expresses several epithelial and rectal markers, while lacking any neuronal markers (Figure 1C; Table S1) (Ferreira et al., 1999; Jarriault et al., 2008; Labouesse et al., 1996). Thus, K is fully differentiated on the basis of differential interference contrast (DIC) appearance, markers expression, electron microscopy (EM) morphology, and function. On the contrary, DVB lacks epithelial and rectal markers, expresses neuronal genes, and develops in a typical neuronal morphology (White et al., 1986) to fulfill its GABAergic motoneuron function in defecation (Figure 1C; Table S1).

Altogether, these observations demonstrate that K and DVB display different terminal identity features, the former being a fully differentiated rectal cell and the latter a GABAergic neuron. The development of a neuron from a fully differentiated rectal cell is intriguing and reminiscent of the Y-to-PDA Td and, per our characterization of the initial and final identities, is in agreement with the original definition of Td (Okada, 1986).

K division, which is oriented and asymmetric, is necessary for DVB formation, but K.p is not yet neuronal at birth

We first investigated the role of cell division in K-to-DVB, by assessing whether K division *per se* is necessary for the formation of DVB. In *lin-5(ev571 ts)/NuMA* mutants, in which K cytokinesis is blocked in 90.5% of the animals at 25°C (Figures 2A and 2B) (Izumi et al., 2006), DVB never formed when K had not divided (Figure 2B). As already observed in other *C. elegans* cells (Lorson et al., 2000), DNA replication occurred in K in most of the worms in which K cytokinesis failed, suggesting that this process is not enough for DVB formation (Figure S1A). Consistently, when blocking K in G0 cell cycle phase through the overexpression of the cell cycle inhibitor *cki-1* (van den Heuvel, 2005), DVB formation was prevented (Figures S1B and S1C). Altogether, these results show that K division is necessary for DVB differentiation.

We characterized K division with time-lapse spinning-disk microscopy, using *[col-34p::his-24::mcherry]* and *[hmr-1::gfp]* transgenes to visualize the rectal cells' nuclei and their apical junctions and *[unc-47p::gfp]* transgene to monitor DVB formation. We observed that K.p buds off from the K cell posteriorly, above the rectal cell F, without disrupting K apicobasal polarity (Figure 2C) or rounding of the K cell, as is often associated with cell division (Cadart et al., 2014), or loss of adherence of K to K′ cells. This division mode likely allows the maintenance of the integrity of the rectum during cytokinesis. As K division appears to occur with an anteroposterior orientation, we analyzed this parameter in a quantitative manner. Because K division is very fast, we estimated its orientation by measuring the angle formed by the rectal slit with the K.a and K.p's nuclei alignment, a maximum of 1 h after

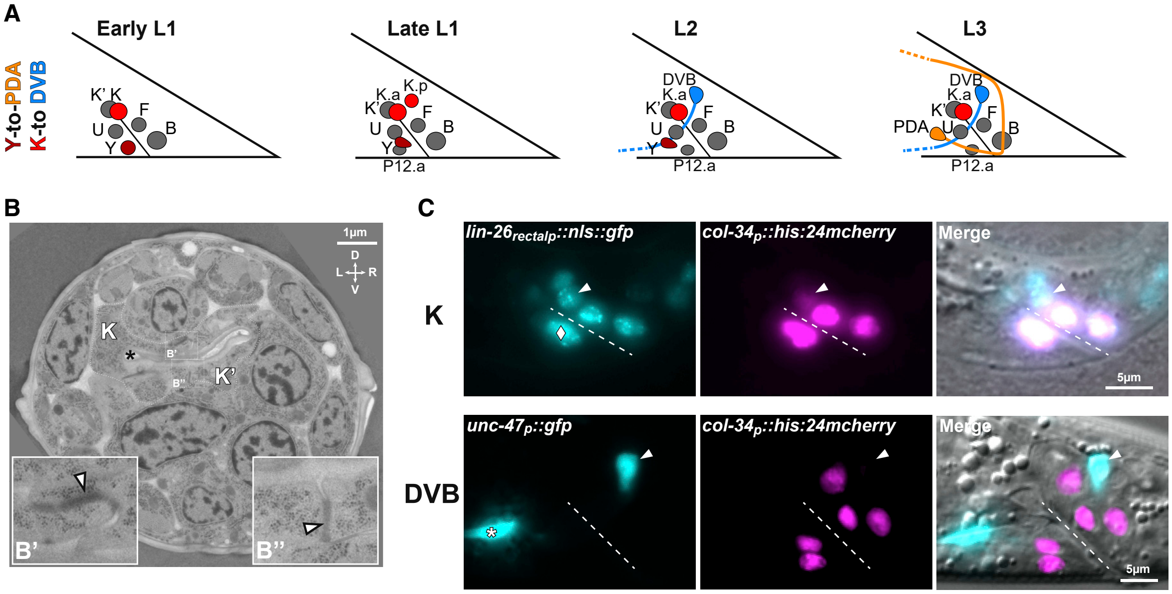

Figure 1. K-to-DVB is a transdifferentiation model

(A) Illustration of the rectum during the formation of PDA and DVB neurons from the Y (dark red) and K (red) rectal cells respectively in hermaphrodites. In early L1, both Y and K are part of the rectum, a tube made of three bi-cellular rings (Y-B, U-F, and K-K′). In late L1, K divides into two daughter cells, K.a and K.p. In L2, Y starts to retract from the rectum, while P12.pa replaces it in the rectum; K.a is part of the rectum in place of its mother and K.p becomes DVB. In L3, PDA is formed. Rectal cell nuclei are represented.

(B) Electron micrograph of the rectum of a newly hatched L1 hermaphrodite showing the K and K′ cells outlined with a dashed white line. Black star, rectal lumen; white arrowheads, electron-dense apical junctions. (B′ and B″) Magnification of the boxed areas in B, illustrating electron-dense apical junctions between K and K′; in B′ the membrane of an upper cell is visible. D, dorsal orientation; V, ventral orientation; L, left orientation; R, right orientation.

(C) Fluorescent and DIC images of nuclear *lin-26p::gfp* in the rectum of an L1 animal (top) and *unc-47p::gfp* in GABAergic neurons in an L3 animal (bottom). *col-34p::his-24::mcherry* allows the visualization of the rectal cells nuclei and K.p after its birth. White rhombus in L1, migrating Y rectal cell partially overlapping U; white star in L3, VD13 GABAergic neuron; arrowheads, K in L1 and DVB in L3; dashed line, rectal slit. Anterior is left and ventral is bottom.

division. Our results show that orientation of K division is stereotyped among animals, forming an angle of 51.2° ± 7.6° (Figure 2D). As the orientation implied, only the anterior daughter K.a inherits the apical proteins HMR-1 (Figure 2C), AJM-1, and DLG-1 (Figures 2F and 2G). Additionally, quantification of the nuclear volumes of K.a and K.p 1 h after K division, using the *gaIs245*[*col-34p::his-24::mcherry*] chromatin marker as an approximation, revealed an asymmetry also in their nuclear volumes (Figure 2E). Thus, K division appears oriented and asymmetric.

These results prompted us to investigate whether a neuronal daughter is directly produced by cell division. We determined the timeline of the cellular events occurring during K-to-DVB: K divides around 11.5 h post-hatching (PH) at 20°C; around 4 h later, after the L1-to-L2 molt, *unc-47* expression is detected in the K.p daughter in a few young L2 larvae, and 16–17 h PH, *unc-47p::gfp* is expressed in all the L2s scored (Figures S2A and S2B). Conversely, K, K.a, and newly born K.p never express *unc-47p::gfp.* As *unc-47/SLC32A1* is involved in GABA transport and necessary for GABAergic neuronal activity (McIntire et al., 1993), we will henceforth use “K.p” when considering K posterior daughter until 16 h PH and “DVB” after this time point. These observations suggest that K.p is not a differentiated GABAergic neuron at birth. In agreement with this, although apical regions are not inherited, the basolateral epithelial marker LET-413 is present in K.p as in K and K.a in all observed animals, even when a destabilized version of LET-413:GFP is used, suggesting active expression of *let-413* gene (Figure 2H; Table S1). The proportion of animals with LET-413:GFP in K.p decreases to less than 20% after 16 h PH, when the DVB marker *unc-47* is expressed in all the worms (Figure S2B). On the same line, the epithelial TF *lin-26* promoter is active in K.p after K division (Figure S2B) and quickly turned off, as *gfp* reporter expression is absent in about 20% of the worms 1 h after K division. *lin-26* expression in K.p is confirmed by single-molecule fluorescence *in situ* hybridization (smFISH) (Ji and van Oudenaarden, 2012) experiments to detect *lin-26* mRNA (Figures S2C and S2D) and by previous antibody staining data (Labouesse et al., 1996). Finally, we observed the presence of rectal markers such as *egl-5/HOX*, *col-34*, and *got-1.2/GOT1* in K.p (Table S1), while both pan-neuronal (e.g., *unc-33/DPYS*, *unc-119/UNC119*) and GABAergic terminal differentiation genes (e.g., *unc-25/GAD*) appear later (Table S1).

These data show that K division is oriented, asymmetric, and required to form DVB. Despite the differences between K.a and K.p at birth, K.p retains important epithelial and rectal features, inheriting and expressing basolateral, rectal, and hypodermal factors. Successive steps are required to convert K.p into DVB.

The Wnt/β-catenin asymmetry pathway is required for K-to-DVB

The stereotyped and oriented nature of K division prompted us to investigate the mechanisms regulating it and their impact on

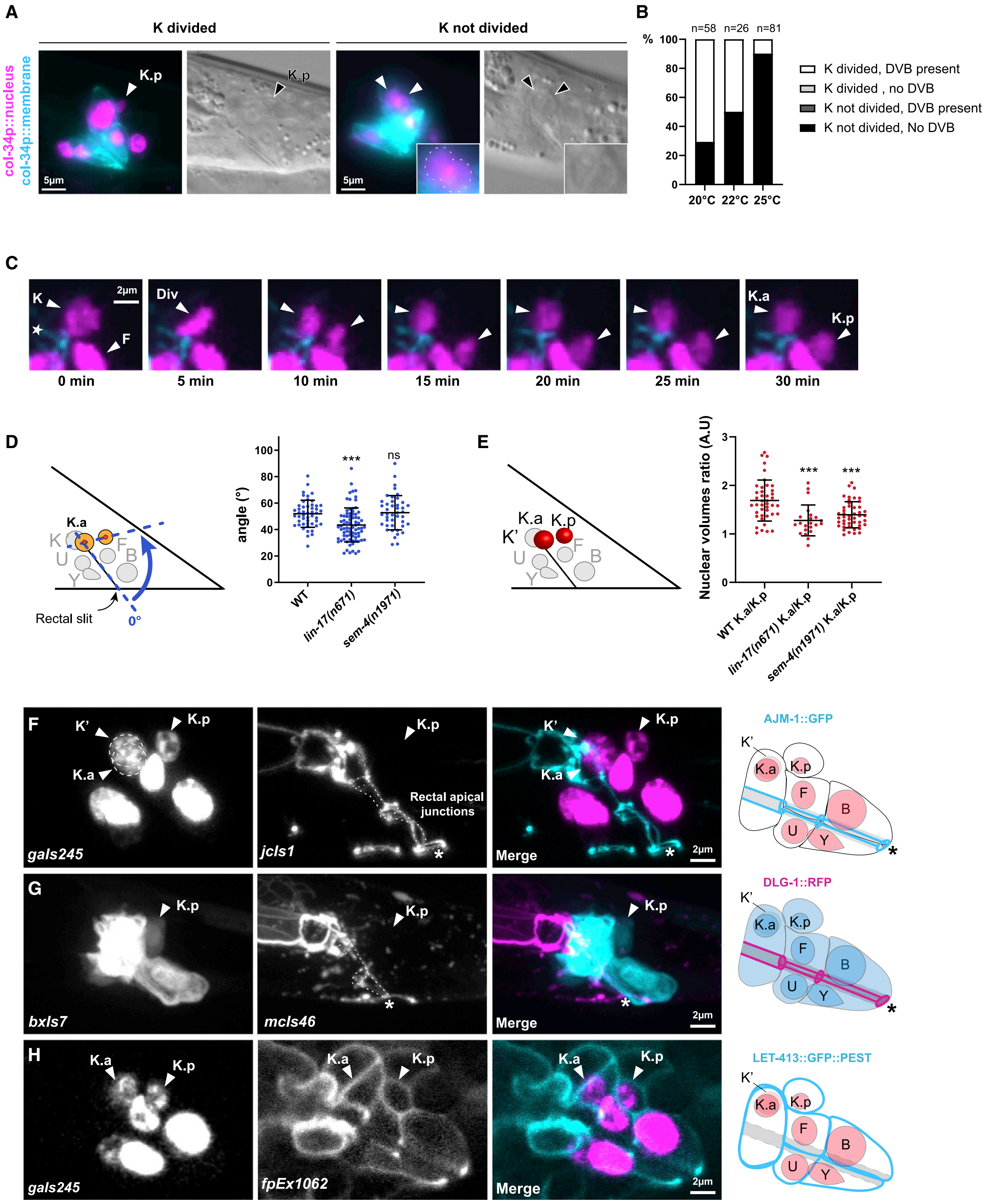

(legend on next page)

K-to-DVB. We focused on the Wnt signaling pathway, known to orient the mitotic spindle (Schlesinger et al., 1999; Goldstein et al., 2006; Heppert et al., 2018) and regulate several asymmetric cell divisions in *C. elegans* (Mizumoto and Sawa, 2007; Sawa and Korswagen, 2013). To test its potential role during K-to-DVB, we screened mutants of the Wnt pathway for DVB formation. The strong loss of function *lin-17(n671)* (Sawa et al., 1996) (encoding one of the four Frizzled receptors in *C. elegans* and expressed in rectal cells [Sawa and Korswagen, 2013]) led to >90% "no DVB" worms (Figure 3A). We next analyzed the requirement of several other Wnt pathway components: *lin-44* and *egl-20*, which encode WNT ligands expressed in the rectal area (Harterink et al., 2011); *pop*-1 which encodes the unique T cell factor (TCF)/lymphoid enhancer factor (LEF) in *C. elegans* (Lin et al., 1995); and *bar-1*, *sys-1*, and *wrm-1*, which encode the three worm β-catenins (Sawa and Korswagen, 2013). We found that *pop-1/TCF*, *lin-44/WNT*, *sys-1*/β-catenin, and *wrm-1*/β-catenin are required for K-to-DVB to various degrees, while *egl-20/WNT* and *bar-1*/β-catenin are dispensable (Figure 3A). Thus, components of the Wnt/β-catenin asymmetry pathway (Mizumoto and Sawa, 2007) are required for DVB formation.

We next examined if K division was altered in worms with defective Wnt signaling. In *lin-17* mutant, the highly penetrant absence of DVB is not due to a failure in K division. However, the orientation of K division is affected in 8.9% of the animals (Figure 2D), with K.p positioned more dorsally or ventrally. Although not accounting for the total "no DVB" defects in *lin-17* mutants, these data suggest that K division is abnormal in a fraction of them. We tested the involvement of other Wnt-related pathways, such as the non-canonical Wnt pathway, known to act directly on spindle orientation through *ced-10/RAC1* (Cabello et al., 2010; Schlesinger et al., 1999), *lin-18/RYK*, *cam-1/ROR*, and the planar cell polarity (PCP) pathways (*vang-1/VANGL* and *fmi-1/CELSR2*) (Sawa and Korswagen, 2013); none of these genes is involved in K-to-DVB (Figures S3A and S3B).

To directly test whether the orientation of K division has an impact on DVB formation, we aimed at perturbing it using mutants known to randomize the mitotic spindle (Gotta and Ahringer, 2001). As all temperature conditions tested for the *lin-5(ev571 ts)* mutant resulted in either wild-type DVB or "no K cytokinesis/no DVB" (Figure 2B) and *gpr-1* and *gpa-16/GNAI1* mutants showed no defect (Figure S1D), we examined the division angle in *goa-1(sa734)* null mutant displaying "no DVB" defect in 0.9% of the animals (Figure S1D). Using a score-recover-score approach, we found that 8.6% of the animals exhibited an abnormal K division angle at late L1 stage (Figure S1E) that did not translate into any "no DVB" defects. Thus, although LIN-17/FZD activity may affect K division axis, altered orientation of K division in itself does not seem to affect DVB formation.

These results show that the canonical Wnt/β-catenin asymmetry pathway is involved in K-to-DVB, but the wild-type orientation of K division is not required.

Both selection of one K daughter and the K-to-DVB conversion require WNT activity

The Wnt/β-catenin asymmetry pathway controls the polarity of several cell divisions during *C. elegans* development through WNT ligands (Herman, 2002; Herman et al., 1995). To determine whether any WNT ligands polarize K division, we examined the phenotypes of *lin-44/WNT* single and *lin-44/WNT;egl-20/WNT* double mutants (Figures 3B and 3C). *lin-44* is expressed posteriorly to K in hypodermal cells, while *egl-20* is expressed in some rectal cells, including K, and in other cells in the rectal area (Harterink et al., 2011). DVB is present in most *lin-44* and *lin-44;egl-20* mutant animals (Figures 3B and 3C), contrary to what is observed in *lin-17* mutants, but appears to originate from K.a in 10.8% of *lin-44* mutant worms and in 57.9% of double mutant animals (Figure 3C): a reversed polarity of cell division phenotype as observed for T and the male B, F, and U cells (Herman et al., 1995). Consistently, LIN-17/FZD receptor transiently localizes at the posterior cortex around 2 h before cell division (not shown). By contrast, reversed polarity of cell division is never observed in Wnt pathway mutants downstream of WNTs such as *pop-1/TCF* and *lin-17/FZD* (Figure S3C). We conclude that the Wnt/β-catenin asymmetry pathway regulates both the polarity of K division and K.p conversion into DVB.

To dissect how this conversion occurs, we characterized K.p identity in *lin-17* mutant. K.p nucleus appears as big as K.a nucleus at all times in 77% of *lin-17(n671)* animals, reminiscent of

Figure 2. K division is oriented and asymmetric and gives rise to a K.p cell that retains epithelial features

(A) Fluorescent and DIC images of *col-34p::his-24::mcherry* (*col-34p::nucleus*) and *col-34p::ph::gfp* (*col-34p::membrane*) in *lin-5(ev571 ts)* mutants where K division has (left) or has not (right) occurred. Absence of K cytokinesis in the worm on the right is evidenced by the presence of a unique cytoplasmic membrane around two nuclei. See also Figure S1.

(B) Bar plot summarizing frequency of K division occurrence and absence of DVB in single *lin-5(ev571 ts)* mutants at different restrictive temperatures, through a score-recover-score strategy. n, total number of animals scored.

(C) Time-lapse imaging of K division using *col-34p::his-24::mcherry* and *hmr-1::GFP* to visualize rectal cell nuclei and apical junctions respectively. Time interval, 5 min. Div, metaphase of K division; arrowheads, K, K.a, and K.p nuclei; white star, apical junction. See also Figure S2.

(D and E) Quantification of the angle of K division with respect to the rectal slit (D) and quantification of the nuclear volumes of K.a and K.p using *col-34p::his-24::mcherry* to visualize the nuclei (E) in late L1 wild-type, *lin-17(n671)* and *sem-4(n1961)* animals. (D) Blue dashed lines (left), landmarks used for the measurements of the angles (curved arrow). Forty-nine, 79, and 43 animals were scored in wild-type, *lin-17*, and *sem-4* mutants, respectively. Mean and standard deviation between biological replicates of the percentage of worms scored are represented. *p < 0.05; **p < 0.01; ***p < 0.001; ****p < 0.0001, and ns, not significant.

(E) Dot plot representing the ratio for K.a/K.p in arbitrary units. Forty-two, 22, and 46 nuclei were measured in wild-type, *lin-17*, and *sem-4* mutants, respectively.

(F–H) Confocal images of late L1 wild-type larva, and corresponding schematics representing (F) the apical junction marker AJM-1::GFP (*jcIs1*) together with *gaIs245[col-34p::his-24::mcherry]* to visualize rectal cell nuclei and (G) the apical junction marker DLG-1::RFP (*mcIs46*) with *bxIs7[egl-5p::gfp]* to visualize rectal cells. For (F) and (G), apical junctions (dashed lines) are present between rectal cells and along the rectal lumen but not in K.p. White star, rectal opening. (H) Localization and expression of a destabilized basolateral marker LET-413::GFP::PEST (*fpEx1062*). Rectal cell nuclei are visualized with *gaIs245[col-34p::his-24::mcherry]*. The K.a, K.p, and sometimes K′ cells are indicated on the pictures. Anterior is left and ventral is bottom.

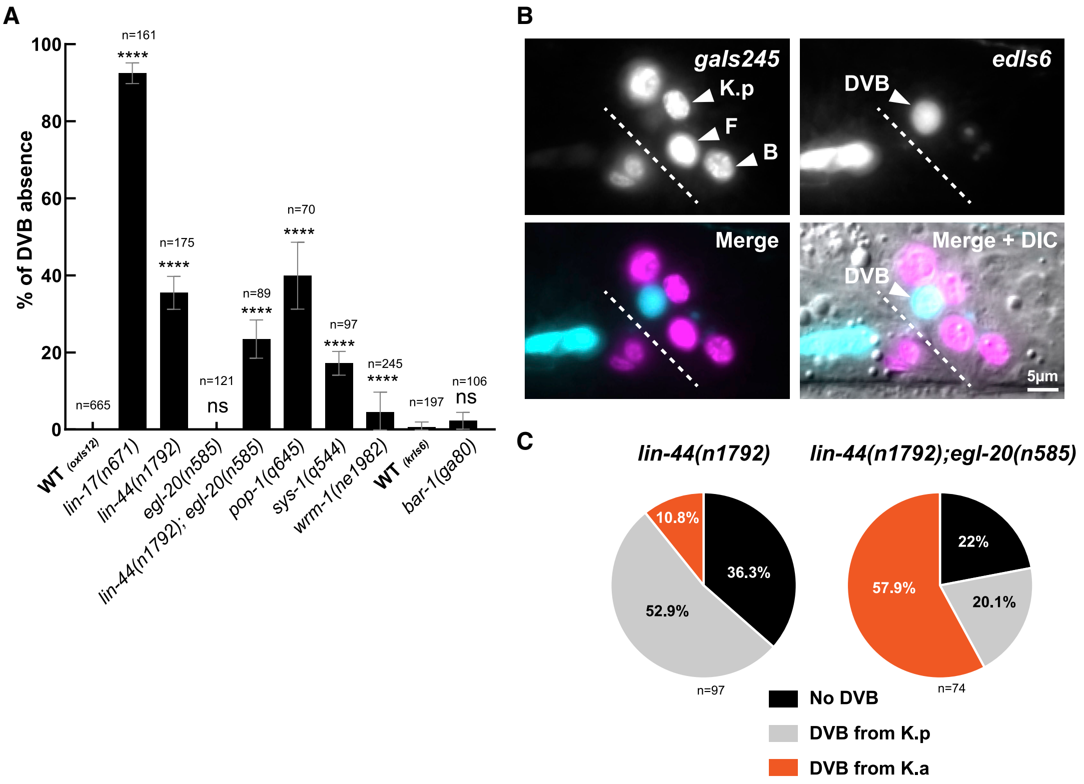

Figure 3. Wnt signaling is involved in DVB formation

(A) Bar plot showing the penetrance of "no DVB" defect in mutants of the Wnt pathway. *oxIs12* transgene was used for all except *bar-1* mutant, which is on the same chromosome. n, total L4 larvae scored. See also Figures S3 and S4. Mean and standard deviation between biological replicates of the percentage of worms scored are represented. *p < 0.05; **p < 0.01; ***p < 0.001; ****p < 0.0001, and ns, not significant.

(B) Fluorescence microscopy pictures of a double mutant *lin-44(n1792); egl-20(n585)* L4 worm in which DVB appears to be formed from K anterior daughter. *gaIs245* highlights the rectal cells' nuclei and *oxIs12* the GABAergic neurons including DVB. Anterior is left and ventral is bottom.

(C) Representation of the percentages of the indicated phenotypes in *lin-44(n1792)* single and *lin-44(n1792); egl-20(n585)* double mutants shown in (A). When present, DVB is formed mostly by K.a in the double mutant strain. n, total animals scored.

a hypodermal identity (Figure 2E). Moreover, all the examined epithelial and rectal markers persist in K.p, including *let-413*, *lin-26*, and *egl-5* (Figures S4B–S4D; Table S1), and even *ajm-1*, which is found in K.p although not inherited from K (Figures S4A and S5; Table S1). On the contrary pan-neuronal and GABAergic markers are never expressed (Figures S4E–S4I; Table S1). Thus, K.p remains rectal in the absence of *lin-17*.

In sum, the Wnt/β-catenin asymmetry pathway plays at least two distinct roles in K-to-DVB Td: (1) *lin-44/WNT* acts as a positional cue and determines which daughter cell of K will subsequently become DVB, and (2) Wnt signal allows epithelial K.p to convert into DVB.

Y-to-PDA plasticity factors are required for K-to-DVB

As our data suggest the requirement of mechanisms additional to K division, we investigated whether Y-to-PDA plasticity genes are also required for K-to-DVB. Supporting this possibility, traditional and CRISPR-KI reporters show that these factors are expressed in K (Table S1; Ferreira et al., 1999; Bürglin and Ruvkun, 2001; Jarriault et al., 2008; Vidal et al., 2015).

We found that *sem-4(n1971)* and *egl-5(n945)*, which are viable null mutants (Chisholm, 1991; Basson and Horvitz, 1996), display the strongest DVB defect with, respectively, 92% and 85% "no DVB" worms (Figure 4A), similar to the PDA defect (Jarriault et al., 2008). As absence of *ceh-6* and *sox-2* is lethal before the conversion occurs, we assessed their involvement using a rectal-specific mutant for *ceh-6(gk665)* (Ahier et al., 2020) and rectal-specific mild RNAi for *sox-2*. *ceh-6(gk665)* and *sox-2* knockdown led to significant defects in PDA and DVB formation (Figure 4A), with a low penetrance, probably because of experimental limitations. To rule out involvement of other family members, we tested their paralogs, but none displayed any significant "no DVB" defects (Figure S6). Consistently, it was previously shown that *sox-2* paralog *sox-3* is not required for DVB formation (Vidal et al., 2015). Finally, we observed that neither *egl-27* nor its paralog *lin-40* mutations has a strong impact on DVB formation as opposed to Y-to-PDA Td (Figures 4A and S6).

These results demonstrate that most genes required for Y-to-PDA are required for K-to-DVB.

Plasticity factors enable K division and erasure of K.p identity

As cell division is necessary for DVB formation, we analyzed K division in plasticity factor-deficient backgrounds. We could not

CellPress
OPEN ACCESS

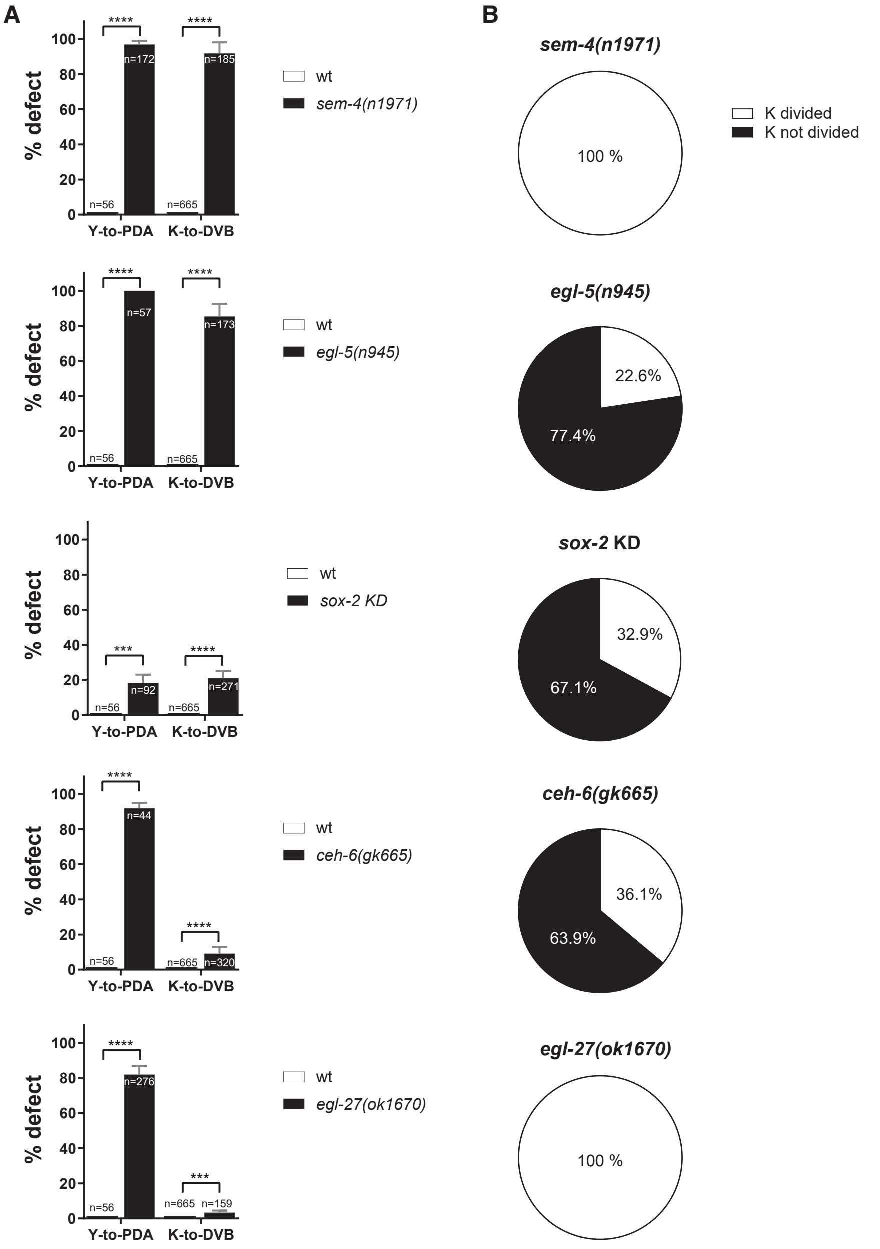

Figure 4. Some key Y-to-PDA plasticity factors are also required for K-to-DVB

(A) Quantification of “absence of PDA” (observed with *exp-1p::gfp)* and “absence of DVB” (observed with *unc-47p::gfp*) defects in wild-type L4 versus mutants or deficient background for Y-to-PDA key plasticity factors. n, total animals scored. See also Figures S4, S6, and S7. Mean and standard deviation between biological replicates of the percentage of worms scored are represented. *p < 0.05; **p < 0.01; ***p < 0.001; ****p < 0.0001, and ns, not significant.

(B) Quantification of K division in the animals deficient for key plasticity factors presented in A (black bars for K-to-DVB) and showing an “absence of DVB” phenotype. Two hundred seventy-six, 87, 221, 127, and 160 animals were scored in *sem-4*, *egl-5*, *sox-2*, *ceh-6*, and *egl-27* mutants, respectively.

assess its orientation and asymmetry in *egl-5*, *ceh-6*, or *sox-2* mutant backgrounds because of technical limitations (see STAR Methods). However, we observed that the absence of DVB in *egl-5* null, *ceh-6* rectal-specific mutant, and *sox-2* knockdown using the anti-GFP nanobody strategy (Wang et al., 2017) is due to K not dividing in three-quarters (*egl-5*) and two-thirds (*ceh-6* and *sox-2*) of the cases (Figure 4B). When K divides but DVB does not develop, K.p retains the rectal identity, as visualized by *col-34* expression in L4 larvae (not shown). Conversely, normally oriented K division occurs in 100% of *sem-4* null animals (Figures 2D and 4B), despite >90% of “no DVB” defect. In *sem-4* mutant, K.p persistently exhibits a large nucleus (Figure 2E), reminiscent of an epithelial identity, in agreement with Basson and Horvitz (1996), and retains the epithelial and rectal markers *lin-26*, *let-413*, *col-34*, and *egl-5* in L3 and L4 stages (Figures S4B and S4D; Table S1). *ajm-1* is also expressed, suggesting that this apical gene is never silenced or is re-expressed in K.p (Figures S4A and S5; Table S1), while neuronal markers are not expressed in K.p in *sem-4* mutant (Figures S4E–S4I; Table S1).

Our findings suggest two roles for the plasticity factors in K-to-DVB: (1) allow the occurrence of K division (*egl-5*, *ceh-6*, and *sox-2*) and (2) initiate K.p reprogramming through the erasure of its rectal identity, very reminiscent of their role during Y-to-PDA.

Plasticity factors and the Wnt signaling pathway act in parallel to erase K.p rectal identity

We investigated the interaction between Y-to-PDA factors and the Wnt signaling pathway during K-to-DVB. We focused on *sem-4* mutant and its relationship with the Wnt pathway, as *sem-4* and *lin-17* mutants show similar phenotypes. The Wnt pathway might control *sem-4* expression in K.p, as it was demonstrated that TCF/LEF1 can bind to the *SALL4* promoter in human cell lines (Böhm et al., 2006). However, the expression of a *sem-4* KI-reporter is not affected in K.p or in other rectal cells of *lin-17* mutant (Figure S7A). In addition, a *sem-4* translational construct able to rescue *sem-4* phenotype was not capable to rescue the “no DVB” defect of *lin-17* mutant (92.5% “no DVB” defect in the *lin-17* mutant with the *sem-4* rescuing construct [n = 204] versus 92.4% in the *lin-17* mutant alone [n = 42]). Thus, neither *sem-4* expression nor activity is downstream of the Wnt signaling in K.p. We tested the reciprocal relationship

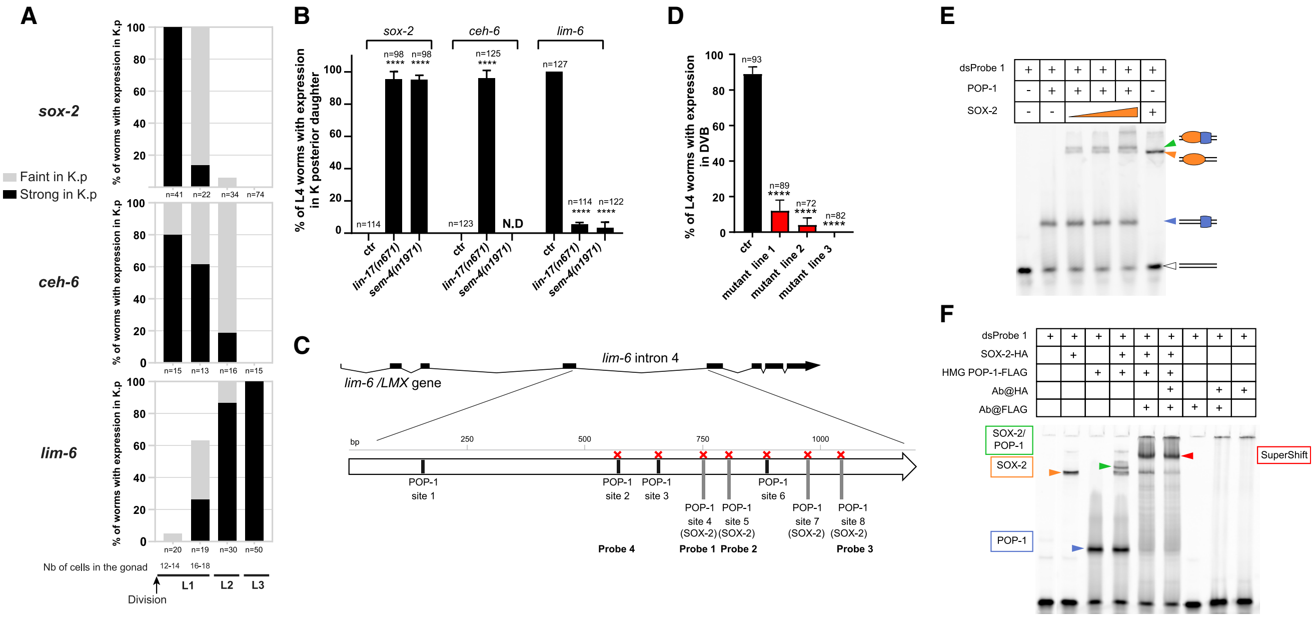

Figure 5. *lim-6* expression regulation by SOX-2 and POP-1/TCF

(A) Time course expression of *sox-2*, *ceh-6*, and *lim-6* CRISPR-KI reporters in K.p right after K division in L1, in early L2, and in L3 (DVB). L1s worms were tightly synchronized on the basis of the number of cells in the gonad. Black bars represent strong signal in K.p, and gray bars represent faint signal in K.p. See also Figures S8 and S10.

(B) *sox-2*, *ceh-6*, and *lim-6* CRISPR-KI reporter expression in L4 in wild-type (DVB), *lin-17(n671)*, and *sem-4(n1961)* mutant backgrounds (persistent K.p). *ceh-6* CRISPR-KI expression could not be addressed in the *sem-4* mutant because of genetic linkage of both genes. n, total animals scored. Mean and standard deviation between biological replicates of the percentage of worms scored are represented. *p < 0.05; **p < 0.01; ***p < 0.001; ****p < 0.0001, and ns, not significant.

(C) Genomic organization of *lim-6/LMX* and POP-1/TCF and SOX-2 binding sites in the fourth intron. The probes used in this study are represented. See also Figure S9. Red crosses, mutated binding sites in the construct used in the transgenic lines presented in D.

(D) Expression in DVB of the *lim-6* intron 4 transcriptional reporter depends on TCF sites. One transgenic line bearing the wild-type version of the intron 4 (ctrl) and three independent transgenic lines bearing the mutated intron 4 (TCF sites 2–8) were analyzed in parallel in L4 larvae. In (B) and (D), bars represent mean ± SD. n, total animals scored. Mean and standard deviation between biological replicates of the percentage of worms scored are represented. *p < 0.05; **p < 0.01; ***p < 0.001; ****p < 0.0001, and ns, not significant.

(E and F) Gel shift assays using the purified full-length SOX-2 and HMG-POP-1 with CY-5-labeled double stranded DNA probe 1. Binding of HMG-POP-1 (blue arrowhead) was observed on the probe in the absence of SOX-2, and vice versa (SOX-2 on probe, orange arrowhead).

(E) Increasing concentrations of SOX-2 (125, 250, and 500 nM) were added to the dsProbe and HMG-POP-1and translated into increased binding to the probes (dark orange arrowhead) as well as an upper shift (green arrowhead) most probably corresponding to a HMG-POP-1-SOX-2-Probe complex. Double line, free probe; double lines with blue square, orange oval or both represent the HMG-POP-1-Probe, the SOX-2-Probe and a SOX-2-HMG-POP-1-Probe complex, respectively. (F) Addition of the anti-FLAG antibody resulted in a supershifted complex containing the DNA probe, SOX-2, HMG-POP-1, and the antibody (red arrowhead).

See also Figures S11, S12, and S13.

with a *lin-17* transcriptional reporter and observed *lin-17* faint expression in K.p as in K.a in *sem-4(n1971)* and wild-type (not shown), which disappears in K.p as it becomes DVB in the wild-type, but not in the mutant (Figure S7B). This is consistent with maintenance of the rectal identity of K.p in *sem-4* mutant, as *lin-17* is a rectal marker. Indeed, *lin-17* expression also persists in *lin-17* mutant in K.p, which also remains rectal (Figure S7B).

We examined the genetic relationship between the Wnt pathway and the plasticity factors with double mutants, using *wrm-1*/β-cat *(n1982 ts)* allele for the Wnt pathway (because *lin-17[n671]* alone shows high penetrance of DVB absence), and *sem-4* hypomorphic allele *n1378* that shows a low "no DVB" defect (Figure S7C) rather than *n1971* null allele. The double mutant *sem-4(n1378)*; *wrm-1(n1982 ts)* led to a synergistic "no DVB" defect compared with the single mutants at 25°C (Figure S7C). Moreover, we observed the same synergy in double mutants with downregulated *sox-2* and *wrm-1* activities (Figure S7D).

Altogether our results suggest that the plasticity factors and the Wnt signaling pathway act in two different parallel genetic pathways to control the loss of K.p rectal identity.

Antiparallel activities of *sox-2/ceh-6* and the Wnt signaling may control the timing of re-differentiation

We characterized the mechanisms regulating re-differentiation into DVB. We focused on *lim-6* gene, encoding a LIM homeobox TF and the sole identified DVB terminal selector, required for DVB terminal differentiation (Hobert et al., 1999; Hober, 2016). Using both a rescuing construct and a KI reporter, we observed

that *lim-6* is expressed in K.p in about 25% L1 wild-type larvae 1–2 h after K division (Figure 5A), overlapping with the decreasing expression of *lin-26* and *let-413* (Figure S2B). A transcriptional reporter bearing the intron 4 of *lim-6*, sufficient for the expression in DVB, follows the same transcriptional dynamics in K.p as the KI (Figures S8A–S8C) and the *otIs157* (Hobert et al., 1999) reporters. Thus, *lim-6* expression appears to be an early indicator of DVB future identity (Figures 5A and S2B). Consistently, *lim-6* is not expressed in K.p in *sem-4* and *lin-17* mutants (Figure 5B).

The earlier expression of *lim-6* in K.p compared with other neuronal markers led us to hypothesize that it could be directly downstream of the Wnt signaling, through POP-1 binding to *lim-6* regulatory regions. Indeed, *Lmx1*, the *lim-6* mouse ortholog, is regulated by the Wnt signaling in proliferative dopaminergic neuron progenitors (Joksimovic et al., 2009, 2012; Chilov et al., 2010). We found putative POP-1/TCF binding sites in the intron 4 of *lim-6* (see STAR Methods; Figure S9B), 8 conserved across nematode species (Figures 5C, S9A, and S9C). When *gfp* reporter constructs lacking the regions encompassing sites 3–6 or 3–8 were injected in the worm, expression was absent in DVB. To test whether those sites are specifically required for expression during the K-to-DVB, we mutated them (Arata et al., 2006), focusing on the conserved sites 2–8 (Figure 5C, red crosses). Transgenic animals bearing the mutated constructs showed almost no expression in DVB (Figure 5D), suggesting that *lim-6* is directly, positively regulated by the Wnt signaling during K-to-DVB.

POP-1/TCF binding sites may overlap with SOX2 binding sites, as both TCF and SOX2 are HMG domain proteins (Figure S9D; Lin et al., 1995; Pevny and Lovell-Badge, 1997). Indeed, 4 POP-1/TCF binding sites in the intron 4 of *lim-6* overlap with SOX2 binding sites (Figures 5C and S9A; STAR Methods). Expression dynamics of key players suggest a functional relationship between SOX-2 and POP-1: the rectal markers *sox-2* and *ceh-6* display opposite expression dynamics with respect to *lim-6* in K.p (Figures 5A and S10A), resulting in a complete absence of expression in DVB (Table S1). The early onset of *lim-6* expression in K.p and its anti-correlation to *sox-2* and *ceh-6* expression led us to hypothesize a competition between SOX-2(/CEH-6) and POP-1 regulating *lim-6* expression. Thus, we examined if both POP-1 and SOX-2 could bind to *lim-6* intron 4 sequences. Electrophoresis mobility shift assays (EMSAs) using DNA probes corresponding to TCF/SOX2 binding *sites 2*, *4*, *5*, and *8* in *lim-6* intron 4 (Figures S9A and S9E) showed that both SOX-2 and HMG-POP-1 can bind these sequences alone (Figure S11) and together (see retarded band in Figures 5E, 5F, and S12 and super-shifted bands when antibodies specific to either SOX-2 or POP-1 are used in Figure S13). It is possible that SOX-2 and POP-1 display antagonistic activities on *lim-6* activation depending on their expression levels and nuclear localization over time. Indeed, preventing decrease of *sox-2* in K.p through overexpression of a *sox-2* transgene resulted in significant loss of *lim-6* expression in DVB (Figure S10B).

In sum, our results suggest that the Wnt pathway leads to downregulation of *ceh-6* and *sox-2* rectal genes in K.p, the latter having a repressive function on *lim-6* expression, and in parallel triggers the activation of *lim-6* through direct binding of POP-1 to *lim-6* regulatory regions (Figure 6C).

DISCUSSION

We addressed the role of cell division and the existence of conserved mechanisms during natural Td by characterizing K-to-DVB, a Td event occurring during *C. elegans* development through a cell division. We first demonstrated that the K rectal cell is a fully differentiated and specialized cell, with all characteristics of the end-of-lineage rectal identity. Yet it divides once and for this reason is considered a blast cell (Chisholm, 1991). However, K is not a stem cell but a mature rectal cell, indistinguishable also at the transcriptional level (Packer et al., 2019) from the other rectal cells that do not divide or change identity and with which it forms the rectum. Thus, although not post-mitotic, K holds a structural role in a permanent, vital organ and is fully differentiated. Few other terminally differentiated cells can divide, such as hepatocytes, which form similar hepatocyte daughters (Miyaoka and Miyajima, 2013). By contrast, the rectal K cell gives rise to two daughters, one subsequently adopting a distinct, differentiated identity. The ability of a differentiated cell to give rise to another differentiated cell type was termed transdifferentiation (Eguchi and Kodama, 1993; Lambert et al., 2021) and K-to-DVB meets the criteria defining Td.

Cell division is required for K-to-DVB and may contribute to reprogramming through different mechanisms

Using mutants defective for K division we demonstrated that cell division is a key step for DVB formation. How might it contribute? One mechanism might involve the asymmetric partitioning of cellular components (Betschinger and Knoblich, 2004). However, our data do not point to such mechanism: plasticity factors and most of the tested epithelial and rectal markers are not asymmetrically partitioned (POP-1/TCF and SYS-1/β-cat expression levels are too low in K cell and its daughters to analyze them). Only some apical membrane proteins are asymmetrically distributed (in K.a), but the observation that K.a can turn into a DVB-like cell in WNT mutants suggests that these proteins do not affect the competence of K daughters to transdifferentiate.

Cell division can contribute to reprogramming also through DNA replication (Nashun et al., 2015) or the formation of a more epigenetically plastic environment, by facilitating the erasure of pre-existing chromatin marks and/or open access to chromatin. Indeed, chromatin regulators can act as barriers to induced direct reprogramming (Hajduskova et al., 2019; Kolundzic et al., 2018; Rothman and Jarriault, 2019). Even though our analysis suggests that DNA replication alone is not sufficient to trigger a dramatic transcriptional change and the erasure of K rectal identity, DNA replication might facilitate Td, for instance by dispensing with some of the factors required for Td events in which DNA replication does not occur. In support of this, we found that *egl-27/MTA*, an ortholog of a chromatin remodeling complex component (Kumar and Wang, 2016), is not key for K-to-DVB, whereas it is very significantly required for Y-to-PDA in absence of cell division (Kagias et al., 2012).

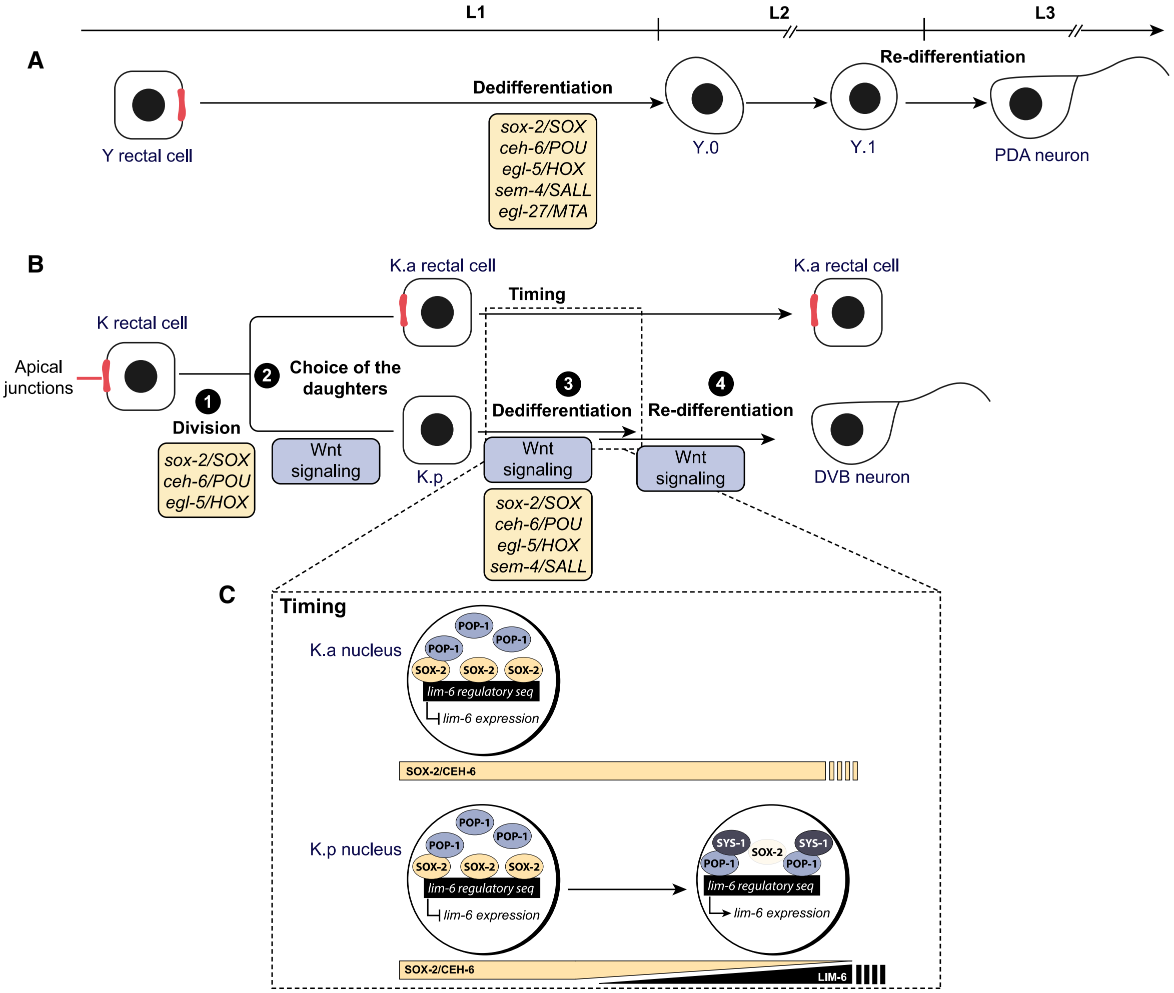

Figure 6. Model for K-to-DVB Td and parallel with Y-to-PDA

(A) Y-to-PDA Td, initiated end of the L1 stage and finalized in the L3 stage, goes through two intermediate states: Y.0, which appears to have lost characteristics of the initial identity but not have gained those of the final identity, and Y.1, which appears to be an early neuronal cell. The box describes the cell plasticity cassette factors required for the initiation of Y-to-PDA Td, that is, the dedifferentiation step. Top: developmental timeline for A and B.

(B) K-to-DVB Td, initiated toward the end of the L1 stage and finalized in the L2 stage, involves a cell division and an intermediate state that possibly represents a mixed identity between the initial and the final identity. The four important features of this Td, as well as the factors involved, are highlighted: division, choice of which daughter will adopt a different identity, loss of the initial identity and adoption of a subtype-specific final identity.

(C) Inset representing how the dynamics of SOX-2 and POP-1 levels and their competition in modulating *lim-6* expression can provide a timer for the re-differentiation step.

The Wnt/β-catenin asymmetry pathway mediates several aspects of K-to-DVB

Our data support the involvement of the *C. elegans* Wnt/β-catenin asymmetry pathway in K-to-DVB. The Wnt/β-catenin asymmetry pathway influences cell identity specification in several cellular contexts and at different developmental stage in *C. elegans*, ranging from the 2-cell stage to late embryonic development and to larval development (Mizumoto and Sawa, 2007; Bertrand and Hobert, 2009; Sawa and Korswagen, 2013). Our study shows that the Wnt pathway can also contribute to Td of a fully differentiated cell in multiple ways: the choice of which daughter will convert to a different identity, the erasure of the rectal identity, and the definition of DVB subtype-specific identity (Figure 6B).

We showed that posterior LIN-44/WNT ensures that a daughter cell is formed at a stereotyped posterior-basal position and is able to lose its mother identity. In its absence, other more anteriorly expressed WNT ligands can induce K-to-DVB, as observed in seam cells (Jackson and Eisenmann, 2012; Yamamoto et al., 2011). This would explain both the non-fully penetrant ‘‘no DVB’’ defect and the reversed polarity of cell division in *lin-44* mutants. We propose that the Wnt signaling pathway has an instructive, rather than a permissive role on K-to-DVB, with WNT ligands acting as positional cues and instructing which K daughter will transdifferentiate. An analogous situation was demonstrated in the SMDD/AIY asymmetric cell division during embryonic development (Kaur et al., 2020).

Besides the Wnt pathway, physical constraints due to the integration of the K cell into the rectum likely affect the orientation of K division, similarly to the role of cell shape and the Wnt pathway previously described (Wildwater et al., 2011). This might explain why the mutants that we tested (*gpr-1*, *goa-1*, and *gpa-16* mutants) did not show any significant impact on K division orientation, and why the orientation defect in *lin-17/FZD* mutant is also very low compared with its "no DVB" defect. Irrespective of K division orientation, in the absence of the Wnt signaling, K.p remains a rectal cell, suggesting that another important role of the Wnt signaling is to erase the epithelial identity of K.p, reminiscent of Y dedifferentiation step. A role in Td for Wnt was recently proposed in promoting hepatocyte-to-cholangiocyte Td (Kosar et al., 2020), and in converting biliary cells (BECs) into hepatocytes in mouse models with compromised hepatocyte proliferation (Valle-Encinas et al., 2020). Further similarities between BEC-to-hepatocyte conversion and the K.p-to-DVB transition include the transition through a mixed identity (Deng et al., 2018). It would be interesting to assess if the role of WNT signal in promoting the erasure of the initial identity in Td is conserved. Interestingly, the Wnt pathway promotes not only dedifferentiation but also re-differentiation, through the instruction of DVB-specific identity: the Wnt pathway is required for expression of the LIM-6 terminal selector TF.

Although we could not easily assess whether WNT distinct functions are sequential, in support of sequential outputs, the WNT signal has been shown to have opposite effect on embryonic stem cells depending on the cell state (Merrill, 2012). Future studies, for instance looking at the evolution over time of the transcriptome of the K.p cell, may address this question.

Conserved reprogramming TFs are required for K-to-DVB

We showed that the Y-to-PDA factors SEM-4, EGL-5, SOX-2, and CEH-6 are also required for K-to-DVB to erase K.p rectal identity after division and, except SEM-4, for K division to occur (Figures 6A and 6B). Thus, K division is necessary but not sufficient for the formation of the DVB neuron: in *sem-4* mutant, and *sox-2* and *ceh-6* mutants in which the K cell did divide, K.p appears to remain rectal. Our data are in agreement with the previously postulated role for *sox-2* for the terminal neural differentiation of post-embryonic differentiated cells, also known as Td (Vidal et al., 2015), with a difference: the lack of *ceh-6* expression (used as rectal cell marker) led Vidal et al. to conclude that the K.p cell found in *sox-2* mutants does not maintain the rectal identity, contrary to what *col-34* expression observed here suggests. We propose that lack of *ceh-6* expression in K.p in *sox-2* mutant might be due to the continued activity of the Wnt signaling pathway, which is required for *ceh-6* downregulation, rather than reflecting a loss of rectal identity.

The involvement of Y-to-PDA factors in K-to-DVB reinforces the hypothesis that shared mechanisms exist (Figures 6A and 6B) and that those factors might be part of a conserved "plasticity cassette" in *C. elegans*, allowing reprogramming initiation of differentiated cells. Interestingly, these genes belong to families known to be master regulators of cell fate and their reprogramming capacity is conserved through evolution (Julian et al., 2017; Malik et al., 2018; Tsubooka et al., 2009). It remains to be determined if these factors are involved in other natural Td events, or if region or tissue-specific variations exist. A recent study reported a low penetrance defect in the AMso-to-MCM or the PHso-to-PHD conversions in mosaic worms having lost a *sox-2* rescuing array, although *sem-4* and *sox-2* genes appeared little or not involved using systemic RNAi (Molina-García et al., 2020). Thus, this study and the present one underscore the importance of the approach used to eliminate *sox-2* activity: *sox-2* systemic RNAi, even in a sensitized background, did not result in significant K-to-DVB defects (not shown) by contrast with cell-specific expression of a *sox-2* antisense RNA. It is also conceivable that variations around this set of factors exist between Td events, with the involvement of other family members.

The plasticity TFs are expressed in all rectal cells, including those that never change their identity. Although the Wnt pathway has an instructive role on K-to-DVB, our data are consistent with the TFs *sem-4*, *sox-2*, *ceh-6*, and *egl-5* providing a permissive cellular context for reprogramming of K, as we previously proposed for Y-to-PDA (Kagias et al., 2012). We anticipate that other factors are expressed in Y and K and specifically allow reprogramming (e.g., ZTF-11; Lee et al., 2019) and that factors expressed in the other rectal cells might prevent their plasticity.

The Wnt pathway and plasticity TFs cooperate to erase K.p rectal epithelial identity and to control the timing of re-differentiation into DVB

Our characterization of the relationship between the Wnt signaling and the reprogramming TFs suggests that they act in parallel to allow the erasure of K.p rectal features and its conversion into DVB. Such cooperation between the Wnt pathway and these TFs was described in other contexts and appears conserved: *sem-4* and the β-catenin *bar-1* cooperate to render six epidermal precursor cells competent to respond to other developmental signals (Eisenmann and Kim, 2000; Grant et al., 2000), and the Wnt signaling enhances the pluripotent reprogramming capacities of SOX2, KLF4, and OCT4 (Marson et al., 2008).

We found that a likely consequence of their interplay is the timing of expression of the terminal selector *lim-6*. Examination of DVB enhancer in *lim-6* intron 4 revealed several POP-1/TCF binding sites, necessary for *lim-6* expression in DVB and bound by the POP-1 protein. In addition, our observations that *sox-2/SOX* and *ceh-6/OCT* expression is downregulated in K.p, in an anti-correlated fashion to the beginning of the expression of *lim-6*, suggest that the Wnt pathway and at least these two members of the plasticity cassette act antagonistically on *lim-6* expression, and hence DVB neuron subtype identity acquisition. Overexpression of SOX-2 in K.p blocks *lim-6* expression in DVB, consistently with antagonistic activities of Wnt and SOX proteins in a TOP-flash assay (Sinner et al., 2007). We found that SOX-2, whose consensus overlaps POP-1/TCF's (Lin et al., 1995; Pevny and Lovell-Badge, 1997), is able to bind to several of POP-1/TCF sites. On the basis of our EMSA experiments, we propose that SOX-2 and POP-1, rather than competing for the binding on

lim-6 regulatory region, co-occupy those sites and that SOX-2 might negatively affect POP-1 activity directly or by sequestering co-factors such as β-catenin. Such a model is consistent with studies in vertebrates, in which SOX factors were shown to interact with TCF/β-catenin on DNA or to affect β-catenin stability (Akiyama, 2004; Kormish et al., 2010; Mukherjee et al., 2020), and also with a recent model postulating that SOX2 levels influence the cell response to the Wnt signaling, leading to pro-pluripotency (at high levels) or pro-differentiation activities (Blassberg et al., 2022). The negative interactions between SOX-2 and the Wnt signaling (a third role for *sox-2* in K Td) might further be reinforced by CEH-6/OCT, as OCT4 was shown to inhibit TCF/β-catenin stability and transcriptional activity in mouse ES cells and Xenopus (Cao et al., 2007; Abu-Remaileh et al., 2010; Davidson et al., 2012). As SOX-2 and CEH-6 levels go down, this negative regulation of SOX-2 on the Wnt pathway would be released and *lim-6* expression activated. Downregulation of *sox-2* and *ceh-6* is lost in *lin-17/FZD* mutant, suggesting negative feedbacks. We postulate that this dynamic pas de deux between *sox-2* and the Wnt signaling provides a timer for precise *lim-6* expression in K.p and therefore re-differentiation (Figure 6C).

The transition path may differ between different transdifferentiation events

K-to-DVB Td occurs in a shorter time window compared with Y-to-PDA Td (less than 6 h compared with an entire larval stage), likely affecting the nature of the conversion. Indeed, our analysis of the expression of cell identity marker genes suggests that the discrete cellular steps observed during Y-to-PDA, with a transient dedifferentiated intermediate with no overlapping rectal and neuronal markers, are blurred in this context. Whether Td proceeds through a discontinuous, step-and-go model, or through a smooth continuous model (Lambert et al., 2021), and how this compares with developmental differentiation trajectories, remain open questions. Taken together, our data on K-to-DVB (this study) and Y-to-PDA (Richard et al., 2011) suggest that both continuous and discontinuous modes may exist during Td in *C. elegans*. Such diversity is also seen in reprogramming events in other organisms: transcriptomic analyses of limb regeneration (Gerber et al., 2018), B-to-macrophage cells (Di Tullio et al., 2011; Francesconi et al., 2018), pericyte-to-neuron (Karow et al., 2018), or fibroblast-to-myotube (Cacchiarelli et al., 2018) reprogramming suggest that these processes occur via a discontinuous step-and-go model (Di Tullio et al., 2011; Gerber et al., 2018). In contrast, during fibroblast-to-neuron Td, Treutlein et al. (2016) describe a continuous path with concomitant loss of fibroblast and gain of neuronal gene expression, which nevertheless involves transition through a distinct state expressing a subset of neural progenitor genes. Future studies with finer granularity in sampling times within the considered time window will further illuminate the range of paths and transition states when cells swap identities.

Limitations of the study

One limitation of this study is the difficulty to knock down the activity of genes required for embryonic and early larval viability and still be able to grow animals until Td has occurred. In addition, rectal cells are insensitive to RNAi in wild-type background. To bypass early lethality, we engineered several rectal-specific knockdown approaches, such as expression of an antisense sequence in a RNAi-sensitized background, or the use of the GFP-targeting nanobodies. However, these approaches are not fully efficient in rectal cells, thus possibly minimizing the real effect. Future studies will address these issues and try to include a temporal control component, as some of the investigated genes have successive roles in both K division and K.p conversion. Another limitation is the current unfeasibility of chromatin immunoprecipitation (ChIP) in one unique cell in *C. elegans*, together with the difficulty in isolating a transient unique cell. It is likely that the methods being developed will allow to address such chromatin-related questions in the future.

STAR★METHODS

Detailed methods are provided in the online version of this paper and include the following:

- KEY RESOURCES TABLE
- RESOURCE AVAILABILITY
  - Lead contact
  - Materials availability
  - Data and code availability
- EXPERIMENTAL MODEL AND SUBJECT DETAILS
  - Construction of *C. elegans* strains
  - Plasmid construction
- METHOD DETAILS
  - RNAi/silencing experiments
  - Epifluorescence microscopy
  - Confocal/spinning disk microscopy
  - Scoring criteria
  - Electron microscopy
  - Image processing and analysis
  - smFISH
  - Transcription factor binding sites analysis
  - 6×His-tagged protein expression and purification
  - Electrophoretic mobility shift assay (EMSA) and super shift assay
- QUANTIFICATION AND STATISTICAL ANALYSIS
  - Statistical analysis and data representations
  - Quantification of nuclear volumes

SUPPLEMENTAL INFORMATION

Supplemental information can be found online at https://doi.org/10.1016/j.celrep.2022.111365.

ACKNOWLEDGMENTS

We are grateful to J. Godin, O. Hobert, A. Alcolei, and M. Barkoulas for their insightful comments on the manuscript. Some strains were provided by the Caenorhabditis Genetics Center (funded by the NIH Office of Research Infrastructure Programs [P40 OD010440]). We thank M. Labouesse for reagents, C. Delance for rectal membrane reporter, A. Daulny and the Institut de Génétique et de Biologie Moléculaire et Cellulaire (IGBMC) protein purification facility, T. Ye for help with FIMO, Y. Schwab for help with the EM data, the IGBMC

imaging platform for their help with the acquisition of smFISH images, and C. Lloret-Fernandez for the original cell design of Figure 6. This work was supported by the ANR-10-LABX-0030-INRT grant managed by Agence Nationale de la Recherche under the Investissements d'Avenir frame program ANR-10-IDEX-0002-02, including an IGBMC PhD Programme fellowship to C.R.; as well as by Ligue Nationale Contre le Cancer, ANR (Agence Nationale de la Recherche) CELLSwitch #ANR-13-BSV2-0005, and ERC CoG (European Research Council Consolidator Grant) PlastiCell #648960 grants awarded to S.J.; M.H. was supported by a Ligue Contre le Cancer postdoctoral fellowship; C.G. is a University of Strasbourg assistant professor; and S.J. is a CNRS research director.

AUTHOR CONTRIBUTIONS

Investigation, C.R., M.H., C.G., A.A., and S.J.; Resources, C.R., M.H., C.G., and A.A.; Validation, C.R., M.H., and C.G.; Visualization, C.R. and C.G.; Conceptualization, C.R., M.H., C.G., and S.J.; Formal Analysis, C.R., M.H., C.G., and S.J.; Data Curation, C.G.; Writing, C.R., C.G., and S.J.; Supervision, S.J.; Funding Acquisition, S.J.; Project Administration, S.J.

DECLARATION OF INTERESTS

The authors declare no competing interests.

Received: August 16, 2021
Revised: April 9, 2022
Accepted: August 25, 2022
Published: September 20, 2022

REFERENCES

Abu-Remaileh, M., Gerson, A., Farago, M., Nathan, G., Alkalay, I., Zins Rousso, S., Gur, M., Fainsod, A., and Bergman, Y. (2010). Oct-3/4 regulates stem cell identity and cell fate decisions by modulating Wnt/β-catenin signalling. EMBO J. *29*, 3236–3248.

Ahier, A., Suman, S.K., and Jarriault, S. (2020). Gene bashing of ceh-6 locus identifies genomic regions important for ceh-6 rectal cell expression and rescue of its mutant lethality. MicroPubl. Biol. *2020*.

Akiyama, H., Lyons, J.P., Mori-Akiyama, Y., Yang, X., Zhang, R., Zhang, Z., Deng, J.M., Taketo, M.M., Nakamura, T., Behringer, R.R., et al. (2004). Interactions between Sox9 and -catenin control chondrocyte differentiation. Genes Dev. *18*, 1072–1087.

Arata, Y., Kouike, H., Zhang, Y., Herman, M.A., Okano, H., and Sawa, H. (2006). Wnt signaling and a hox protein cooperatively regulate PSA-3/meis to determine daughter cell fate after asymmetric cell division in C. elegans. Dev. Cell *11*, 105–115.

Basson, M., and Horvitz, H.R. (1996). The Caenorhabditis elegans gene sem-4 controls neuronal and mesodermal cell development and encodes a zinc finger protein. Genes Dev. *10*, 1953–1965.

Bertrand, V., and Hobert, O. (2009). Linking asymmetric cell division to the terminal differentiation program of postmitotic neurons in C. elegans. Dev. Cell *16*, 563–575.

Betschinger, J., and Knoblich, J.A. (2004). Dare to Be different: asymmetric cell division in Drosophila, C. elegans and vertebrates. Curr. Biol. *14*, R674–R685.

Blassberg, R., Patel, H., Watson, T., Gouti, M., Metzis, V., Delás, M.J., and Briscoe, J. (2022). Sox2 levels regulate the chromatin occupancy of WNT mediators in epiblast progenitors responsible for vertebrate body formation. Nat. Cell Biol. *24*, 633–644.

Böhm, J., Sustmann, C., Wilhelm, C., and Kohlhase, J. (2006). SALL4 is directly activated by TCF/LEF in the canonical Wnt signaling pathway. Biochem. Biophys. Res. Commun. *348*, 898–907.

Brenner, S. (1974). The genetics of Caenorhabditis elegans. Genetics *77*, 71–94.

Brockes, J.P., and Kumar, A. (2002). Plasticity and reprogramming of differentiated cells in amphibian regeneration. Nat. Rev. Mol. Cell Biol. *3*, 566–574.

Bürglin, T.R., and Ruvkun, G. (2001). Regulation of ectodermal and excretory function by the C. elegans POU homeobox gene ceh-6. Development *128*, 779–790.

Cabello, J., Neukomm, L.J., Günesdogan, U., Burkart, K., Charette, S.J., Lochnit, G., Hengartner, M.O., and Schnabel, R. (2010). The Wnt pathway controls cell death engulfment, spindle orientation, and migration through CED-10/Rac. PLoS Biol. *8*, e1000297.

Cacchiarelli, D., Qiu, X., Srivatsan, S., Manfredi, A., Ziller, M., Overbey, E., Grimaldi, A., Grimsby, J., Pokharel, P., Livak, K.J., et al. (2018). Aligning single-cell developmental and reprogramming trajectories identifies molecular determinants of myogenic reprogramming outcome. Cell Syst. *7*, 258–268.e3.

Cadart, C., Zlotek-Zlotkiewicz, E., Le Berre, M., Piel, M., and Matthews, H.K. (2014). Exploring the function of cell shape and size during mitosis. Dev. Cell *29*, 159–169.

Cao, Y., Siegel, D., Donow, C., Knöchel, S., Yuan, L., and Knöchel, W. (2007). POU-V factors antagonize maternal VegT activity and β-Catenin signaling in Xenopus embryos. EMBO J. *26*, 2942–2954.

Chilov, D., Sinjushina, N., Saarimäki-Vire, J., Taketo, M.M., and Partanen, J. (2010). beta-Catenin regulates intercellular signalling networks and cell-type specific transcription in the developing mouse midbrain-rhombomere 1 region. PLoS One *5*, e10881.

Chisholm, A. (1991). Control of cell fate in the tail region of C. elegans by the gene egl-5. Development *111*, 921–932.

Corish, P., and Tyler-Smith, C. (1999). Attenuation of green fluorescent protein half-life in mammalian cells. Protein Eng. *12*, 1035–1040.

Davidson, K.C., Adams, A.M., Goodson, J.M., McDonald, C.E., Potter, J.C., Berndt, J.D., Biechele, T.L., Taylor, R.J., and Moon, R.T. (2012). Wnt/-catenin signaling promotes differentiation, not self-renewal, of human embryonic stem cells and is repressed by Oct4. Proc. Natl. Acad. Sci. USA *109*, 4485–4490.

Deng, X., Zhang, X., Li, W., Feng, R.-X., Li, L., Yi, G.-R., Zhang, X.-N., Yin, C., Yu, H.-Y., Zhang, J.-P., et al. (2018). Chronic liver injury induces conversion of biliary epithelial cells into hepatocytes. Cell Stem Cell *23*, 114–122.e3.

Di Tullio, A., and Graf, T. (2012). C/EBPα bypasses cell cycle-dependency during immune cell transdifferentiation. Cell Cycle *11*, 2739–2746.

Di Tullio, A., Manh, T.P.V., Schubert, A., Castellano, G., Månsson, R., and Graf, T. (2011). CCAAT/enhancer binding protein (C/EBP)-induced transdifferentiation of pre-B cells into macrophages involves no overt retrodifferentiation. Proc. Natl. Acad. Sci. USA *108*, 17016–17021.

Eguchi, G., and Kodama, R. (1993). Transdifferentiation. Curr. Opin. Cell Biol. *5*, 1023–1028.

Eguchi, G., Eguchi, Y., Nakamura, K., Yadav, M.C., Millán, J.L., and Tsonis, P.A. (2011). Regenerative capacity in newts is not altered by repeated regeneration and ageing. Nat. Commun. *2*, 384.

Eisenmann, D.M., and Kim, S.K. (2000). Protruding vulva mutants identify novel loci and Wnt signaling factors that function during Caenorhabditis elegans vulva development. Genetics *156*, 1097–1116.

Ferreira, H.B., Zhang, Y., Zhao, C., and Emmons, S.W. (1999). Patterning of Caenorhabditis elegans posterior structures by theAbdominal-B homolog, egl-5. Dev. Biol. *207*, 215–228.

Fishman, V.S., Shnayder, T.A., Orishchenko, K.E., Bader, M., Alenina, N., and Serov, O.L. (2015). Cell divisions are not essential for the direct conversion of fibroblasts into neuronal cells. Cell Cycle *14*, 1188–1196.

Francesconi, M., Di Stefano, B., Berenguer, C., de Andrés-Aguayo, L., Plana-Carmona, M., Mendez-Lago, M., Guillaumet-Adkins, A., Rodriguez-Esteban, G., Gut, M., Gut, I.G., et al. (2018). Single cell expression analysis uncouples transdifferentiation and reprogramming. Elife *8*, e41627. https://doi.org/10.7554/eLife.41627.

Gerber, T., Murawala, P., Knapp, D., Masselink, W., Schuez, M., Hermann, S., Gac-Santel, M., Nowoshilow, S., Kageyama, J., Khattak, S., et al. (2018). Single-cell analysis uncovers convergence of cell identities during axolotl limb regeneration. Science *362*, eaaq0681.

Goldstein, B., Takeshita, H., Mizumoto, K., and Sawa, H. (2006). Wnt signals can function as positional cues in establishing cell polarity. Dev. Cell *10*, 391–396.

Gotta, M., and Ahringer, J. (2001). Axis determination in C. elegans: initiating and transducing polarity. Curr. Opin. Genet. Dev. *11*, 367–373.

Grant, K., Hanna-Rose, W., and Han, M. (2000). sem-4 promotes vulval cell-fate determination in Caenorhabditis elegans through regulation of lin-39 hox. Dev. Biol. *224*, 496–506.

Guo, S., Zi, X., Schulz, V.P., Cheng, J., Zhong, M., Koochaki, S.H.J., Megyola, C.M., Pan, X., Heydari, K., Weissman, S.M., et al. (2014). Nonstochastic reprogramming from a privileged somatic cell state. Cell *156*, 649–662.

Hajduskova, M., Baytek, G., Kolundzic, E., Gosdschan, A., Kazmierczak, M., Ofenbauer, A., Beato del Rosal, M.L., Herzog, S., Fatima, N., Mertins, P., et al. (2019). MRG-1/MRG15 is a barrier for germ cell to neuron reprogramming in *Caenorhabditis elegans*. Genetics *211*, 121–139.

Hanna, J., Saha, K., Pando, B., van Zon, J., Lengner, C.J., Creyghton, M.P., van Oudenaarden, A., and Jaenisch, R. (2009). Direct cell reprogramming is a stochastic process amenable to acceleration. Nature *462*, 595–601.

Harterink, M., Kim, D.H., Middelkoop, T.C., Doan, T.D., van Oudenaarden, A., and Korswagen, H.C. (2011). Neuroblast migration along the anteroposterior axis of C. elegans is controlled by opposing gradients of Wnts and a secreted Frizzled-related protein. Development *138*, 2915–2924.

Heppert, J.K., Pani, A.M., Roberts, A.M., Dickinson, D.J., and Goldstein, B. (2018). A CRISPR tagging-based screen reveals localized players in Wnt-directed asymmetric cell division. Genetics *208*, 1147–1164.

Herman, M.A. (2002). Control of cell polarity by noncanonical Wnt signaling in C. elegans. Semin. Cell Dev. Biol. *13*, 233–241.

Herman, M.A., Vassilieva, L.L., Horvitz, H.R., Shaw, J.E., and Herman, R.K. (1995). The C. elegans gene lin-44, which controls the polarity of certain asymmetric cell divisions, encodes a Wnt protein and acts cell nonautonomously. Cell *83*, 101–110.

Hobert, O. (2016). A map of terminal regulators of neuronal identity in *Caenorhabditis elegans: C. elegans* neurodevelopment. Wiley Interdiscip. Rev. Dev. Biol. *5*, 474–498.

Hobert, O., Tessmar, K., and Ruvkun, G. (1999). The Caenorhabditis elegans lim-6 LIM homeobox gene regulates neurite outgrowth and function of particular GABAergic neurons. Development *126*, 1547–1562.

Izumi, Y., Ohta, N., Hisata, K., Raabe, T., and Matsuzaki, F. (2006). Drosophila Pins-binding protein Mud regulates spindle-polarity coupling and centrosome organization. Nat. Cell Biol. *8*, 586–593.

Jackson, B.M., and Eisenmann, D.M. (2012). Beta-catenin-dependent Wnt signaling in C. elegans: teaching an old dog a new trick. Cold Spring Harbor Perspect. Biol. *4*, a007948.

Jarriault, S., Schwab, Y., and Greenwald, I. (2008). A Caenorhabditis elegans model for epithelial-neuronal transdifferentiation. Proc. Natl. Acad. Sci. USA *105*, 3790–3795.

Ji, N., and van Oudenaarden, A. (2012). Single molecule fluorescent in situ hybridization (smFISH) of C. elegans worms and embryos. In WormBook (The C. elegans Research Community, WormBook). https://doi.org/10.1895/wormbook.1.153.1. http://www.wormbook.org.

Joksimovic, M., Yun, B.A., Kittappa, R., Anderegg, A.M., Chang, W.W., Taketo, M.M., McKay, R.D.G., and Awatramani, R.B. (2009). Wnt antagonism of Shh facilitates midbrain floor plate neurogenesis. Nat. Neurosci. *12*, 125–131.

Joksimovic, M., Patel, M., Taketo, M.M., Johnson, R., and Awatramani, R. (2012). Ectopic Wnt/beta-catenin signaling induces neurogenesis in the spinal cord and hindbrain floor plate. PLoS One *7*, e30266.

Julian, L.M., McDonald, A.C., and Stanford, W.L. (2017). Direct reprogramming with SOX factors: masters of cell fate. Curr. Opin. Genet. Dev. *46*, 24–36.

Kagias, K., Ahier, A., Fischer, N., and Jarriault, S. (2012). Members of the NODE (Nanog and Oct4-associated deacetylase) complex and SOX-2 promote the initiation of a natural cellular reprogramming event in vivo. Proc. Natl. Acad. Sci. USA *109*, 6596–6601.

Kamath, R.S., Fraser, A.G., Dong, Y., Poulin, G., Durbin, R., Gotta, M., Kanapin, A., Le Bot, N., Moreno, S., Sohrmann, M., et al. (2003). Systematic functional analysis of the Caenorhabditis elegans genome using RNAi. Nature *421*, 231–237.

Karow, M., Camp, J.G., Falk, S., Gerber, T., Pataskar, A., Gac-Santel, M., Kageyama, J., Brazovskaja, A., Garding, A., Fan, W., et al. (2018). Direct pericyte-to-neuron reprogramming via unfolding of a neural stem cell-like program. Nat. Neurosci. *21*, 932–940.

Kaur, S., Mélénec, P., Murgan, S., Bordet, G., Recouvreux, P., Lenne, P.-F., and Bertrand, V. (2020). Wnt ligands regulate the asymmetric divisions of neuronal progenitors in *C. elegans* embryos. Development *147*, dev183186.

Kolundzic, E., Ofenbauer, A., Bulut, S.I., Uyar, B., Baytek, G., Sommermeier, A., Seelk, S., He, M., Hirsekorn, A., Vucicevic, D., et al. (2018). FACT sets a barrier for cell fate reprogramming in Caenorhabditis elegans and human cells. Dev. Cell *46*, 611–626.e12.

Kormish, J.D., Sinner, D., and Zorn, A.M. (2010). Interactions between SOX factors and Wnt/β-catenin signaling in development and disease. Dev. Dynam. *239*, 56–68.

Kosar, K., Nejak-Bowen, K., and Li, Q. (2020). Determining the effects of Wnt signaling in the alleviation of cholestasis via the promotion of hepatocyte transdifferentiation. Faseb. J. *34*, 1.

Kumar, R., and Wang, R.-A. (2016). Structure, expression and functions of MTA genes. Gene *582*, 112–121.

Labouesse, M., Hartwieg, E., and Horvitz, H.R. (1996). The Caenorhabditis elegans LIN-26 protein is required to specify and/or maintain all non-neuronal ectodermal cell fates. Development *122*, 2579–2588.

Lambert, J., Lloret-Fernández, C., Laplane, L., Poole, R.J., and Jarriault, S. (2021). On the origins and conceptual frameworks of natural plasticity—lessons from single-cell models in C. elegans. Curr. Top. Dev. Biol. *144*, 111–159.

Landmann, F., Quintin, S., and Labouesse, M. (2004). Multiple regulatory elements with spatially and temporally distinct activities control the expression of the epithelial differentiation gene lin-26 in C. elegans. Dev. Biol. *265*, 478–490.

Lee, J., Taylor, C.A., Barnes, K.M., Shen, A., Stewart, E.V., Chen, A., Xiang, Y.K., Bao, Z., and Shen, K. (2019). A Myt1 family transcription factor defines neuronal fate by repressing non-neuronal genes. Elife *8*, e46703.

Lin, R., Thompson, S., and Priess, J.R. (1995). pop-1 encodes an HMG box protein required for the specification of a mesoderm precursor in early C. elegans embryos. Cell *83*, 599–609.

Lloret-Fernández, C., Maicas, M., Mora-Martínez, C., Artacho, A., Jimeno-Martín, Á., Chirivella, L., Weinberg, P., and Flames, N. (2018). A transcription factor collective defines the HSN serotonergic neuron regulatory landscape. Elife *7*, e32785. https://doi.org/10.7554/eLife.32785.

Lorson, M.A., Horvitz, H.R., and van den Heuvel, S. (2000). LIN-5 is a novel component of the spindle apparatus required for chromosome segregation and cleavage plane specification in Caenorhabditis elegans. J. Cell Biol. *148*, 73–86.

Malik, V., Zimmer, D., and Jauch, R. (2018). Diversity among POU transcription factors in chromatin recognition and cell fate reprogramming. Cell. Mol. Life Sci. *75*, 1587–1612.

Marro, S., Pang, Z.P., Yang, N., Tsai, M.-C., Qu, K., Chang, H.Y., Südhof, T.C., and Wernig, M. (2011). Direct lineage conversion of terminally differentiated hepatocytes to functional neurons. Cell Stem Cell *9*, 374–382.

Marson, A., Foreman, R., Chevalier, B., Bilodeau, S., Kahn, M., Young, R.A., and Jaenisch, R. (2008). Wnt signaling promotes reprogramming of somatic cells to pluripotency. Cell Stem Cell *3*, 132–135.

Maruyama, M., Ichisaka, T., Nakagawa, M., and Yamanaka, S. (2005). Differential roles for Sox15 and Sox2 in transcriptional control in mouse embryonic stem cells. J. Biol. Chem. *280*, 24371–24379.

McIntire, S.L., Jorgensen, E., Kaplan, J., and Horvitz, H.R. (1993). The GABAergic nervous system of Caenorhabditis elegans. Nature *364*, 337–341.

Mello, C.C., Kramer, J.M., Stinchcomb, D., and Ambros, V. (1991). Efficient gene transfer in C.elegans: extrachromosomal maintenance and integration of transforming sequences. EMBO J. *10*, 3959–3970.

CellPress
OPEN ACCESS

Merrell, A.J., and Stanger, B.Z. (2016). Adult cell plasticity in vivo: de-differentiation and transdifferentiation are back in style. Nat. Rev. Mol. Cell Biol. *17*, 413–425.

Merrill, B.J. (2012). Wnt pathway regulation of embryonic stem cell self-renewal. Cold Spring Harbor Perspect. Biol. *4*, a007971.

Miyaoka, Y., and Miyajima, A. (2013). To divide or not to divide: revisiting liver regeneration. Cell Div. *8*, 8.

Miyazaki, K. (2003). Creating random mutagenesis libraries by megaprimer PCR of whole plasmid (MEGAWHOP). Methods Mol. Biol. *231*, 23–28.

Mizumoto, K., and Sawa, H. (2007). Two βs or not two βs: regulation of asymmetric division by β-catenin. Trends Cell Biol. *17*, 465–473.

Molina-García, L., Lloret-Fernández, C., Cook, S.J., Kim, B., Bonnington, R.C., Sammut, M., O'Shea, J.M., Gilbert, S.P., Elliott, D.J., Hall, D.H., et al. (2020). Direct glia-to-neuron transdifferentiation gives rise to a pair of male-specific neurons that ensure nimble male mating. Elife *9*, e48361.

Morin, M.C., Hoff-Yoessle, S., and Jarriault, S. (2020). A simple PCR-based method to follow and genotype alleles with single nucleotide changes. MicroPubl. Biol. https://doi.org/10.17912/micropub.biology.000218.

Mukherjee, S., Chaturvedi, P., Rankin, S.A., Fish, M.B., Wlizla, M., Paraiso, K.D., MacDonald, M., Chen, X., Weirauch, M.T., Blitz, I.L., et al. (2020). Sox17 and β-catenin co-occupy Wnt-responsive enhancers to govern the endoderm gene regulatory network. Elife *9*, e58029.

Narasimhan, K., Lambert, S.A., Yang, A.W., Riddell, J., Mnaimneh, S., Zheng, H., Albu, M., Najafabadi, H.S., Reece-Hoyes, J.S., Fuxman Bass, J.I., et al. (2015). Mapping and analysis of Caenorhabditis elegans transcription factor sequence specificities. Elife *4*, e06967.

Nashun, B., Hill, P.W.S., Smallwood, S.A., Dharmalingam, G., Amouroux, R., Clark, S.J., Sharma, V., Ndjetehe, E., Pelczar, P., Festenstein, R.J., et al. (2015). Continuous histone replacement by hira is essential for normal transcriptional regulation and de novo DNA methylation during mouse oogenesis. Mol. Cell *60*, 611–625.

Ng, H.-H., and Surani, M.A. (2011). The transcriptional and signalling networks of pluripotency. Nat. Cell Biol. *13*, 490–496.

Okada, T.S. (1986). Transdifferentiation in animal models: fact or artifact? Dev. Growth Differ. *28*, 213–221.

Okada, T.S. (1991). Transdifferentiation: Flexibility in Cell Differentiation (Oxford Science Publications - Clarendon Press).

Packer, J.S., Zhu, Q., Huynh, C., Sivaramakrishnan, P., Preston, E., Dueck, H., Stefanik, D., Tan, K., Trapnell, C., Kim, J., et al. (2019). A lineage-resolved molecular atlas of *C. elegans* embryogenesis at single-cell resolution. Science *365*, eaax1971.

Pevny, L.H., and Lovell-Badge, R. (1997). Sox genes find their feet. Curr. Opin. Genet. Dev. *7*, 338–344.

Richard, J.P., Zuryn, S., Fischer, N., Pavet, V., Vaucamps, N., and Jarriault, S. (2011). Direct in vivo cellular reprogramming involves transition through discrete, non-pluripotent steps. Development *138*, 1483–1492.

Rothman, J., and Jarriault, S. (2019). Developmental plasticity and cellular reprogramming in *Caenorhabditis elegans*. Genetics *213*, 723–757.

Sawa, H., and Korswagen, H.C. (2013). Wnt signaling in C. elegans. WormBook, 1–30. https://doi.org/10.1895/wormbook.1.7.2.

Sawa, H., Lobel, L., and Horvitz, H.R. (1996). The Caenorhabditis elegans gene lin-17, which is required for certain asymmetric cell divisions, encodes a putative seven-transmembrane protein similar to the Drosophila frizzled protein. Genes Dev. *10*, 2189–2197.

Schlesinger, A., Shelton, C.A., Maloof, J.N., Meneghini, M., and Bowerman, B. (1999). Wnt pathway components orient a mitotic spindle in the early Caenorhabditis elegans embryo without requiring gene transcription in the responding cell. Genes Dev. *13*, 2028–2038.

Sinner, D., Kordich, J.J., Spence, J.R., Opoka, R., Rankin, S., Lin, S.-C.J., Jonatan, D., Zorn, A.M., and Wells, J.M. (2007). Sox17 and Sox4 differentially regulate beta-catenin/T-cell factor activity and proliferation of colon carcinoma cells. Mol. Cell Biol. *27*, 7802–7815.

Sulston, J.E., and Horvitz, H.R. (1977). Post-embryonic cell lineages of the nematode, Caenorhabditis elegans. Dev. Biol. *56*, 110–156.

Sulston, J.E., Schierenberg, E., White, J.G., and Thomson, J.N. (1983). The embryonic cell lineage of the nematode Caenorhabditis elegans. Dev. Biol. *100*, 64–119.

Takahashi, K., and Yamanaka, S. (2006). Induction of pluripotent stem cells from mouse embryonic and adult fibroblast cultures by defined factors. Cell *126*, 663–676.

Takahashi, K., and Yamanaka, S. (2016). A decade of trancription factor-mediated reprogramming to pluripotency. Nat. Rev. Mol. Cell Biol. *17*, 183–193.

Thorel, F., Népote, V., Avril, I., Kohno, K., Desgraz, R., Chera, S., and Herrera, P.L. (2010). Conversion of adult pancreatic alpha-cells to beta-cells after extreme beta-cell loss. Nature *464*, 1149–1154.

Toker, A.S., Teng, Y., Ferreira, H.B., Emmons, S.W., and Chalfie, M. (2003). The Caenorhabditis elegans spalt-like gene sem-4 restricts touch cell fate by repressing the selector Hox gene egl-5 and the effector gene mec-3. Development *130*, 3831–3840.

Trembley, A. (1744). Mémoires pour servir à l'histoire d'un genre de polypes d'eau douce, à bras en forme de cornes/(Paris, Durand, 1744 and Leyde (Jean & Herman Verbeek).

Treutlein, B., Lee, Q.Y., Camp, J.G., Mall, M., Koh, W., Shariati, S.A.M., Sim, S., Neff, N.F., Skotheim, J.M., Wernig, M., and Quake, S.R. (2016). Dissecting direct reprogramming from fibroblast to neuron using single-cell RNA-seq. Nature *534*, 391–395.

Tsubooka, N., Ichisaka, T., Okita, K., Takahashi, K., Nakagawa, M., and Yamanaka, S. (2009). Roles of Sall4 in the generation of pluripotent stem cells from blastocysts and fibroblasts. Gene Cell. *14*, 683–694.

Valle-Encinas, E., Aleksieva, N., Velasco Martinez, C., Dawes, M., Zverev, M., Müller, M., Offergeld, A., Smalley, M.J., Hogan, C., Forbes, S.J., et al. (2020). Canonical Wnt signalling is activated during BEC-to-hepatocyte conversion in vivo and modulates liver epithelial cell plasticity in hepatic organoids. Preprint at bioRxiv. https://doi.org/10.1101/2020.11.09.374462.

van den Heuvel, S. (2005). Cell-cycle regulation. In WormBook (The C. elegans Research Community, WormBook). https://doi.org/10.1895/wormbook.1.28.1. http://www.wormbook.org.

Vidal, B., Santella, A., Serrano-Saiz, E., Bao, Z., Chuang, C.-F., and Hobert, O. (2015). C. elegans SoxB genes are dispensable for embryonic neurogenesis but required for terminal differentiation of specific neuron types. Development *142*, 2464–2477.

Virchow. (1886). Congrès périodique international des sciences médicales. 8ème session. Compte rendu. Copenhague, 1884. In Congrès Périodique International Des Sciences Médicales (librarie Gyldendal).

Wang, S., Tang, N.H., Lara-Gonzalez, P., Zhao, Z., Cheerambathur, D.K., Prevo, B., Chisholm, A.D., Desai, A., and Oegema, K. (2017). A toolkit for tissue-specific protein degradation in C. elegans. Development *144*, 2694–2701.

White, J.G., Southgate, E., Thomson, J.N., and Brenner, S. (1986). The structure of the nervous system of the nematode Caenorhabditis elegans. Philos. Trans. R. Soc. Lond. B Biol. Sci. *314*, 1–340.

Wildwater, M., Sander, N., de Vreede, G., and van den Heuvel, S. (2011). Cell shape and Wnt signaling redundantly control the division axis of C. elegans epithelial stem cells. Development *138*, 4375–4385.

Xu, J., Du, Y., and Deng, H. (2015). Direct lineage reprogramming: strategies, mechanisms, and applications. Cell Stem Cell *16*, 119–134.

Yamamoto, Y., Takeshita, H., and Sawa, H. (2011). Multiple Wnts redundantly control polarity orientation in Caenorhabditis elegans epithelial stem cells. PLoS Genet. *7*, e1002308.

Yanger, K., Zong, Y., Maggs, L.R., Shapira, S.N., Maddipati, R., Aiello, N.M., Thung, S.N., Wells, R.G., Greenbaum, L.E., and Stanger, B.Z. (2013). Robust

cellular reprogramming occurs spontaneously during liver regeneration. Genes Dev. *27*, 719–724.

Zuryn, S., Le Gras, S., Jamet, K., and Jarriault, S. (2010). A strategy for direct mapping and identification of mutations by whole-genome sequencing. Genetics *186*, 427–430.

Zuryn, S., Ahier, A., Portoso, M., White, E.R., Morin, M.-C., Margueron, R., and Jarriault, S. (2014). Sequential histone-modifying activities determine the robustness of transdifferentiation. Science *345*, 826–829.

Altun, Z.F. and Hall, D.H. 2009. Alimentary system, rectum and anus. InWormAtlas. doi:10.3908/wormatlas.1.5. Edited for the web by Laura A. Herndon. Last revision: April 23, 2013.

STAR★METHODS

KEY RESOURCES TABLE

REAGENT or RESOURCE	SOURCE	IDENTIFIER
Antibodies		
HA Tag Polyclonal Antibody	IGBMC antibody Facility	Rabbit Polyclonal
FLAG Tag Monoclonal Antibody	IGBMC antibody Facility	Mouse Monoclonal
Bacterial and virus strains		
Escherichia coli: OP50	Caenorhabditis Genetics Center	OP50
Escherichia coli: HT115(DE3)	Caenorhabditis Genetics Center	HT115(DE3)
Escherichia coli: DH10β	Invitrogen™	DH10β
Escherichia coli: BL21(DE3)	Stratagene	BL21(DE3)
Chemicals, peptides, and recombinant proteins		
Proteinase K from *Tricachium album*	Sigma-Aldrich	Cat#: P2308
Ampicillin	Sigma-Aldrich	N/A
IPTG	GoldBio	Cat#: I2481C
KH2PO4	Sigma-Aldrich	Cat#: P0662
Na2HPO4	Thermo Fisher Scientific	Cat#: S374-3
NaCl	Thermo Fisher Scientific	Cat#: S271-3
MgSO4	Sigma-Aldrich	Cat#: M7506
cOmplete Mini, EDTA-free Protease Inhibitor Cocktail	Millipore Sigma	Cat#: 11836170001
Tetramisole hydrochloride	Sigma-Aldrich	Cat#: L9756-5G
Tricaine	Sigma-Aldrich	Cat#: E10521-10G
NaOH	Sigma-Aldrich	
NaCl	Sigma-Aldrich	
Tris pH8	Sigma-Aldrich	
EDTA	Sigma-Aldrich	
Dithiothreitol (DTT)	ThermoFisher Scientific	Cat#: R0861
Critical commercial assays		
Taq DNA Polymerase, 5 U/μL	Roche	11 435 094 001
Master Mix Phusion	New England Biolabs	M0531L
Q5® Site-Directed Mutagenesis Kit	New England Biolabs	E0554S
T4 DNA Ligase	New England Biolabs	M0202
mMESSAGE mMACHINE™ T7 Transcription Kit	Invitrogen™	AM1344
NucleoSpin® Gel and PCR Clean-up	Macherey-Nagel	740609.50
NucleoSpin® Plasmid	Macherey-Nagel	740588.50
NucleoBond® Xtra Midi	Macherey-Nagel	740410.50
Hitrap Ni crude 1 mL columns	Dutscher	17-5247-01
NGM agar plates	N/A	N/A
LB agar plates	N/A	N/A
LB	N/A	N/A
Experimental models: Organisms/strains		
C. elegans strains: See Table S2		
Oligonucleotides		
Oligonucleotides: See Table S3	Sigma-Aldrich	

(*Continued on next page*)

Continued		
REAGENT or RESOURCE	SOURCE	IDENTIFIER
Recombinant DNA		
CloneJET PCR Cloning Kit	Thermo Fisher Scientific	K1231
pET30a+	Novagen	pET30a+
pET32a+	Novagen	pET32a+
pPD95.75	Fire Kit, Addgene	pPD95.75
pPD97.82	Fire Kit, Addgene	pPD97.82
pPD122.53	Fire Kit, Addgene	pPD122.53
pOD1988	Wang et al., 2017	pOD1988
2nls::gfp	This study	pSJ553
egl-5p(6.5 kb)::nanobodyGFP::zif-1.	This study	pSJ559
lin-17p::2nls::gfp.	This study	pSJ567
lim-6(int4)::gfp	This study	pSJ739
lim-6int4(mutated)::gfp.	This study	pSJ759
let-413a::gfp::pest	This study	pSJ721.14
lin-26p::gfp	This study	pSJ722
T7p::6XHis::hmg-pop-1	This study	pSJ769
T7p::6XHis::sox-2 full::HA	This study	pSJ6094
T7p::6XHis::hmg-pop-1::Flag	This study	pSJ1107
col-34p::gfp::cki-1(gDNA)::unc-54 3′utr	This study	pSJ1108
col-34p::gfp::cki-1(cDNA)::unc-54 3′utr	This study	pSJ1112
egl-5p(1,3 kb)::sox-2 full antisense.	This study	pSJ6293
lim-6(int4)::mCherry	This study	pSJ1096
col-34p::gfp::sox-2(cDNA)::unc-54 3′utr	This study	pSJ6199
Software and algorithms		
Adobe Illustrator CS6	http://www.adobe.com/products/illustrator.html	RRID:SCR_010279
Fiji	https://imagej.net/Fiji/Downloads	RRID: SCR_002285
Imaris	https://imaris.oxinst.com/packages	RRID: SCR_007370
GraphPad Prism	https://www.graphpad.com/	RRID: SCR_002798
LAS X	https://www.leica-microsystems.com/products/microscope-software/p/leica-las-x-ls/	RRID:SCR_013673
SnapGene viewer	http://www.snapgene.com/products/snapgene_viewer/	RRID:SCR_015053
ApE (A plasmid Editor)	https://jorgensen.biology.utah.edu/wayned/ape/	N.A
UCSC Genome Browser	http://genome.ucsc.edu/cgi-bin/hgTracks?db=ce11&lastVirtModeType=default&lastVirtModeExtraState=&virtModeType=default&virtMode=0&nonVirtPosition=&position=chrX%3A1074694%2D1078639&hgsid=1004849487_ACKae4H3vpcGQxQU9xfEluAVn6Sj	RRID:SCR_005780
Genomatix Software: MatInspector	https://www.genomatix.de/online_help/help_matinspector/matinspector_help.html	RRID:SCR_008036
ALGEN-PROMO	http://alggen.lsi.upc.es/cgi-bin/promo_v3/promo/promoinit.cgi?dirDB=TF_8.3	RRID:SCR_016926
FIMO	http://meme-suite.org/doc/fimo.html	RRID:SCR_001783
Jaspar	http://jaspar.genereg.net/	RRID:SCR_003030
CIS-BP	http://cisbp.ccbr.utoronto.ca/TFreport.php?searchTF=T333272_2.00	RRID:SCR_017236

(*Continued on next page*)

Continued

REAGENT or RESOURCE	SOURCE	IDENTIFIER
Other		
Leica MZ6	Leica Microsystems	
Leica SP5	Leica Microsystems	
Leica DM6 B	Leica Microsystems	
HAMAMATSU Digital Camera		C11440-42U30

RESOURCE AVAILABILITY

Lead contact

Further information and requests for resources and reagents should be directed to and will be fulfilled by the lead contact, Sophie Jarriault (sophie@igbmc.fr).

Materials availability

Reagents generated in this study will be made available on request, but we may require a payment and/or a completed Materials Transfer Agreement if there is potential for commercial application.

Data and code availability

- All data reported in this paper will be shared by the lead contact upon request.
- This paper does not report original code.
- Any additional information required to reanalyze the data reported in this paper is available from the lead contact upon request.

EXPERIMENTAL MODEL AND SUBJECT DETAILS

C. elegans strains were maintained on agar plates containing NGM growth media seeded *with E. coli* strain OP50 (Brenner, 1974) at 20°C, except for temperature-sensitive strains *(lin-5(ev571 ts), wrm-1(ne1982 ts), lin-18(n1051 ts), par-1(zu310 ts))* which were grown at 15°C. To score the (hermaphrodite) larvae mutant phenotype in temperature-sensitive strains, an egg pulse population (spanning over 1 h) was shifted at the restrictive temperature (25°C) to avoid early embryonic lethality and scored in L2. The strains used in this study are summarized in Table S2.

Construction of *C. elegans* strains

C. elegans transgenic strains were created by DNA microinjection in the gonad of young adult hermaphrodites (Mello et al., 1991) of the plasmid of interest together with a co-injection marker and pBSK to a final concentration of DNA of 200–250 ng/μL in water. For the SOX-2 overexpression experiments, a *lim-6int4::mCherry::unc-54 3′utr* fragment was amplified using custom oligo oCG-444F and oligo D(r) using pSJ1096 as a template. Fragment *col-34p::gfp::sox-2(cDNA)::unc-54 3′utr* was PCR amplified by custom oligo BDN519 and oligoD using pSJ6199 as a template. All PCR were carried out by Hi-Fidelity Phusion polymerase following standard PCR method. Amplicons were purified (NucleoSpin® Gel and PCR Clean-up) and quantified (NanoDrop 1000 Spectrophotometer) and injected into the gonads of young hermaphrodite animals [*lim-6int4::mCherry::unc-54 3′utr(*20 ng/μL), *myo-2p::GFP*(2 ng/μL) & *pBSK*+(250 ng/μL) ± *col-34p::gfp::sox-2(cDNA)::unc-54 3′utr(*25 ng/μL)].

PCR fragments injections: *col-34p::gfp::cki-1(gDNA)::unc-54 3′utr* and *col-34p::gfp::cki-1(cDNA)::unc-54 3′utr* fragments were amplified by custom oligo BDN519 and oligoD with template pSJ1108 and pSJ1112 respectively. Injection mix was composed of *col-34p::gfp::cki-1(gDNA)::unc-54 3′utr* or *col-34p::gfp::cki-1(cDNA)::unc-54 3′utr (20 ng/μL)*, *pCFJ90* (2 ng/μL) & *pBSK*+(250 ng/μL) and injected into gonad of young adult hermaphrodite animals.

All the other strains used in this work (Table S2) were obtained by crossing existing strains from our lab, other labs (obtained through CGC or directly) or obtained from SunyBiotech for knock-in reporter strains. The presence of mutant alleles was confirmed by PCR genotyping in case of deletions and by PCR + restriction genotyping in case of point mutations (Morin et al., 2020). Primers used for genotyping are summarized in Table S3.

Plasmid construction

pSJ553 – *2nls::gfp*. The 2NLS sequence was amplified by PCR from pSJ207 (Kagias et al., 2012) with primers oCG390/oCG391 and cloned KpnI/XhoI into pPD95.75.

pSJ559 – *egl-5p(6.5 kb)::nanobodyGFP::zif-1. nanobodyGFP::zif-1::U54 3′UTR* was amplified by PCR from pOD1988 plasmid (Wang et al., 2017) with primers oCR073/oCR074 and cloned into pSJ671 AscI/ApaI sites, containing *egl-5p::Δpes10*.

pSJ567 – *lin-17p::2nls::gfp*. *lin-17p* (6.5 kb) was amplified by PCR from genomic DNA with primers oCR155/oCR156 and cloned into pSJ553 HindIII/PstI sites.

pSJ721.14 – *let-413::gfp::pest*. The *Mus musculus* ornithine decarboxylase PEST sequence (Corish and Tyler-Smith, 1999) was inserted by Megawhop cloning (Miyazaki, 2003) into pML801 plasmid (a gift from Michel Labouesse). The *pest* sequence (120 bp) was obtained through annealing of 2 μg of oligonucleotides oCG368 and oCG369 in 10 mM Tris pH8, 50 mM NaCl and 1 mM EDTA. The annealing was performed in 50μL for 5′ at 95°C and then ramping down to about 25°C with a rate of −1.5 °C/min. After the annealing, the *pest* sequence was cloned into pJET1.2/blunt (Thermo Fisher Scientific) and subsequently amplified with primers oCG370/oCG371 and cloned into pML801 by Megawhop cloning.

pSJ722 – *lin-26rectalp::nls::gfp*. The rectal specific promoter of *lin-26* (Landmann et al., 2004) was PCR-amplified from genomic DNA with primers oCG381/oCG382 and cloned by Megawhop cloning into pPD97.82.

pSJ739 – *lim-6int4::gfp*. *lim-6 intron 4* was PCR-amplified from genomic DNA with primers oCG444/oCG445 and cloned by Megawhop cloning into pPD95.75.

pSJ759 – *lim-6int4(mutated)::gfp*. The sequence of the *lim-6 intron 4* with 7 out of 8 mutated TCF binding sites (Figure S9) was ordered (ProteoGenix, France) flanked with BbsI and SalI restriction sites. This allowed it to be cloned into pSJ739, replacing the wild-type *lim-6int4* sequence.

pSJ769 – *T7p::6XHis::hmg-pop-1*. The sequence of the HMG domain of *pop-1* was PCR-amplified from genomic DNA with primers oCG556/oCG557 and cloned by Megawhop cloning into pET30a+.

pSJ6094 - *T7p::6XHis::sox-2 full::HA*. The *sox-2::HA* sequence was PCR-amplified from peYFP-sox-2-HA (Kagias et al., 2012) with primers psj6094sox-2 F/R and cloned into pET32a+ SalI/Not1 sites.

pSJ6293 - *egl-5p(1,3 kb)::sox-2 full antisense. egl-5p(1,3 kb)* was PCR amplified with primers pLG7F/pLG7R and cloned into pPD122.53 at the SalI/XbaI sites. The full *sox-2* antisense cDNA was PCR amplified with primers BDT950/952 from a mRNA prep and cloned by Megawhop to replace the GFP present in the original L4053 plasmid.

pSJ1096 - *lim-6int4::mCherry::unc-54 3′utr.* GFP was removed from pSJ739 using *KpnI* and *EcoRI* restriction sites then digested fragment was assembled with overlapping ends containing mCherry fragment (obtained using oligos oSKS-212 and oSKS-213 via standard PCR method) using HiFi DNA assembly kit (New England Biolabs), following the manufacture's protocol.

The coding sequences in all constructs were verified by Sanger sequencing. All plasmids were transformed into DH10β bacteria (Invitrogen™) for DNA amplification or BL21(DE3) (Stratagene) for protein production

pSJ1107 - *T7p::6XHis::hmg-pop-1::Flag*. Flag tag was inserted at the C-terminus of *hmg-pop-1* in pSJ769 using Q5® Site-Directed Mutagenesis Kit (New England Biolabs), following the manufacturer's protocol. Oligo oSKS-235 & oSKS-236 were used for this modification and their sequence is listed in Table S3.

pSJ1108 - *col-34p::gfp::cki-1(gDNA)::unc-54 3′utr*. *sox-2* was removed from pSJ6199 using PstI and NsiI restriction sites; digested vector was then ligated with *cki-1* fragment (obtained with custom made oligos oSKS-233 & oSKS-234 via standard PCR method & PstI and NsiI digestion) using T4 DNA Ligase (New England Biolabs), following the manufacturer's protocol.

pSJ1112 - *col-34p::gfp::cki-1(cDNA)::unc-54 3′utr*. The intron was removed from pSJ1108 using custom-made oligos (oSKS-237 & oSKS-238; see Table S3) following Q5® Site-Directed Mutagenesis Kit (New England Biolabs) manufacturer's protocol.

METHOD DETAILS

RNAi/silencing experiments

RNAi experiments were performed as previously described (Zuryn et al., 2014). Basically, we sequence-verified clones from the Ahringer library (Kamath et al., 2003). RNAi was performed by injecting double stranded RNA (dsRNA) directly into L4 worms, which we found more effective to suppress gene expression in rectal cells when compared to the feeding method. To this end, the insert of each clone was PCR amplified using T7 primers. I*n vitro* transcription was performed using the PCR products as templates with T7 RNA polymerase using the mMESSAGE mMACHINE™ T7 Transcription Kit (Invitrogen™). Single stranded RNA was allowed to anneal to form dsRNA by gradually lowering the temperature of the sample from 65°C. RNAi sensitized *rrf-3(pk1426) II ; oxIs12[unc-47::gfp]* adults were microinjected with dsRNA and F1 progeny derived from these adults were scored for the presence of DVB. Note that many of the knockdowns tested resulted in significant lethality and that only escapers were scored, possibly biasing the results toward less defects. For *sox-2* rectal-specific knockdown in particular, we used either the rectal expression (under *egl-5(1.3 kb)* promoter) of a *sox-2* antisense sequence, or the rectal expression (under *egl-5*(6 kb) promoter) of the nanobody-GFP system (Wang et al., 2017) in a *gfp::sox-2* CRISPR KI strain. This latter strategy allowed us to monitor GFP::SOX-2 switch-off in parallel to DVB defects.

Epifluorescence microscopy

Worms were immobilized on 2% agarose pads using Tricaine 0.4% and Tetramizole 0.04%. Images were captured on a Leica DM6 B microscope with LAS X software and the HAMAMATSU Digital Camera C11440. For all images, anterior is to the left and dorsal up.

Confocal/spinning disk microscopy

Worms were immobilized on 5% agarose pads with Tricaine 0.4% and Tetramizole 0.04%. For Confocal microscopy, images were captured on an inverted Leica TCS SP5 laser scanning confocal microscope (Leica Microsystems, Germany). For Spinning Disk

microscopy, time-lapse images were captures with an inverted Nikon Eclipse Ti equipped with the PFS (perfect focus system) and a Yokogawa CSU-X1 scan head and a 60×1.4 NA objective lens, and run using Metamorph. For the smFISH, the same microscope was used but with a 100×1.4 NA objective. For all images, anterior is left and dorsal up.

Scoring criteria

Both localization, DIC appearance and fluorescent markers, using in particular the rectal reporter array *gaIs245[col-34p::his-24::mcherry; unc-119(+)]* (Kagias et al., 2012) were used to identify K, K' and K.p cells' nuclei. Of note, K is found on the left side, K' on the right side of the worm, and K.p is posterior to K.a and K'. We further confirmed the left position of K relative to the localization of the commissures of the GABAergic neurons visualized with *oxIs12 [unc-47p::GFP]* and DNA replication was visualized using *gaIs245 [col-34p::his-24::mcherry; unc-119(+)]*. The assessment of K division in *lin-5* mutant was performed by using *gaIs245* together with a fluorescent marker of the plasma membrane of rectal cells (*fpIs101[col-34p::ph::gfp; odr-1p::dsRed]*). In *egl-5(n945)* mutant, since *col-34* expression is absent in rectal cells, an *egl-5* reporter *(bxIs7[egl-5(6.5 kb)::gfp; lin-15(+)])* was used to identify K.a and K.p. This cytoplasmic reporter did not allow us to estimate the orientation of K division based on K.a and K.p nuclei alignment or to measure their nuclear volumes. Similarly, the combination of markers that we used to simultaneously assess K division and DVB formation (*gaIs245* and *wyIs75* for *sox-2*), as well as the overall perturbed rectal area, precluded the quantification of the orientation of K division or the nuclear volumes in *sox-2* knockdown and *ceh-6* mutant. Since the rectal-specific *sox-2* antisense array contains also *rol-6(su1006)*, making it difficult to identify K.a, K′ and K.p in roller mutants, K cell division was scored in animals where *sox-2 is* knocked down by an anti-GFP nanobody strategy (See RNAi/Silencing experiments). This strategy was also used in combination with *wrm-1* mutant to test the genetic interaction between *sox-2* and the Wnt signaling pathway. DVB presence was always based on the expression of *unc-47* terminal differentiation gene (*oxIs12*, *krIs6* or *wyIs75* arrays) and the presence of its stereotyped neurite going anteroventrally. For timing experiments, tightly synchronized worms were obtained via hatch-pulses: more than 100–200 eggs were picked on fresh plates seeded with OP50 and each hour newly hatched larvae were transferred on new plates. Alternatively, L1-L2 worms were picked and staged according to the number of cells in the developing gonad. To determine the precise timing of K division and DVB formation, hatch-pulse tightly synchronized L1 were mounted every hour starting 10 h after hatching. Several criteria were used to precisely stage the worms: the occurrence of division, using *gaIs245*; the L1-L2 transition, using the number of cells in the gonad and the disappearance of the alae observed by DIC. The early L2 stage was further dissected using the number of GABAergic cell bodies in the ventral nerve cord and the number of VD commissures as well as *unc-47* expression in DVB as observed with *oxIs12*.

Electron microscopy

See description in (Jarriault et al., 2008) (SI text). Ten microns of serial ultrathin sections (50–70 nm) of the rectal area were collected and contrasted in lead citrate and uranyl acetate before imaging with a SiS Megaview 3 CCD camera mounted on a FEI Morgagni TEM operated at 70 kV.

Image processing and analysis

Images were processed using ImageJ. The measurement of the angle of K division was performed using the angle tool from ImageJ where a segmented line was drawn through the rectal slit and the center of the K.a and K.p nuclei in late L1 larvae as represented by the yellow dashed line in Figure 2A.

smFISH

The smFISH probe for *lin-26* was designed using the stellaris-designer from biosearchtech web site (https://www.biosearchtech.com/stellaris-designer) and coupled to Quasar 670.

smFISH was performed on the strain IS3423 *gaIs245[col-34p::his-24::mCherry; unc-119(+)] V; sox-2(syb737[GFP::linker::sox-2])X*. *gaIs245* allows the identification of rectal cells and GFP::SOX-2 highlights K.p nucleus after division. Young mothers were bleached and eggs were grown for 26hrs on NGM plate at 20°C. The next day, the L1 larvae were treated as described in Ji and van Oudenaarden (2012). Briefly, larvae were collected and rinsed in M9 twice and fixed for 45 min in 4% paraformaldehyde. Then larvae were rinsed in PBS and incubated in 70% EtOH for at least 20hrs. The next day, larvae were rinsed twice in wash buffer containing 10% formaldehyde and incubated O/N at 30°C protected from light, with 100μL of hybridization buffer containing 1μL of the *lin-26* probe (final concentration 0.6μM). The next day, larvae were rinsed twice in wash buffer and incubated for 30 min with Dapi in wash buffer. Then larvae were rinsed in 2X SSC and mounted on a pad with Vectashield. Images were acquired on a Nikon Spinning Disk microscope as follows: Laser 635 16%, 600 ms exposure, Laser 405 30% 150 ms exposure, Laser 491 30% 200 ms exposure, Laser 561 9% 200 ms exposure, 14 z steps with 0.3 μm between each step.

Quantifications were performed manually as follows: For each cell of interest, we counted the number of spots touching the nucleus, only taking into account cells for which the total volume of the nucleus was visible as determined with our different markers (*his-24::mCherry, gfp::sox-2* and Dapi). We reasoned that spots touching the nucleus ("touching spots") would reflect active transcription.

Transcription factor binding sites analysis

For the manual identification of the putative TF binding sites, the following consensus sequences in Snapgene were used:

POP-1/TCF: WWCAAAR (Bertrand and Hobert, 2009), SEM-4/SPALT: WARATTGTSTKKSW (Lloret-Fernández et al., 2018) and TTGTST (Toker, 2003), SOX-2/SOX2: VACAAWGG (Maruyama et al., 2005).

For automatic identification of putative binding sites, several platforms were used: FIMO with the *C. elegans* POP-1/TCF Matrix (Narasimhan et al., 2015), or MatInspector and Promo from vertebrate homologues.

Conservation of the binding sites was examined using the multiple alignment provided by the UCSC genome Browser. However, very poor conservation of the binding sites was observed among *C. elegans* species.

6×His-tagged protein expression and purification

To make 6×His-tagged proteins*, sox-2, pop-1* and *HMG-pop-1* cDNAs were cloned into pET32a+ or pET30a+ (Addgene) and transformed into BL21(DE3) from Stratagene. 6×His-tagged protein expression was induced by adding 1 mM IPTG to 1L of transformed cell culture at OD600 = 0.6 for 4 h at 37°C. Cells were then harvested and lysed in 40 mL of Lysis Buffer (20 mM Tris pH 8, 100 mM NaCl, 10 mM imidazole, 1 mM DTT, 0.1% NP40, Protease inhibitor from Roche) by sonication. The lysates were centrifuged 30 min at 40 000 RPM, 4°C. 6×His-tagged proteins were purified using a Hitrap Ni crude 1 mL columns (Dutscher) and the following buffers: Equilibration Buffer (20 mM Tris pH 8, 100 mM NaCl, 10 mM imidazole, 0.1% NP40) and Elution Buffer (Equilibration Buffer +300 mM imidazole). The extracts were fractionated on a cation exchange chromatography and then dialyzed in EMSA buffer (20 mM Tris pH 8, 100 mM NaCl, 1 mM EDTA, 1 mM DTT, 0.1% NP40). Note that for unknown reasons, we could not manage to purify the full-length POP-1 protein despite numerous attempts.

Electrophoretic mobility shift assay (EMSA) and super shift assay

Cy5-labeled probes were ordered (Merck, Germany) and annealed as follows: 10μM of forward (CY5) and reverse (unlabeled) primers were incubated in the dark in TE buffer containing 125 mM NaCl at 100°C for 5 min followed by 5hrs at room temperature and then O/N at 4°C. Purified 6×His-tagged proteins (250 mM, or a range of 0,5 to 500 nM) and 200 ng of Cy5-probes were incubated on ice in binding buffer (20 mM Tris HCl pH 8.0, 100 mM NaCl, 100 μg/mL BSA, 1 mM EDTA, 1 mM DTT, 0.1% NP-40) for 20 min and resolved in a pre-run 6% polyacrylamide gel containing 0.5× TBE buffer (Bio-Rad Mini-PROTEAN) at 100 V for 1 h. The gels were imaged using the Typhoon^{TM} FLA 9500 biomolecular imager.

For the super shift assay, recombinant purified HMG-POP-1-FLAG (300 nM) or SOX-2-HA (200 nM) and 200 ng of double stranded annealed Cy5-probe 1 or 2 were incubated at room temperature in binding buffer (20 mM Tris HCl pH 8.0, 100 mM NaCl, 100 μg/mL BSA, 1 mM EDTA, 1 mM DTT, 0.1% NP-40) for 30 min. Then, 2 μM of rabbit polyclonal anti-HA and/or mouse monoclonal anti-FLAG (IGBMC antibody facility) were added to the samples and incubated for 15 min before loading on a 8% non-denaturing polyacrylamide mini gel using running 0.5× TBE buffer (Bio-Rad Mini-PROTEAN) at constant 80 V. The gels were scanned using the Typhoon^{TM} FLA 9500 biomolecular imager (GE healthcare). All the steps involving labelled probe were carried out either in the dark or minimal light condition.

QUANTIFICATION AND STATISTICAL ANALYSIS

Statistical analysis and data representations

In the bar plots, mean and standard deviation between biological replicates of the percentage of worms scored are represented. The stars summarize the statistical significance as calculated through Fisher's exact test on the merged raw data from single replicates in a contingency table. Two-tailed Fisher's exact test was used to compare reporter gene expression in mutant vs wild-type worms. One-tailed Fisher's test was used to compare Td defects in mutant vs wild-type worms; the choice of the one-tailed test is justified by the known 0% Td defect in wild-type worms. The Student's t-test was used to analyze the significance in the difference between K.a and K.p nuclear volumes' ratio and the angle of K division in wild-type vs *lin-17* and *sem-4* mutants. F test was used to compare the variances of the angle of division between wild-type vs *lin-17*, *sem-4* and *goa-1* mutants. For the smFISH, an Anova and Dunnet T3 test for multiple comparison was performed. When relevant, top bars represent mean + standard deviation. *$p < 0.05$; **$p < 0.01$; ***$p < 0.001$; ****$p < 0.0001$ and ns, not significant, for all the tests.

Quantification of nuclear volumes

To measure K.a, K.p and K′ nuclear volumes, *gals245* transgenic strains were imaged at Leica SP5 confocal microscope. All the volume in Z containing K.a, K.p and K′ nuclei was acquired with a z-step size of 0.3 μm. Imaris software was used to reconstruct 3D images and to analyze the volume occupied by each nucleus. Manual selection of the nuclear area was performed.

Cell Reports, Volume *40*

Supplemental information

A natural transdifferentiation event involving mitosis is empowered by integrating signaling inputs with conserved plasticity factors

Claudia Riva, Martina Hajduskova, Christelle Gally, Shashi Kumar Suman, Arnaud Ahier, and Sophie Jarriault

Fig. S1.

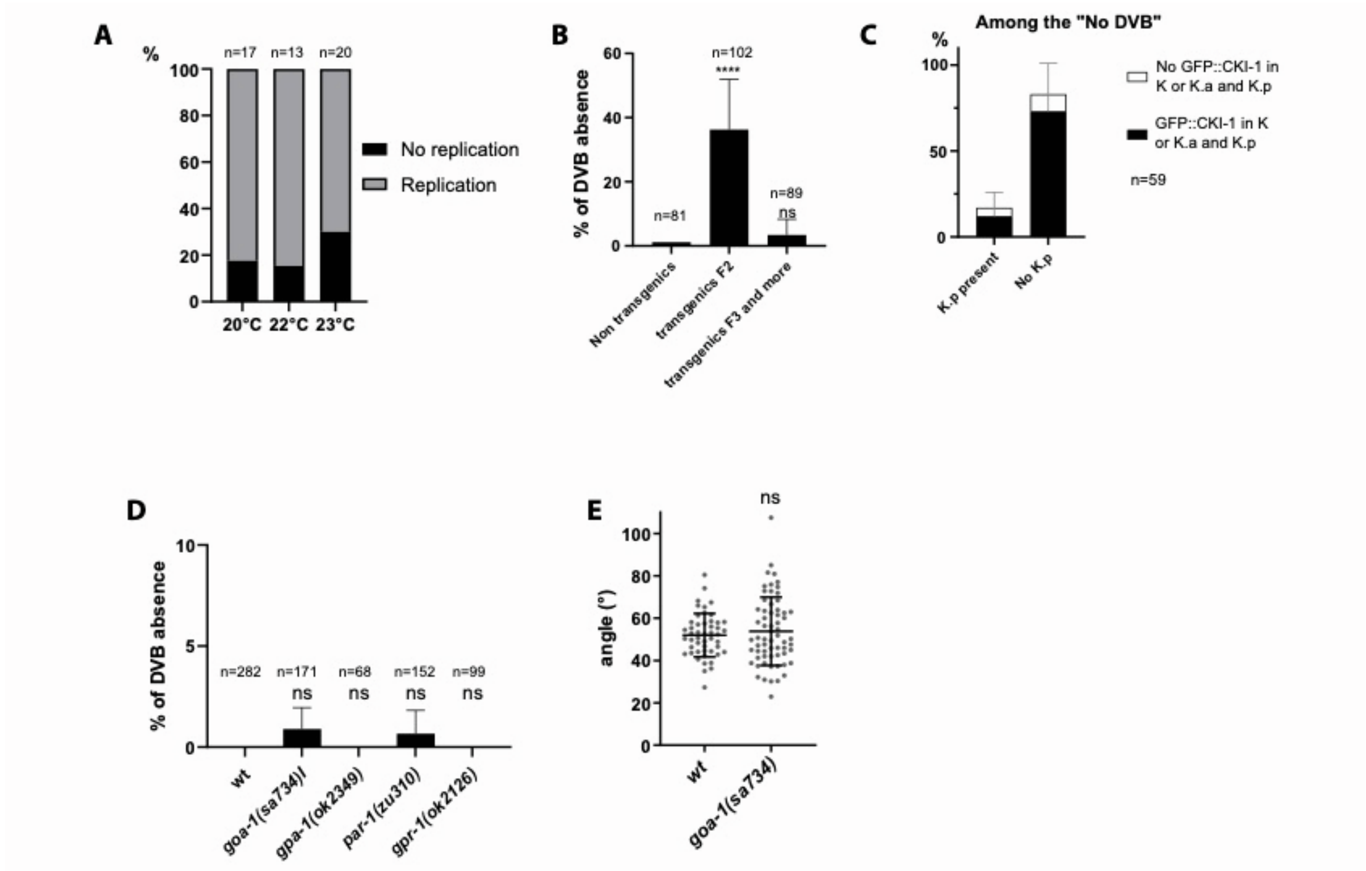

Figure S1. While DNA replication in K is not sufficient for DVB formation and division orientation mutants do not impact K-to-DVB, K division is required for K-to-DVB. Related to Figure 2.

(A) Histogram summarizing the percentage of animals in which K DNA underwent replication in the *lin-5(ev571ts)* mutant at different restrictive temperatures.

(B) Histogram showing the percentage of animals with a "NO DVB" defect, and without (Non transgenics) or with (transgenics) overexpression of *gfp::cki-1* in the rectal cells. Note that for the transgenics, the F2 generation of the transgenic lines was first scored and displayed more penetrant defects than the following generations, due to transgene silencing.

(C) Histogram showing the percentage animals displaying an absence (No K.p) or presence (K.p present) of the K.p cell among the *gfp::cki-1* overexpressing animals without DVB. Note that for each category, the GFP expression was assessed in K, K.a and K.p, when present. Solid bar: GFP expression was observed; white bar, No GFP expression. Error bars, SD of the GFP-expressing animals.

(D) Histogram showing the percentage of worms without DVB in mutants for the Gα and *gpr-1/LGN* genes involved in spindle orientation in *C. elegans* zygote and for the Par gene *par-1*. The low penetrance of DVB absence is due to an impairment in K cytokinesis.

(E) Dot plot representing K division angle in the *goa-1(sa734)* mutant. n=64.

For all the histograms, ns, not significant; n, total animal scored.

Fig. S2.

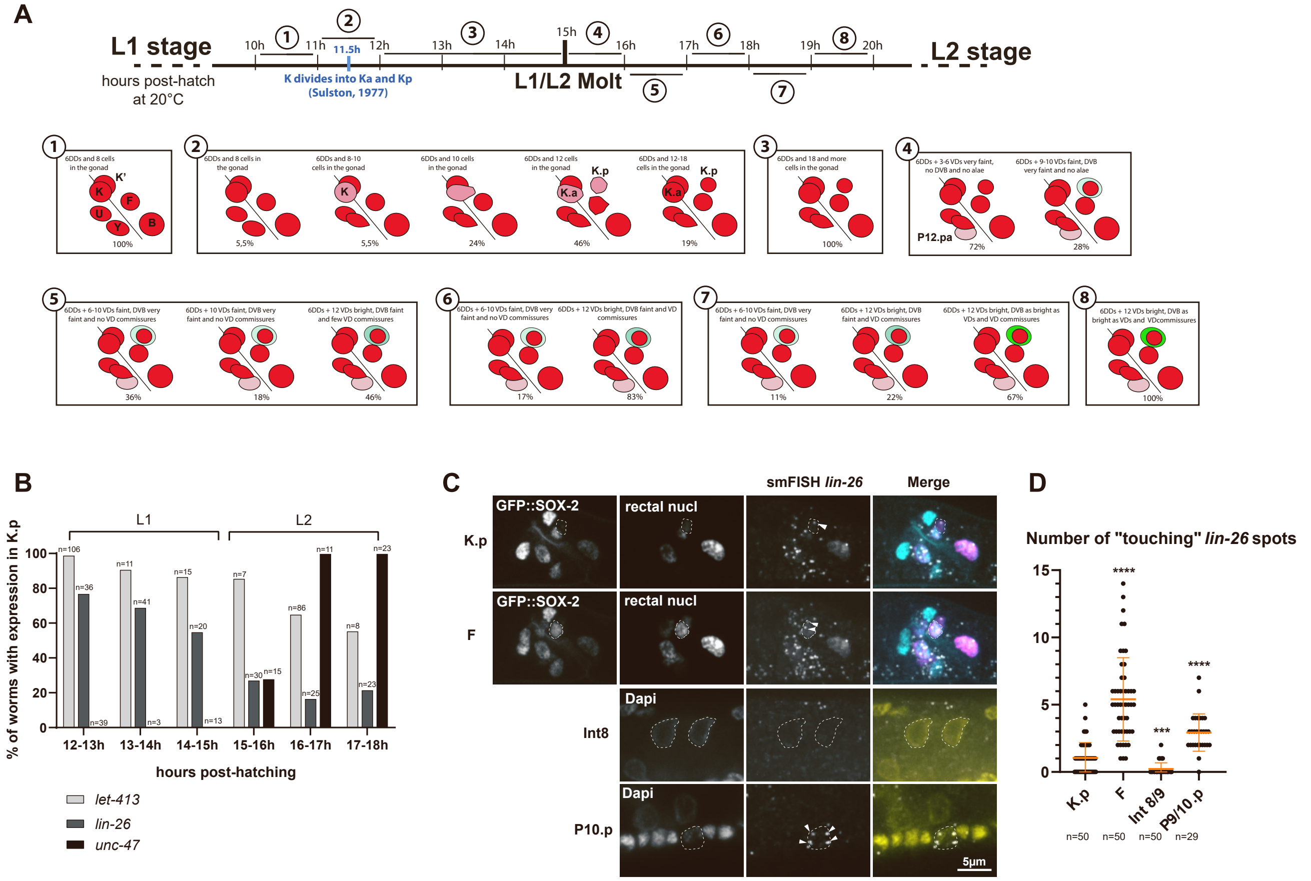

Figure S2. K divides into K.a and K.p in late L1 stage and K.p still shows epithelial features after division. Related to Figure 2.

(A) Time course of the cellular events during K division and DVB formation along the developmental timeline. The *col-34p::mCherry* positive rectal cells and the rectal slit (line) are represented. Each box corresponds to a point of one hour where different characteristic landmarks have been observed in addition to K division: number of GABAergic neurons, VD commissures, presence of alae, number of cells in the gonad, *col-34p::mCherry* and *unc-47p::GFP* intensities, rectal cell shapes. The percentage of animals (n%) with the corresponding landmarks is indicated at each particular time point. Worms were synchronized by hatch pulse (see Methods). Left is towards front, anterior is left, and dorsal is up.

(B) Quantification of worms with expression of *fpEx1062[let-413::gfp::pest*], *fpIs110[lin-26p::GFP]* and *oxIs12[unc-47p::GFP]* in K.p over time, in L1 and L2 grown at 20°C. n, total animal scored.

(C) *lin-26* smFISH staining (white spots on the merge) on 26h post hatching L1 larvae expressing a *gfp::sox-2* (CRISPR KI *syb737*, shown in cyan) and the rectal nuclear reporter *gaIs245*, shown in magenta. Intestinal cells and Pn.p cells in the same area were identified with the DAPI staining, in yellow. Scale bar for all in bottom right picture.

(D) Quantifications of the *lin-26* smFISH spots were performed on K.p and F rectal cells as well as on intestinal cells (Int 8/9) and Pn.p cells (P9/10.p). We considered only the spots in contact with the nuclei to reflect the most recently transcribed mRNAs. Statistical test compares the number of spots in the K.p cell to the others. Very few *lin-26* mRNA molecules are detected in close proximity to the nucleus in epithelial cells generally (from 3 spots to 5 spots on average; P9/10.p where *lin-26* has been shown to be expressed (Labouesse et al., 1996), 3 spots; rectal F cell, 5 spots; K.a, which is on the left side, was very difficult to image because of photobleaching (n=9) and exhibits 4 spots on average). Intestinal cells, where *lin-26* is not expressed, show no *lin-26* mRNA spots (0, 22 spots on average).

Fig. S3.

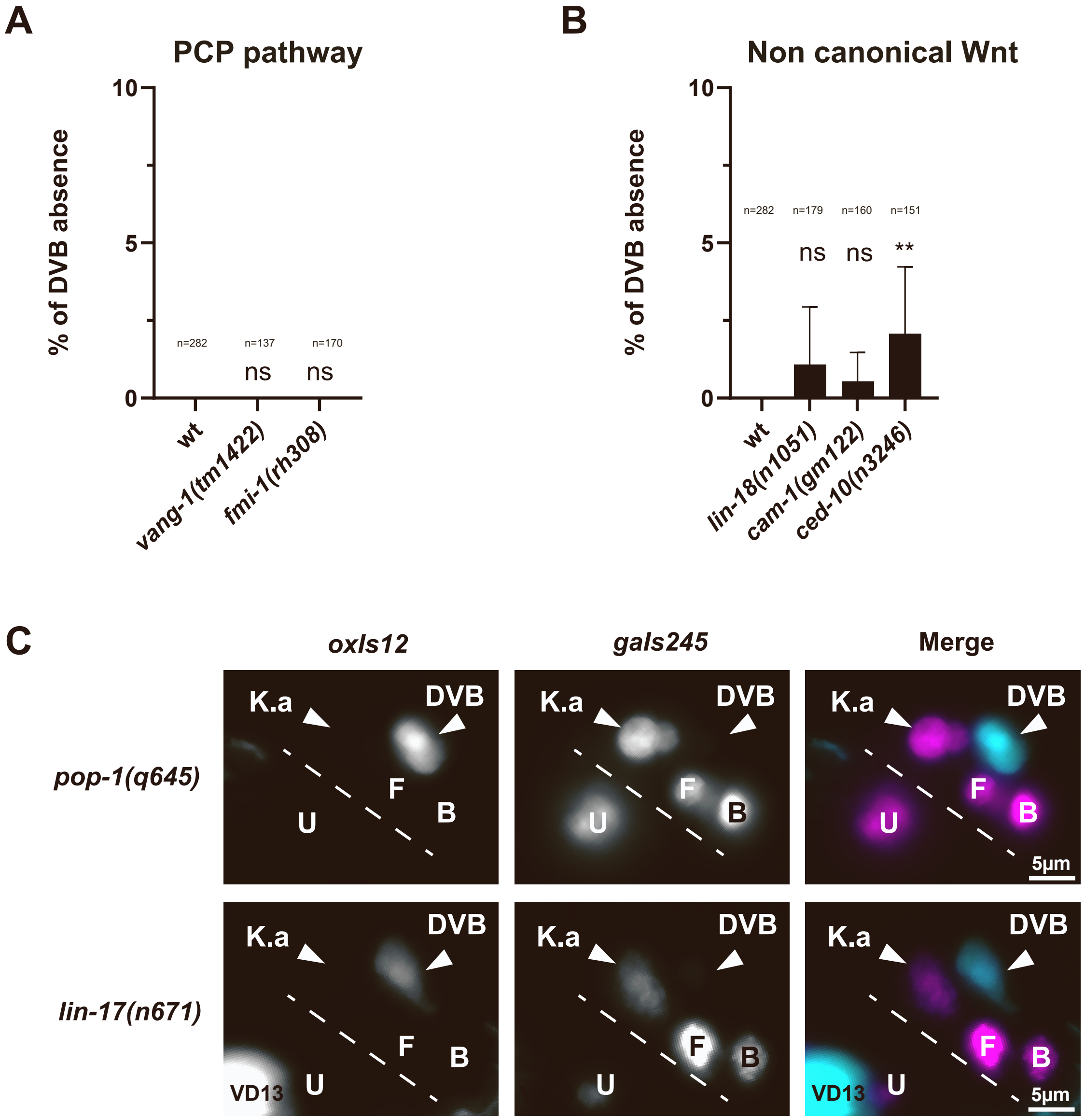

Figure S3. The non-canonical Wnt pathways are not required for K-to-DVB. Related to Figure 3.

(A) Histograms showing the percentage of "No DVB" worms in mutant backgrounds for genes of the PCP pathway.

(B) Histograms showing the percentage of "No DVB" worms in mutant backgrounds for the non-canonical Wnt-dependent pathways (*lin-18* and *cam-1*) or their downstream effectors (*ced-10*). n, total animal scored;.ns, non-statistically significant; **, p<0.005.

(C) When present, in a small percentage of *pop-1/TCF* (top) or *lin-17/FZD* (bottom) L4 mutants, the DVB neuron is formed from K posterior daughter. The positions of DVB, as observed with o*xIs12,* and of the K.a, U, F and B rectal cells, as observed using *gaIs245*, are indicated. VD13, GABAergic neuron. Dash line, rectal slit. Anterior is to the left and ventral to the bottom.

Fig. S4.

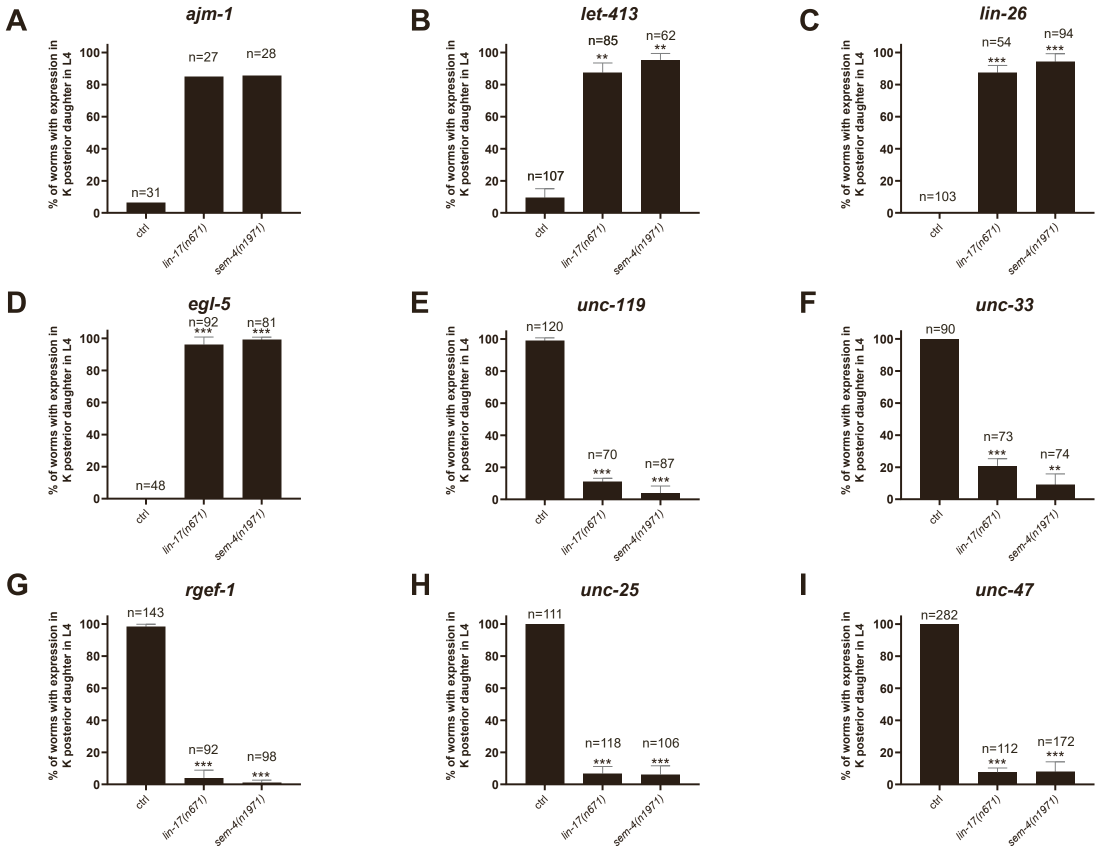

Figure S4. K.p cell remains rectal-epithelial in *lin-17/FZD* and *sem-4/SALL* mutants. Related to Figure 3 and Figure 4.

Quantification of the % of animals expressing (**A-C**) epithelial (*ajm-1, let-413* and *lin-26*), (**D**) rectal (*egl-5*), (**E-G**) pan-neuronal (*unc-119*, *unc-33* and *rgef-1*) and (**H, I**) GABAergic (*unc-25, unc-47*) reporters in K posterior daughter in *lin-17/F*ZD and *sem-4/SALL* mutant backgrounds, or DVB in wild type, in L4 larvae. n, total animal scored.

Fig. S5.

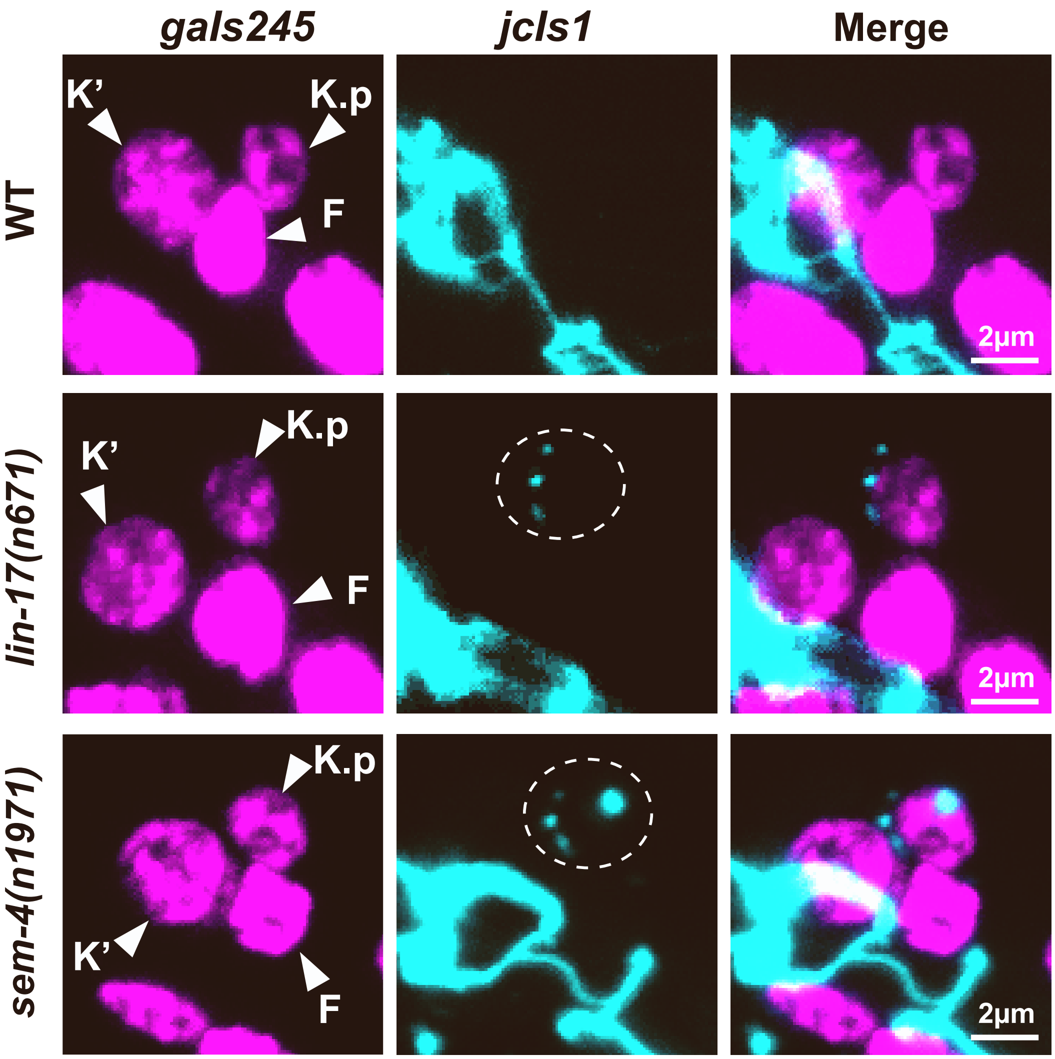

Figure S5. K.p cell expresses the apical junction protein AJM-1 in *lin-17/FZD* and *sem-4/SALL* mutants. Related to Figure 3 and Figure 4.

Confocal images of wild-type, *lin-17/FZD* and *sem-4/SALL* mutant backgrounds in L3 larvae carrying *gaIs245[col-34p::his-24::mcherry]* to visualize the rectal cell nuclei and *jcIs1[ajm-1::GFP]*. Patches of AJM-1 proteins are present in the K.p cell (dashed oval) in the mutant backgrounds, consistently with the mutant K.p retaining its epithelial identity.

Fig. S6.

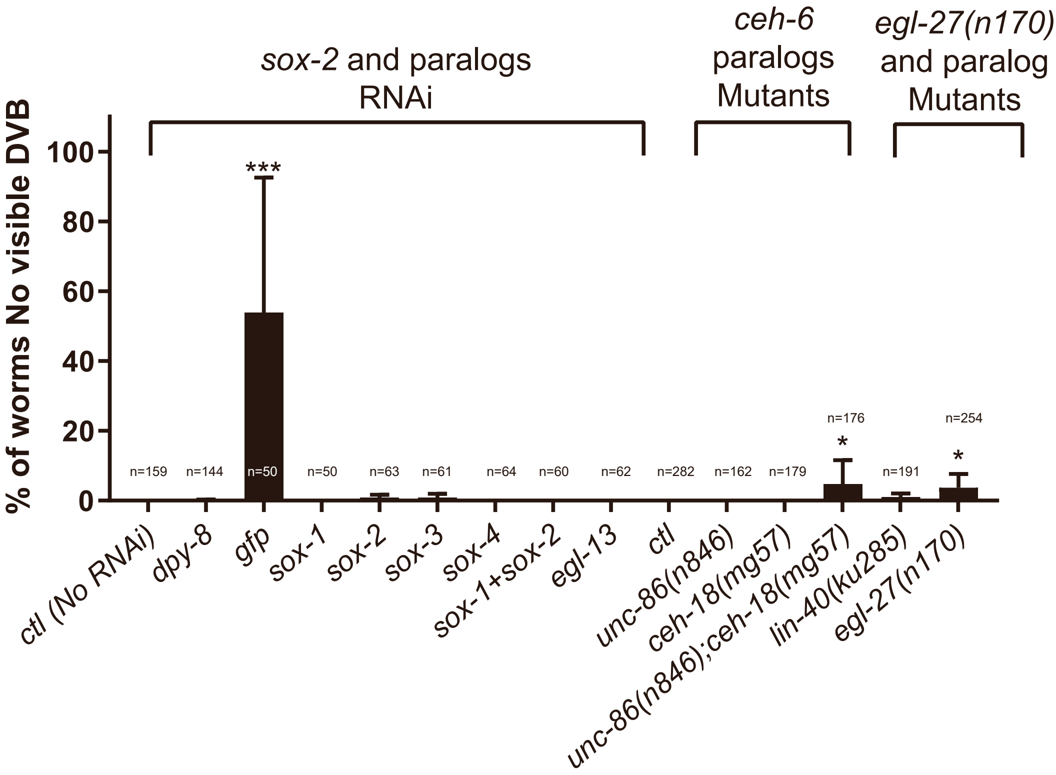

Figure S6. *sox-2*, *ceh-6* and *egl-27* paralogs do not seem to be required to form DVB. Related to Figure 4. Quantification of DVB defective L4 animals (as observed by *unc-47* expression) using RNAi in a sensitized *rrf*-3 mutant background to target *sox-2* paralogs (*dpy-8* and *gfp* RNAi represent controls). Mutants were used for paralogs of *ceh-6* (*unc-86(n846)* and *ceh-18(mg57)*) and *egl-27* (*lin-40(ku285)*). No obvious defects were observed, although RNAi was found to work poorly in the rectal cells. n, total animal scored.

Fig. S7.

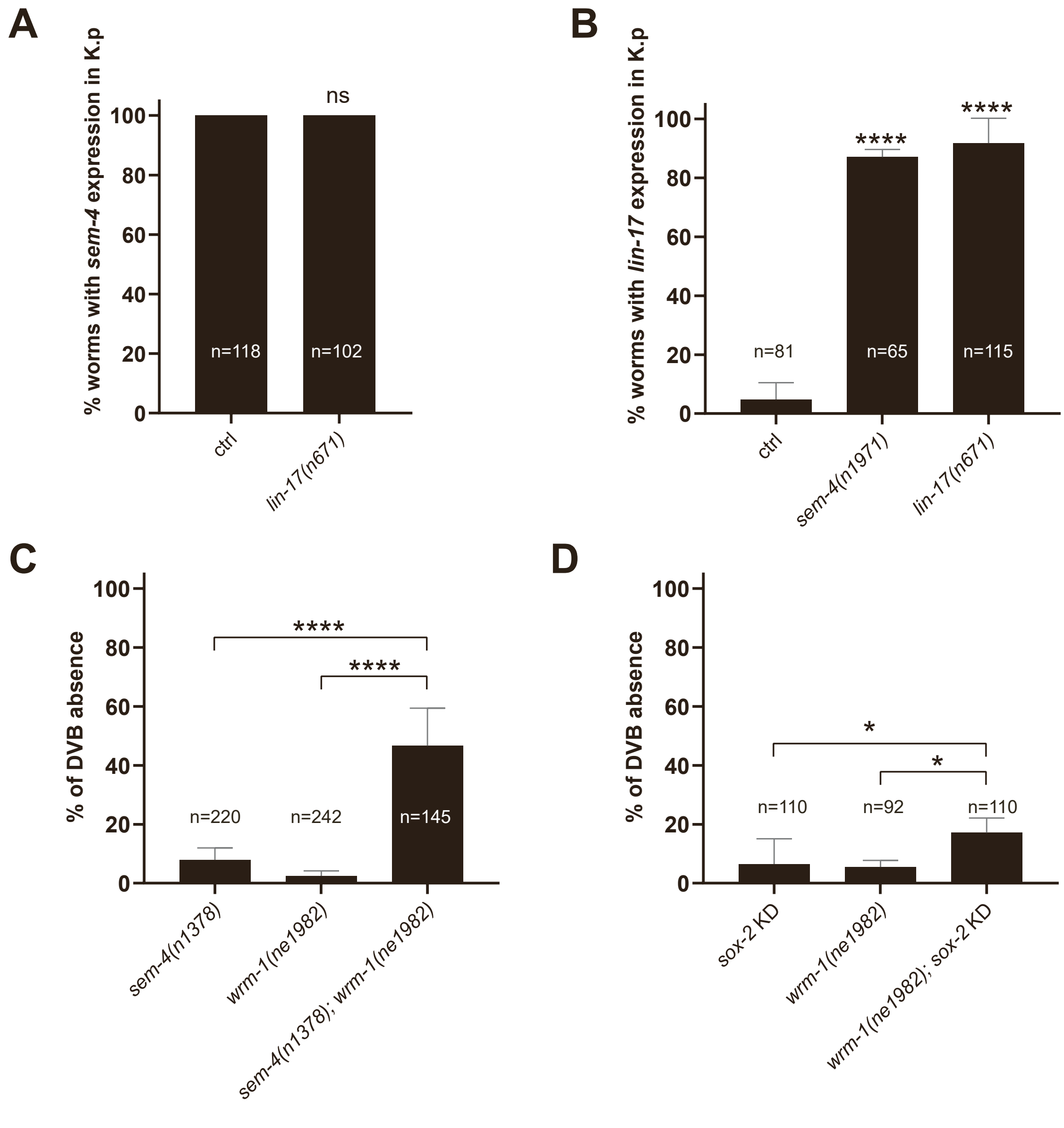

Figure S7. *sem-4/SALL* and the Wnt signaling pathway act in parallel to drive K-to-DVB Td. Related to Figure 5.

(A) Quantification of *sem-4/SALL* expression in K.p in wild type L4s and in *lin-17/Frizzled* mutant L4s.

(B) Quantification of *lin-17/FZD* expression in K.p in wild type L4s *vs sem-4/SALL* and *lin-17/FZD* mutants.

(C-D) Quantification of DVB defective L4 animals (as observed by *unc-47* expression using *krIs6* in A and *oxIs12* in B) in simple *sem-4(n1378)* (C), *sox-2* knock-down (using a nanobody strategy, D) and *wrm-1(n1982)* (C, D) mutants, or in *sem-4(n1378);wrm-1(n1982)* (C) and *wrm-1(n1982);sox-2* KD (D) double mutants, all raised at 25°C. n, total animal scored.

Fig. S8.

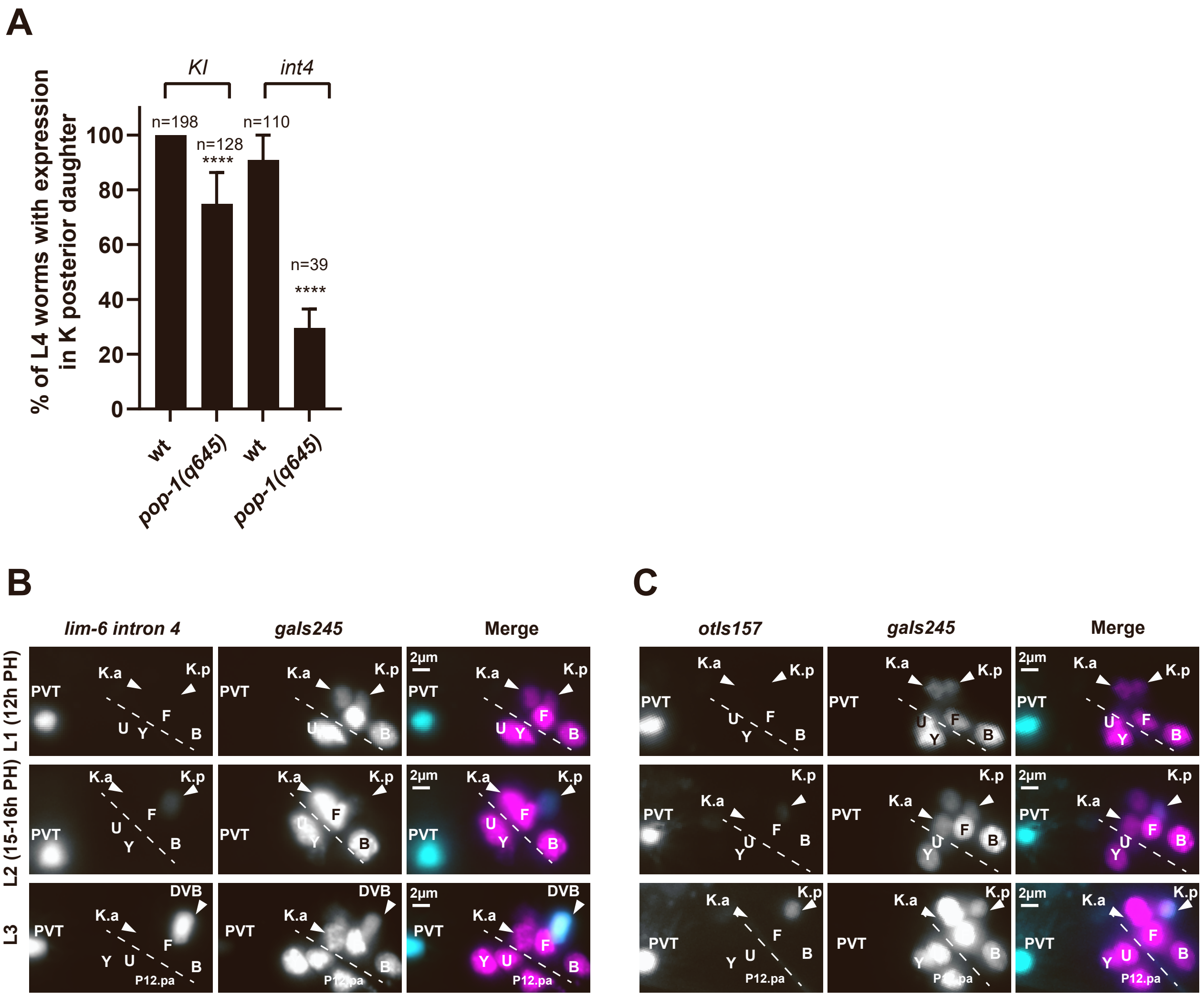

Figure S8. ***lim-6*** **is expressed early in K.p and its expression is affected in** ***pop-1/TCF*** **mutant. Related to Figure 5.**

(A) *lim-6* expression is impaired in *pop-1* mutant. Quantification of the % of L4 larvae expressing *lim-6::gfp* CRISPR (KI) and *lim-6 intron 4* transcriptional reporter (*int4*) in wild-type (DVB) and *pop-1(q645)* mutant (persistent K.p) backgrounds. Note that for the *pop-1(q645)* mutant, only viable homozygote (not balanced) mutant worms were analyzed. n, total animal scored.

(B-C) Time course expression of *lim-6(int4)::gfp (fpEx1111)* (B) and *lim-6r::gfp (otIs157)* (C) reporters in K.p/DVB (Cyan) in L1, 1h after the division (top), in an early L2 animal (middle) and in an L3 larva (bottom) where rectal cells are visualized with *gaIs245 (col-34p::his-24::mcherry;* magenta*)*.

For all pictures, dashed line, rectal slit. Anterior is to the left and ventral to the bottom.

Fig. S9.

A

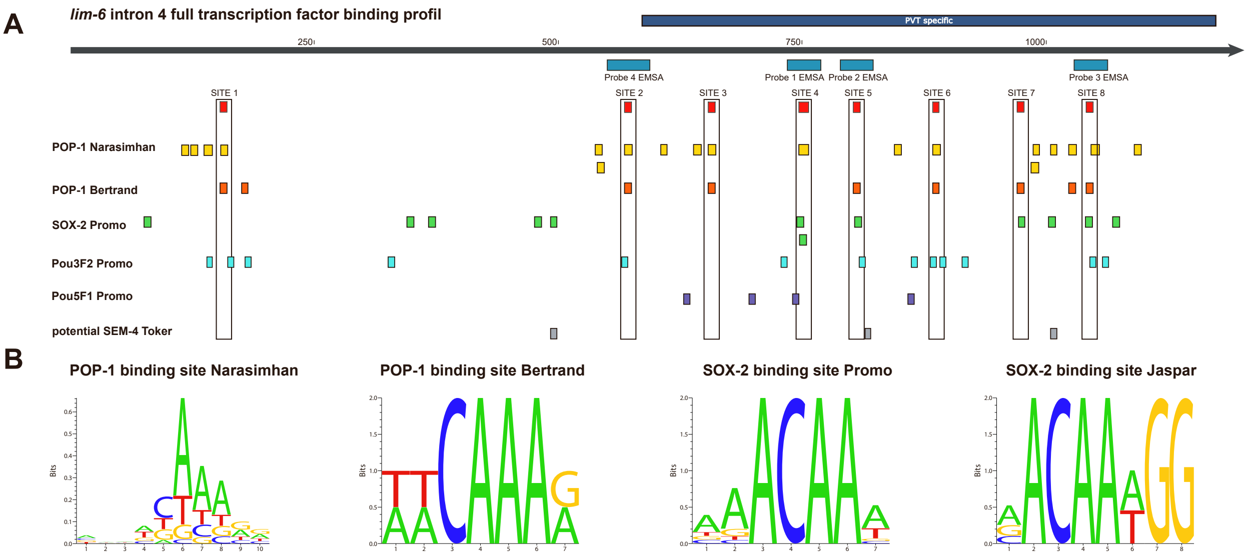

C

Site	Sequence	1st nucleotide Position in intron 4 (bp)	Identification method	Mutation in pSJ759
**Site 1**	TTCAAAG	154	Fimo: Narasimhan, 2015<br>Bertrand, 2009<br>MatInspector	
**Site 2**	ATCAAAA	569	Fimo: Narasimhan, 2015<br>Bertrand, 2009<br>MatInspector	ATCGTTA
**Site 3**	TTCAAAG	655	Fimo: Narasimhan, 2015<br>Bertrand, 2009<br>MatInspector	TTCGTTG
**Site 4**	ACAACAAACA	748	Fimo: Narasimhan, 2015	ACACCATGGA
**Site 5**	CTTTGTT	804 (rev)	Fimo: Narasimhan, 2015<br>Bertrand, 2009<br>MatInspector	CGCTAGC
**Site 6**	ATCAAAG	885	Fimo: Narasimhan, 2015<br>Bertrand, 2009<br>MatInspector	ATCCTCG
**Site 7**	CTTTGTT	972 (rev)	Bertrand, 2009<br>MatInspector	CCCGCGG
**Site 8**	AACAAAA	1043	Bertrand, 2009<br>MatInspector	CGGCCGA

D

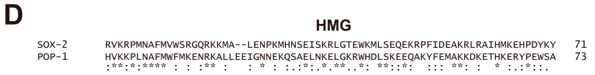

E

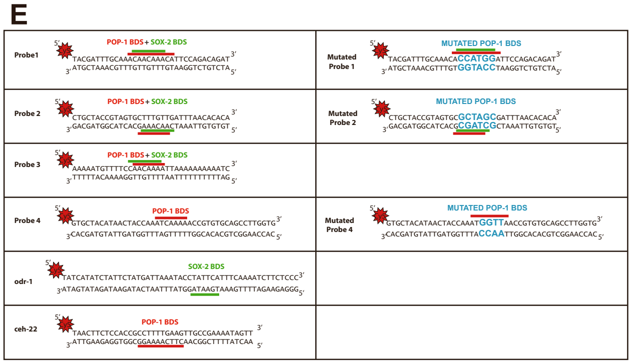

Figure S9. Analysis of SOX-2 and POP-1/TCF binding sites in *lim-6* intron 4. Related to Figure 5.

(A) The intron 4 of *lim-6* was analyzed using different tools (See Methods). Here the POP-1 binding site prediction using the Matrix of Narasimhan and the consensus of Bertrand, 2009, are represented. The SOX-2, POU3F2 and POU5F1 binding sites were predicted by Promo and the SEM-4 consensus site published in Toker, 2003 was used.

(B) Sequence logo for POP-1 and SOX-2 binding sites showing the sequence similarities.

(C) Table summarizing the binding sites on which we have focused our efforts in this study. Binding sites predicted by more than one approach, or because of the presence of two consecutive binding sites for SOX-2 (site 4), were selected. The mutations introduced into the *lim-6* transcriptional reporter to abolish POP-1 binding are presented on the right. Note that these mutations most probably abolish also SOX-2 binding due to the very close similarity of their predicted binding sites.

(D) This binding site similarity can be explained by the sequence similarity of the HMG domains present in SOX-2 and POP-1.

(E) Sequence of the probes used in this study for the gel shift experiments. Note that probe 3 displayed poor annealing due to its AT rich sequence and therefore was not further used.

Fig. S10.

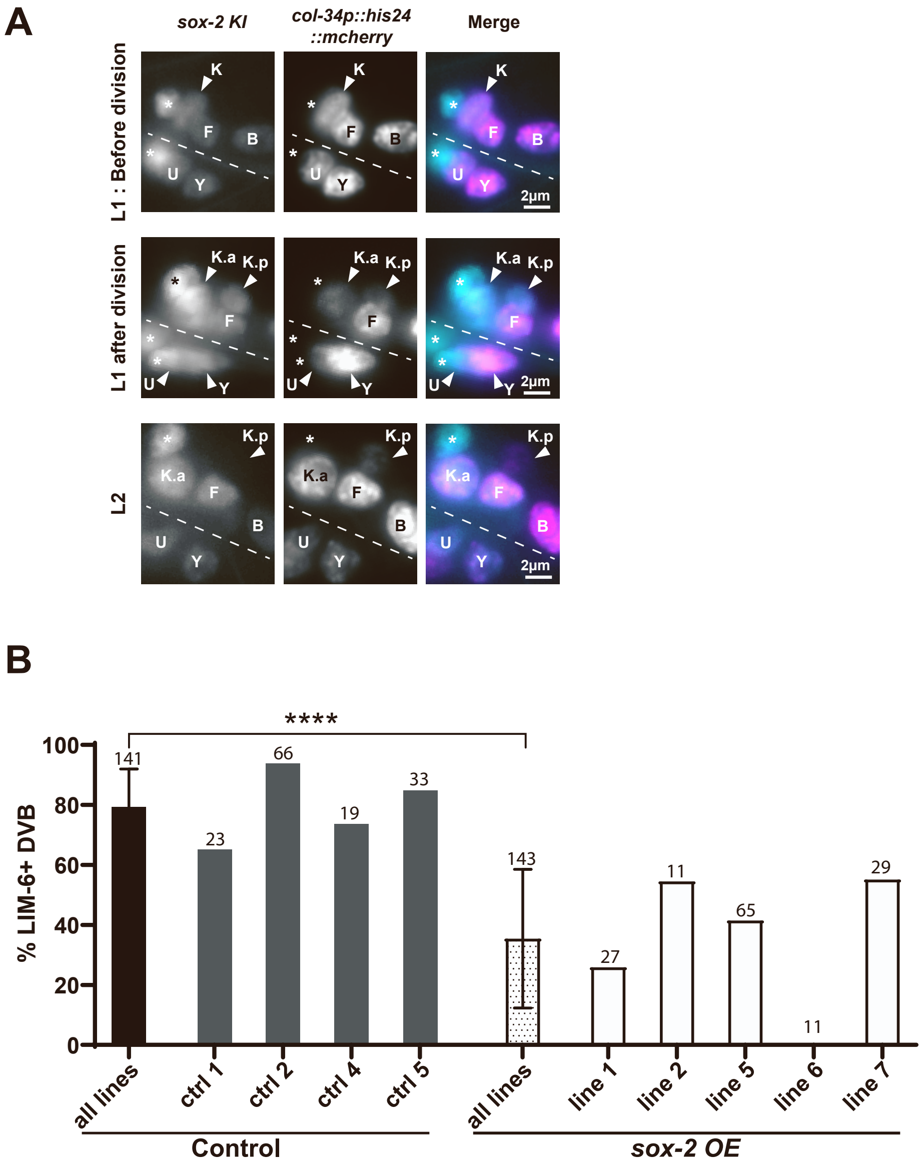

Figure S10. *sox-2* is expressed in K.p after K division and it is subsequently downregulated during DVB differentiation. Overexpression of *sox-2* prevents *lim-6* expression. Related to Figure 5A.

(A) Fluorescent images of *gfp::sox-2* KI and *col-34p::his-24::mcherry* in the rectum of a wild-type L1 animal before K division (top), in an L1 animal after K division (14 cells in the gonad; middle) and in an L2 animal (bottom). Note that K.a continues to express *sox-2* over time whereas expression fades away in K.p during its conversion. White stars indicate the rectal gland cells; the rectal cell position is indicated on the pictures; dashed line, rectal slit; anterior is left and ventral is bottom.

(B) The rectal *col-34* promoter was used to overexpress (OE) SOX-2 in K.p along with a co-injected *lim-6(int4)::mCherry* reporter. The % of L4 animals displaying *lim-6* expression in DVB are represented. Black bar, all results obtained for the control lines (*lim-6(int4)::mCherry* alone) and dark grey bars, each individual line data respectively; Dotted bar, all results obtained for the SOX-2 overexpressing lines, followed by each individual SOX-2(OE) line data (white bars). Note that transgenic lines overexpressing SOX-2 are difficult to retrieve and maintain, and throw few transgenic animals: transgenics in the F2 generation were usually the only animals that could be scored. The total number of animals scored is indicated above each bar. ****, p<0.0001.

Fig. S11.

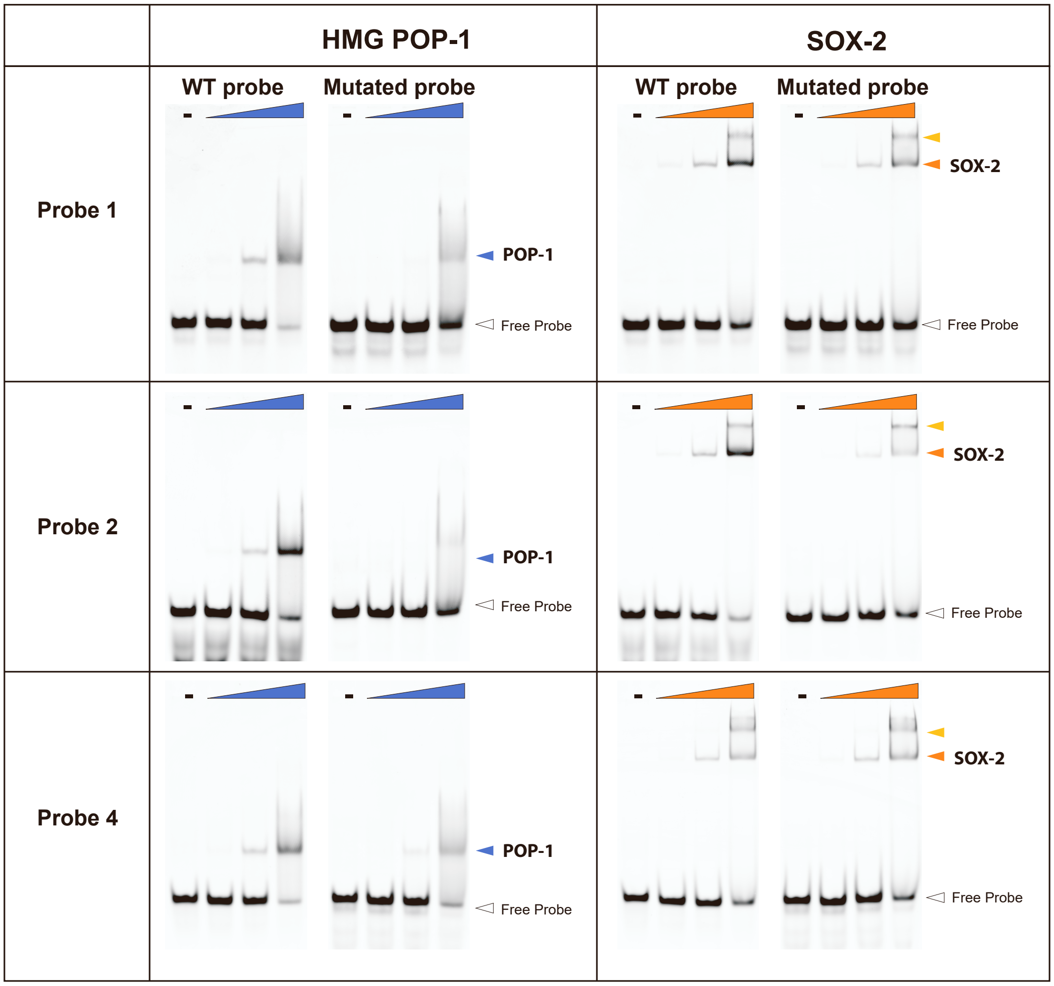

Figure S11. Gel shift experiments show independent binding capacities of POP-1 and SOX-2 to probes 1, 2 and 4. Related to Figure 5E, F.

Increasing concentration (5nm, 50nM and 500nM) of purified HMG-POP-1 and SOX-2 were incubated with wild type or mutated probes 1, 2 and 4 bound to the Cy5 fluorophore. Note that the probe 4 which does not bear canonical SOX-2 binding site is able to bind SOX-2. As, in addition, the mutation of the POP-1 binding site does not seem to affect this binding, it is likely that a non-predicted SOX-2 binding site is present. Probe 3 was also able to bind both SOX-2 and HMG-POP-1, although results for are not presented because this probe annealed poorly, most probably due to its AT-rich sequence. Blue arrowhead, POP-1 bound to the probe; orange arrowhead, SOX-2 bound to the probe; light orange arrowhead, a second SOX-2 shifted band appears at high SOX-2 concentrations; open arrowhead, unbound probe.

Fig. S12.

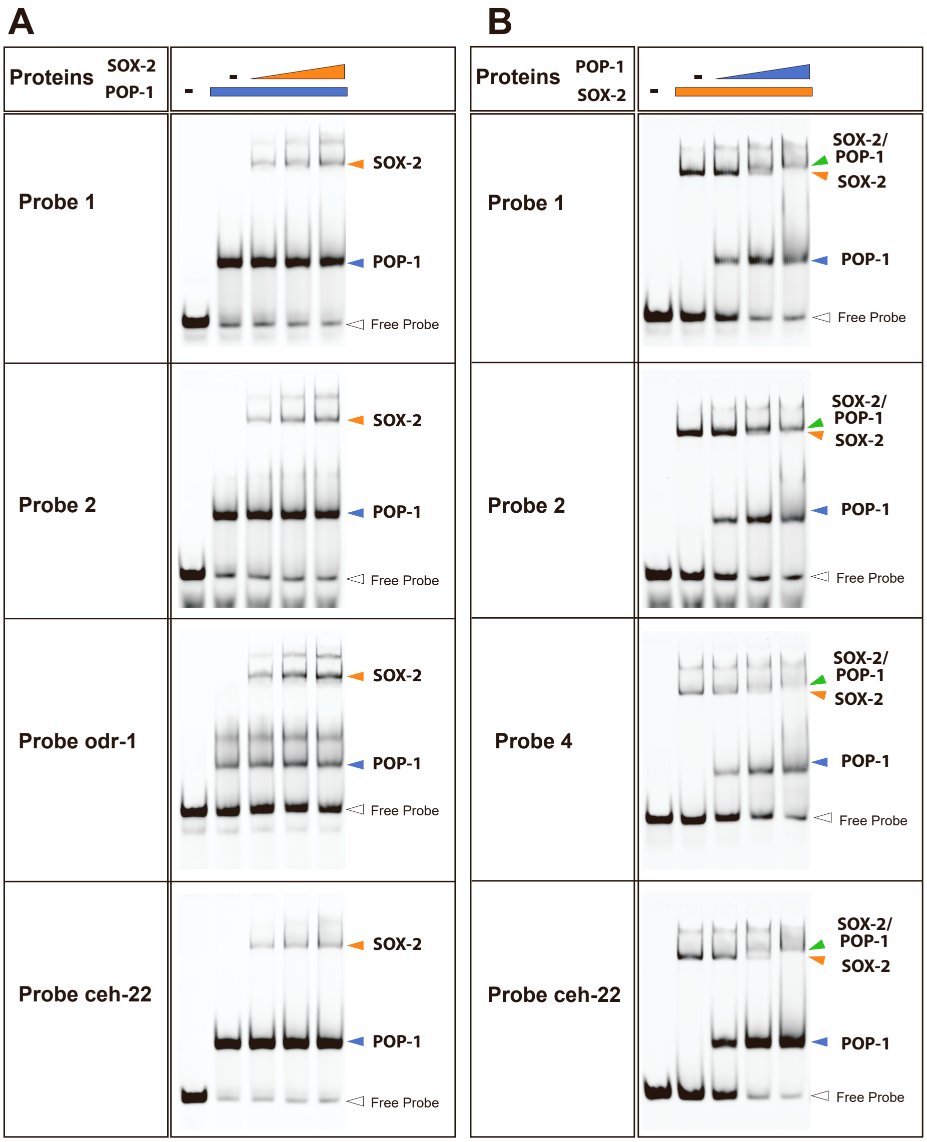

Figure S12. Gel shift experiments show binding capacities of POP-1 and SOX-2 when co-incubated. Related to Figure 5E, F.

(**A**) Increasing quantity of SOX-2 (125nM-250nM-500nM) was added to a mix of HMG-POP-1 and Cy-5-dsProbe #1, #2, *odr-1* (known SOX-2 target, Alqadah, 2015) and *ceh-22* (known POP-1 target, Lam, 2007, Bhambhani, 2014). (**B**) Increasing quantity of HMG-POP-1 (125nM-250nM-500nM) was added to a mix of SOX-2 and Cy-5-dsProbe #1,# 2, #4 and *ceh-22*. Increasing quantity of HMG-POP-1 shows an increasing binding to all the probes as well as an upper shift, most probably corresponding to a HMG-POP-1-SOX-2-Probe complex.

Blue arrowhead, POP-1 bound to the probe; orange arrowhead, SOX-2 bound; green arrowhead, POP-1 and SOX-2 bound; open arrowhead, unbound probe.

Fig. S13.

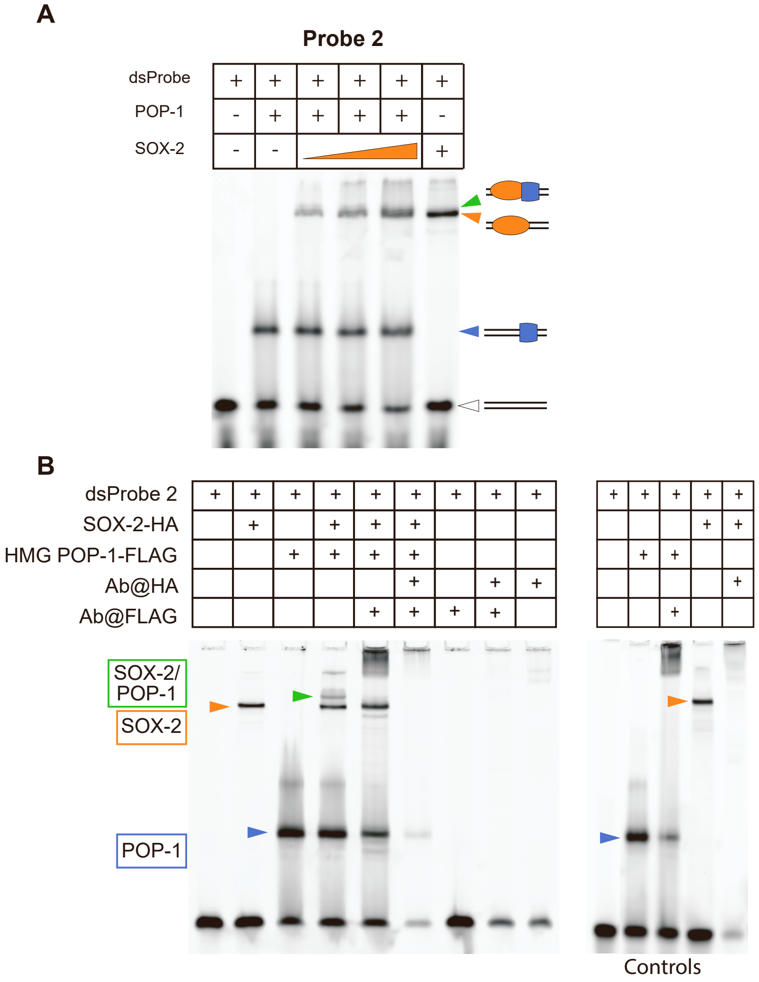

Figure S13. Antibody supershift EMSA analysis of SOX-2 and POP-1 co-binding. Related to Figure 5E, F.

(**A**) Representative EMSA assay on Probe 2 revealing single binding of SOX-2 (orange arrow head) and HMG-POP-1 (blue arrow head) as well as co-binding (upper band, green arrow head).

(**B**) This upper band was totally upshifted after pre-incubation of the complex with an anti-FLAG antibody (Ab@FLAG) against HMG-POP-1-FLAG.

Various combinations of antibodies/protein/probe complexes were used for controls as indicated.

Table S1. Summary of all cell markers expression.

C. elegans genes	Human ortholog	Reporter	WT				*sem-4*	*lin-17*	References reporter	Ref observation
			L1	early L2		L4	L4	L4		
			K	K.a	K.p	DVB	K post. daughter	K post. daughter		
**Epithelial markers**										
dlg-1	*DLG*	*mcIs46[dlg-1::rfp]*	+	+	-	-	N.D	N.D	Diogon et al. (2007)	This study
ajm-1	*AJM1*	*jcIs1[ajm-1::gfp]*	+	+	-	-	+	+	Mohler et al. (1998)	This study; Mohler et al. (1998)
hmr-1	Cadherin	*fpIs17[hmr-1::gfp]*	+	+	-	-	N.D	N.D	This study	This study
let-413	*SCRIB*	*fpEx1062[let-413a::gfp::pest]*	+	+	+	-	+	+	This study	This study
lin-26	Zinc-finger transcription factor	*fpIs110[lin-26rectalp::gfp]*	+	+	+	-	+	+	Labouesse et al. (1996); this study	Labouesse et al. (1996); this study
**Rectal markers (also in other cells, to visualise the rectal cells)**										
sem-4	*SALL* transcription factor	*syb1287[sem-4::gfp]*	+	+	+	+	ND	+	This study	This study
sox-2	*SOX* transcription factor	*syb737[gfp::sox-2]*	+	+	+*	-	+	+	This study	This study
ceh-6	*POU* transcription factor	*syb972[gfp::ceh-6]*	+	+	+*	-	+	+	This study	This study
egl-5	*HOX* transcription factor	*bxIs7[egl-5::gfp]*	+	+	+	-	ND	ND	Teng et al. (2004)	This study
col-34	Cuticle collagen gene	*gaIs245[col-34p::his-24::mcherry]*	+	+	+	-	+	+	Zuryn et al. (2014)	This study
got-1.2	*GOT1*	*sIs11174[rCesT01C8.5::gfp+p Ceh361]*	+	+	+	-	ND	ND	McKay et al. (2003)	This study
**Pan-neuronal markers**										
unc-33	*DPYS*	*otIs117[unc-4(+) ; unc-33p::GFP]*<br>*otIs118[unc-33::GFP; unc-4(+)]*	-	-	-	+	-	-	McKay et al. (2003)	This study
unc-119	*UNC119*	*edIs6[unc-119::gfp; rol-6]*	-	-	-	+	-	-	Maduro and Pilgrim (1995); Praitis et al. (2001)	This study
rgef-1	RASGRP3	*otIs173 [F25B3.3::DsRed2; ttx-3promB::GFP]*	-	-	-	+	-	-	Benard et al. (2009)	This study
**DVB Terminal selector**										
lim-6	*LMX1B*	*syb971[lim-6::gfp]*<br>*otIs157[lim-6r::GFP]*	-	-	+	+	-	-	This study	Hobert et al. (1999); this study
**GABAergic markers**										
unc-47	*SLC32A1*	*oxIs12[unc-47p::gfp]*<br>*krIs6[unc-47p::DsRed2]*	-	-	-	+	-	-	McIntire et al. (1997)<br>Teuliere et al. (2011)	McIntire et al. (1997); this study<br>Teuliere et al. (2011)
unc-25	*GAD*	*juIs8[unc-25p::gfp]*	-	-	-	+	-	-	Jin et al. (1999)	Jin et al. (1999) ; this study

*, expression is seen in K.p after its birth, and disappears as *lim-6* expression appears (see Fig. 5A).

Table S2. Strain list.

C. elegans strain	Identifier
rrf-3(pk1426) II ; oxIs12[unc-47::gfp; lin-15(+)] X	IS17
lin-5(ev571) II; gaIs245[col-34p::his-24::mcherry; unc-119(+)] V ; oxIs12[unc-47p::gfp; lin-15(+)] X	IS1118
sem-4(n1971) bxIs7[egl-5p(6,5kb)::gfp; lin-15(+)] I; otIs173[rgef-1p::dsred2; ttx-3p::gfp] III	IS1208
sem-4(n197) I; otIs117[unc-4(+); unc-33p::gfp] IV; gaIs245[col-34p::his-24::mcherry; unc-119(+)] V	IS1210
gaIs245[col-34p::his-24::mcherry; unc-119(+)] V; oxIs12[unc-47p::gfp; lin-15(+)] X	IS1299
egl-5(n945) III; gaIs245[col-34p::his-24::mcherry; unc-119(+)] V; oxIs12[unc-47p::gfp; lin-15(+)] X	IS1332
lin-17(n671) I; gaIs245[col-34p::his-24::mcherry; unc-119(+)] V; oxIs12[unc-47p::gfp; lin-15(+)] X	IS1370
fpIs17[hmr-1::gfp]; gaIs245[col-34p::his-24::mcherry; unc-119(+)] V	IS1374
wrm-1(ne1982) III; gaIs245[col-34p::his-24::mcherry; unc-119(+)] V; oxIs12[unc-47p::gfp; lin-15(+)] X	IS1432
sem-4(n1971) I; gaIs245[col-34p::his-24::mcherry; unc-119(+)] V; oxIs12[unc-47p::gfp; lin-15(+)] X	IS2968
unc-86(n846) III; gaIs245[col-34p::his-24::mcherry; unc-119(+)] V; oxIs12[unc-47p::gfp; lin-15(+)] X	IS3097
fpIs110[lin-26p::gfp; rol-6(su1006)] IV; gaIs245[col-34p::his-24::mcherry; unc-119(+)] V	IS3107
egl-27(ok1670) II; gaIs245[col-34p::his-24::mcherry; unc-119(+)] V ; oxIs12[unc-47p::gfp; lin-15(+)] X	IS3113
gaIs245[col-34p::his-24::mcherry; unc-119(+)] V; fpEx1062[let-413a::gfp::pest; myo-2p::gfp]	IS3119
gaIs245[col-34p::his-24::mcherry; unc-119(+)] V; oxIs12[unc-47p::gfp; lin-15(+)] X; fpEx955[Δ(-2846pb to -102)ceh-6p::gfp::ceh-6; odr-1::rfp]	IS3120
ceh-6(gk665) I; gaIs245[col-34p::his-24::mcherry; unc-119(+)] V; oxIs12[unc-47p::gfp; lin-15(+)] X; fpEx955[Δ(-2846pb to -102)ceh-6p::gfp::ceh-6; odr-1::rfp]	IS3122
gaIs245[col-34p::his-24::mcherry; unc-119(+)] V; oxIs12[unc-47p::gfp; lin-15(+)] X; fpEx788[egl-5p(1,3kb)::sox-2(antisens); rol-6(su1006)]	IS3142
lin-40(ku285) V; oxIs12[unc-47p::gfp; lin-15(+)] X	IS3146
sem-4(n1971) I; gaIs245[col-34p::his-24::mcherry; unc-119(+)] V; fpEx1062[let-413a::gfp::pest; myo-2p::gfp]	IS3176
gaIs245[col-34p::his-24::mcherry; unc-119(+)] V; juIs8[unc-25p::gfp; lin-15(+)]	IS3298
edIs6[unc-119p::gfp; rol-6(su1006)] IV; gaIs245[col-34p::his-24::mcherry; unc-119(+)] V	IS3327
lin-17(n671)I; edIs6[unc-119p::gfp; rol-6(su1006)] IV; gaIs245[col-34p::his-24::mcherry; unc-119(+)] V	IS3328

gaIs245[col-34p::his-24::mcherry; unc-119(+)] V; otIs118[unc-33p::gfp; unc-4(+)]	**IS3329**
lin-17(n671)I; gaIs245[col-34p::his-24::mcherry; unc-119(+)] V; otIs118[unc-33p::gfp; unc-4(+)]	**IS3330**
lin-17(n671)I; gaIs245[col-34p::his-24::mcherry; unc-119(+)] V; juIs8 [unc-25p::gfp; lin-15(+)]	**IS3335**
jcIs1[ajm-1::gfp; rol-6(su1006)] IV; gaIs245[col-34p::his-24::mcherry; unc-119(+)] V	**IS3339**
lin-17(n671) I; fpIs110[lin-26p::gfp; rol-6(su1006)] IV; gaIs245[col-34p::his-24::mcherry; unc-119(+)] V	**IS3349**
lin-17(n671) I; jcIs1[ajm-1::gfp; rol-6(su1006)] IV; gaIs245[col-34p::his-24::mcherry; unc-119(+)] V	**IS3357**
gaIs245[col-34p::his-24::mcherry; unc-119(+)] V; fpEx1111[lim-6int4::gfp; coel::dsred]	**IS3379**
lin-17(n671) I; gaIs245[col-34p::his-24::mcherry; unc-119(+)] V; fpEx1062[let-413a::gfp::pest; myo-2p::gfp]	**IS3383**
lin-17(n671) I; gaIs245[col-34p::his-24::mcherry; unc-119(+)] V; fpEx1111[lim-6int4::gfp; coel::dsred]	**IS3420**
gaIs245[col-34p::his-24::mcherry; unc-119(+)] V; sox-2(syb737[gfp::linker::sox-2]) X	**IS3423**
wyIs75[unc-47p::dsred; exp-1p::gfp; odr-1p::rfp] III; vang-1(tm1422)X	**IS3433**
unc-73(e936) dpy-5(e61) I ; gaIs245[col-34p::his-24::mcherry; unc-119(+)] V ; oxIs12[unc-47p::gfp; lin-15(+)] X	**IS3452**
lin-17(n671) I; gaIs245[col-34p::his-24::mcherry; unc-119(+)] V; sox-2(syb737[gfp::linker::sox-2]) X	**IS3457**
sem-4(n1971) I; gaIs245[col-34p::his-24::mcherry; unc-119(+)] V; sox-2(syb737[gfp::linker::sox-2]) X	**IS3458**
dsh-1(ok1445) II; gaIs245[col-34p::his-24::mcherry; unc-119(+)] V; oxIs12[unc-47p::gfp; lin-15(+)] X	**IS3464**
egl-27(ok1670) II; wyIs75[unc-47p::dsred; exp-1p::gfp; odr-1p::rfp] III; him-5(e1490)V	**IS3469**
egl-5(n945) III; syIs50[cdh-3p::gfp; dpy-20(+)]	**IS3475**
egl-20(n585) IV ; gaIs245[col-34p::his-24::mcherry; unc-119(+)] V; oxIs12[unc-47p::gfp; lin-15(+)] X	**IS3485**
lin-44(n1792)I; gaIs245[col-34p::his-24::mcherry; unc-119(+)] V; oxIs12[unc-47p::gfp; lin-15(+)] X	**IS3486**
lin-44(n1792) I ; egl-20(n585) IV ; gaIs245[col-34p::his-24::mCherry; unc-119(+)] V ; oxIs12[unc-47p::gfp; lin-15(+)] X	**IS3487**
gaIs245[col-34p::his-24::mcherry; unc-119(+)] V; lim-6(nr2073) oxIs12[unc-47p::gfp; lin-15(+)] X	**IS3490**
par-1(zu310) gaIs245[col-34p::his-24::mcherry; unc-119(+)] V; oxIs12[unc-47p::gfp; lin-15(+)] X	**IS3491**
fmi-1(rh308) gaIs245[col-34p::his-24::mcherry; unc-119(+)] V; oxIs12[unc-47p::gfp; lin-15(+)] X	**IS3511**
dsh-1(ok1445) mig-5(tm2639) II; oxIs12[unc-47p::gfp; lin-15(+)] X	**IS3512**
wyIs75[unc-47p::dsred; exp-1p::gfp; odr-1p::rfp] III; gaIs245[col-34p::his-24::mcherry; unc-119(+)] V; sox-2(syb737[gfp::linker::sox-2]) X; fpEx1156[egl-5p(6.5kb)::nanobodyGFP::zif-1; coel::gfp; pBSK]	**IS3521**

gpr-1(ok2126) III; gaIs245[col-34p::his-24::mcherry; unc-119(+)] V; oxIs12[unc-47p::gfp; lin-15(+)] X	**IS3530**
sem-4(n1971)I; edIs6[unc-119p::gfp; rol-6(su1006)] IV; gaIs245[col-34p::his-24::mcherry; unc-119(+)] V	**IS3537**
sem-4(n1971)I; gaIs245[col-34p::his-24::mcherry; unc-119(+)] V; otIs118[unc-33p::gfp; unc-4(+)]	**IS3539**
gaIs245[col-34p::his-24::mcherry; unc-119(+)] V; ceh-6(syb972[gfp::linker::ceh-6]) X	**IS3540**
lin-17(n671) bxIs7[egl-5(6.5kb)::gfp; lin-15(+)] I; otIs173[rgef-1p::dsred2; ttx-3pB::gfp]III	**IS3583**
hT2[bli-4(e937) let-?(q782) qIs48] (I;III)/pop-1(q645)I ; gaIs245[col-34p::his-24::mcherry; unc-119(+)] V; oxIs12[unc-47p::gfp; lin-15(+)] X	**IS3596**
hT2[bli-4(e937) let-?(q782) qIs48] (I;III)/pop-1(q645)I; gaIs245[col-34p::his-24::mcherry; unc-119(+)] V; fpEx1111[lim-6int4::gfp; coel::dsred]	**IS3600**
otIs173[rgef-1p::dsred2; ttx-3pB::gfp]III; oxIs12[unc-47p::gfp; lin-15(+)] X	**IS3604**
lin-5(ev571)II; gaIs245[col-34p::his-24::mcherry; unc-119(+)] V; fpIs101[col-34p::ph::gfp; odr-1p::dsRed] X	**IS3619**
sem-4(n1971)I; gaIs245[col-34p::his-24::mcherry; unc-119(+)] V; lim-6(syb971[lim-6::linker::gfp])X	**IS3632**
lin-17(n671)I; gaIs245[col-34p::his-24::mcherry; unc-119(+)] V; lim-6(syb971[lim-6::linker::gfp])X	**IS3669**
gaIs245[col-34p::his-24::mcherry; unc-119(+)] V; lim-6(syb971[lim-6::linker::gfp])X	**IS3677**
lin-17(n671) I; gaIs245[col-34p::his-24::mcherry; unc-119(+)] V; ceh-6(syb972[gfp::linker::ceh-6]) X	**IS3702**
sys-1(q544) I; gaIs245[col-34p::his-24::mcherry; unc-119(+)] V; oxIs12[unc-47p::gfp; lin-15(+)] X	**IS3718**
krIs6[unc-47::DsRed2; lin-15(+)] II ; gaIs245[col-34p::HIS-24::mCherry; unc-119(+)] V; fpEx1295(pcr fragment col-34p::gfp::cki-1(gDNA), myo-2p::mCherry]	**IS3950**
krIs6[unc-47::DsRed2; lin-15(+)] II ; gaIs245[col-34p::HIS-24::mCherry; unc-119(+)] V; fpEx1296(pcr fragment col-34p::gfp::cki-1(gDNA), myo-2p::mCherry]	**IS3951**
krIs6[unc-47::DsRed2; lin-15(+)] II ; gaIs245[col-34p::HIS-24::mCherry; unc-119(+)] V; fpEx1297(pcr fragment col-34p::gfp::cki-1(cDNA), myo-2p::mCherry]	**IS3952**
krIs6[unc-47::DsRed2; lin-15(+)] II ; gaIs245[col-34p::HIS-24::mCherry; unc-119(+)] V; fpEx1298(pcr fragment col-34p::gfp::cki-1(gDNA), myo-2p::mCherry]	**IS3972**
krIs6[unc-47::DsRed2; lin-15(+)] II ; gaIs245[col-34p::HIS-24::mCherry; unc-119(+)] V; fpEx1299(pcr fragment col-34p::gfp::cki-1(gDNA), myo-2p::mCherry]	**IS3973**
krIs6[unc-47::DsRed2; lin-15(+)] II ; gaIs245[col-34p::HIS-24::mCherry; unc-119(+)] V; fpEx1300(pcr fragment col-34p::gfp::cki-1(gDNA), myo-2p::mCherry]	**IS3974**

Table S3. Oligonucleotides list.

Oligo name	Sequence	Use
**BDT950**	CTGAATCCGGATCCATCATCATGTCGACTGCAGAATTCGAAGCTT GTCGACGGAGCTC	*sox-2* antisense construct
**BDT952**	CTTGGAGGGTACCTAGGAGCTCGATATCTAGAAGAGGTAACATG GGATTGGGA	*sox-2* antisense construct
**EB110F**	AGAAGACCGCCCCTCTTTTGA	Genotyping *ceh-6(syb972)*
**EB110R**	GGCTGCCTCCATCTCGTTCT	Genotyping *ceh-6(syb972)*
**EB5F**	TCCAGTCTCTTCAGGTCAGTGATCT	Genotyping *egl-27(ok1670)*
**EB5R**	CGAGATTTCCAAATTCTTACCCGACTG	Genotyping *egl-27(ok1670)*
**EB6R**	GTGTAATTGACAGCGATGATGATGAAGG	Genotyping *egl-27(ok1670)*
**LIN5 FW 01**	GACAAGACCAAGTTATCGGC	Genotyping *lin-5(ev571),* digest w/ BglII
**LIN5 RV 01**	CCCATTGACTGAAATTCTTCG	Genotyping *lin-5(ev571),* digest w/ BglII
**mcm124F**	GAACTACAACTACTTTGGTCAACCATTGGGCCCTGCCACGTTTCC CCCAT	Genotyping *egl-5(n945),* digest w/ NcoI
**mcm124R**	CGTAAGATAGCATATAGGGTCAGACG	Genotyping *egl-5(n945),* digest w/ NcoI
**mcm125F**	CCGCGCCATTGACACCGATTTGGTAC	Genotyping *sem-4(n1971),* digest w/ Acc65I
**mcm125R**	CCTAACAAGCTAGCCTTTTCAGTTACAAAAACATCTCTCTTAACTG GGTA	Genotyping *sem-4(n1971),* digest w/ Acc65I
**oCG347 rev début GFP**	CCACTGACAGAAAATTTGTGCCC	Genotyping *sem-4 (syb1287)* and sequencing
**oCG368 sens PEST**	CTTAGCCATGGCTTCCCGCCGGCGGTGGCGGCGCAGGATGATG GCACGCTGCCCATGTCTTGTGCCCAGGAGAGCGGGATGGACCGT CACCCTGCAGCCTGTGCTTCTGCTAGGATCAAT	PEST sequence fw
**oCG369 rev PEST**	ATTGATCCTAGCAGAAGCACAGGCTGCAGGGTGACGGTCCATCC CGCTCTCCTGGGCACAAGACATGGGCAGCGTGCCATCATCCTGC GCCGCCACCGCCGGCGGGAAGCCATGGCTAAG	PEST sequence rv
**oCG370 sens MW PEST**	GGATTACACATGGCATGGATGAACTATACAAACTTAGCCATGGCT TCCCGCCGGCGGTGGC	Cloning *pest* sequence into pML801
**oCG371 rev MW PEST**	GGTAGCGACCGGCGCTCAGTTGGAATTCTACGAATGCTACACATT GATCCTAGCAGAAGCACAGGCTG	Cloning *pest* sequence into pML801
**oCG381 lin-26p f**	CAACTTGGAAATGAAATAAGCTTGCATGGATCCGACGTCTTCCCA TTGTCTTCCATTCATCTT	Cloning of the lin-26 rectal specific promoter into pPD97.82
**oCG382 lin-26p r**	GCTGAAAAGTGTCTAGAGTCGACCAAGGCCTGCAGCTGAAAATAA TCAATTAAAAATTTTAAAAAAAGTAAGCGAGGG	Cloning of the lin-26 rectal specific promoter into pPD97.82
**oCG390 for NLS1 kpnI**	AGGGTACCGAGCTCAGAAAAAATGACAGC	Cloning *2nls* into pPD95.75
**oCG391 rev GFP XhoI**	GGGTATCTCGAGAAGCATTGAACACCATAACAGAAAG	Cloning *2nls* into pPD95.75
**oCG411 sens egr-1 ku285**	GCCCCAAAAGCCTGAAAAAGCCCAAAATTTCTCAATTTCCA	Genotyping *egr-1(ku285),* digest w/ Hpy188III
**oCG412 rev egr-1 ku285**	GACGTCTCCGCAGAAGCTTCGGTGGC	Genotyping *egr-1(ku285),* digest w/ Hpy188III
**oCG444 lim-6 3int sens**	GGATACGCTAACAACTTGGAAATGAAATAGGCGCCCTTCTTGAGA TTGCG	Cloning *lim-6 intron4* into pPD95.75
**oCG445 lim-6 3int rev**	CGACCTGCAGGCATGCAAGCTAAAGATTGACATATTGGAGACATC TGCC	Cloning *lim-6 intron4* into pPD95.75
**oCG461 sens sox-2 CRISPR**	GGTTGTCTTTTGCAGTGTCCGG	Genotyping *sox-2(syb737)*

**oCG462 rev sox-2 CRISPR**	CAGAGCCATTTTCTTCCGCTGTC	Genotyping *sox-2(syb737)*
**oCG463 sens sem-4 CRISPR**	GACGACGAATCTTCGATGTGGC	Genotyping *sem-4 (syb1287)*
**oCG464 rev sem-4 CRISPR**	GGGGGAAAGAGGGAAAATTAGCTG	Genotyping *sem-4 (syb1287)*
**oCG556 sens HMG**	GACAGCCCAGATCTGGGTACCCAAGGAGGTGGAAAAGCGAAGA	cloning of POP-1 HMG
**oCG557 rev HMG**	GACGGAGCTCGAATTCGGATCCTTAACTCTTATCCCTTCGTTTCTTCG	cloning of POP-1 HMG
**oCR017 ceh-6 fw**	GGCGGATGCAAGATTTTACG	Genotyping *ceh-6(gk665)*
**oCR018 ceh-6 rv wt**	GGATGACGACGAAGGTATGAG	Genotyping *ceh-6(gk665)*
**oCR019 ceh-6 rev gk665**	CTGTGACAATGTTCCCGGAG	Genotyping *ceh-6(gk665)*
**oCR029 fw ceh-18(mg57)**	CCCACACCAGTTTCCACAAATGGC	Genotyping *ceh-18(mg57)*
**oCR030 rv ceh-18(mg57)**	AGGCTAGAAAGTTCTACGGG	Genotyping *ceh-18(mg57)*
**oCR036 rv ceh-18 wt**	GCTCGCCGCCTCAATTCTTGAT	Genotyping *ceh-18(mg57)*
**oCR061 fw AfeI EpiDeg**	GAGGGTACCAGAGCTCAAGCGCTATTCACCTGGCACCGACTAC	Cloning AfeI and XhoI sites into pOD1988
**oCR062 rv XhoI EpiDeg**	CCAGACTCCACCAGTTGGACTTGATCCATCTCGAGTTATCTGGAACAAAATGTAAG	Cloning AfeI and XhoI sites into pOD1988
**oCR073 AscI nanob**	ATAAAAGGCGCGCCAAAAAATGGATCAAGTCCAACTGGT	Cloning *nanobodyGFP::zif-1* into pSJ671
**oCR074 U54 ApaI**	GTAATAGGGCCCTTAACCCTCACTAAAGGGAACAAAA	Cloning *nanobodyGFP::zif-1* into pSJ671
**oCR075 tm1422 fw**	GGGCCAGAAGATTGCACCAC	Genotyping *vang-1(tm1422)*
**oCR076 tm1422 rv wt**	GCATGCTGAAGCCGAAACGT	Genotyping *vang-1(tm1422)*
**oCR077 tm1422 rv**	CGCAATCGGTAGAATTGAAAATTTCGG	Genotyping *vang-1(tm1422)*
**oCR087 nr2073 fw**	GTAATGCGCGAAGCTTCCTG	Genotyping *lim-6(nr2073)*
**oCR088 nr2073rv wt**	GGGAGCCTATAGGTCAGCTCT	Genotyping *lim-6(nr2073)*
**oCR089 nr2073 rv**	CCTCCGCTTGGAAGGACAAAA	Genotyping *lim-6(nr2073)*
**oCR092 rh308 fw**	GTGATAATGCTCGTATTGTCTATTCCATTGATTCCTAT	Genotyping *fmi-1(rh308)*, digest w/ AseI
**oCR093 rh308 rv**	GTGGATGAGATCCGCCGTCAG	Genotyping *fmi-1(rh308)*, digest w/ AseI
**oCR094bis**	CCTCTTAAAAACTTACCTCTCAAATTTGAACTTATTCAAGC	Genotyping *egl-20(n585)*, digest w/ HindIII
**oCR095 n585 rv**	GAACATTGGCATTTGTGGGTTCAAAC	Genotyping *egl-20(n585)*, digest w/ HindIII
**oCR096 n1792 fw**	CTTCAAAACTGTGCGAATCGTTTGAGATTTCAGCCCT	Genotyping *lin-44(n1792)*, digest w/ AvrII
**oCR097 n1792 rv**	CCTTTTGACCCTACCCGCCGAAC	Genotyping *lin-44(n1792)*, digest w/ AvrII
**oCR105 zu310 fw**	CCCACATTCATCCATCGATCTTTCATAAT	Genotyping *par-1(zu310)*, digest w/ SspI
**oCR106 zu310 rv**	GTCTCTGCTGTTCAATATTTGCATTCG	Genotyping *par-1(zu310)*, digest w/ SspI
**oCR107 ok2126fw wt**	CTGAACTGCCTGCTGCCAGA	Genotyping *gpr-1(ok2126)*
**oCR108 ok2126rv**	CACGAAAGTCATCAACGTATGTAGTAAAG	Genotyping *gpr-1(ok2126)*
**oCR109 ok2126fw mu**	CCAAGGCTCGACGGTTTGC	Genotyping *gpr-1(ok2126)*
**oCR113 ga80 fw**	GCATAGTGAGTTCTGGAATTGCTCGAACTGTGTTATACTGCCC	Genotyping *bar-1(ga80)*, digest w/ BclI
**oCR114 ga80 rv**	CATCCATGGCCGACTATGAGCCGATCCCCACTCTTTCTGAT	Genotyping *bar-1(ga80)*, digest w/ BclI

**oCR122 q645 fw**	CGATGGATTTCGACCGGCACC	Genotyping *pop-1(q645),* digest w/ ClaI
**oCR123 q645 rv**	GATATAAAAATACACAAAAATGATGGCCGACGAAGAGCTCATCGA	Genotyping *pop-1(q645),* digest w/ ClaI
**oCR128 n1378 fw**	CAACACCGAATCCAAAAAACGAAAATCCACTGCTTGGCATG	Genotyping *sem-4(n1378),* digest w/ SphI
**oCR129 n1378 rv**	CCACGAGTTGTGAATGCGCGTCCAC	Genotyping *sem-4(n1378),* digest w/ SphI
**oCR138 syb971fw**	GACATTCGAAGCTCTGATGATG	Genotyping *lim-6(syb971)*
**oCR139 syb971rv wt**	GTGCAAAGATTAGAGCTCTGAC	Genotyping *lim-6(syb971)*
**oCR140 syb971rv mu**	GGGTATCTCGAGAAGCATTG	Genotyping *lim-6(syb971)*
**oCR144 n1051 fw**	CACTACAGAGTTATGGCAAACATCGACTACCTCTCGTTCCCAT	Genotyping *lin-18(n1051),* digest w/ NcoI
**oCR145 n1051 rv**	CCTGTCGCAATTTCACTTTCAACGGCTC	Genotyping *lin-18(n1051),* digest w/ NcoI
**oCR149 gm122 fw**	GACCACGATTTACTTCGGCAACG	Genotying *cam-1(gm122),* digest w/ BclI
**oCR150 gm122 rv**	CATCATATGTATAAAGTTTGCGAATCGGATTCTAATGAT	Genotying *cam-1(gm122),* digest w/ BclI
**oCR151 q544 fw**	CCTGTTGGCGGAGGAGGTTGATCATGTGG	Genotyping *sys-1(q544),*digest w/ AflIII
**oCR152 q544 rv**	GGCAAAAAGATCCTCACATGAAACACTGCGCAAATCACGT	Genotyping *sys-1(q544),*digest w/ AflIII
**oCR153 n671**	CCGCATTTTTCGTAGATCACACC	Sequencing *lin-17(n671)*
**oCR154 n671**	CGAGCACATTCCACAGAAGATG	Sequencing *lin-17(n671)*
**oCR155 lin-17p fw**	CTGAAGCTTACACTTTGTTCGCTC	Cloning *lin-17p* reporter
**oCR156 lin-17p rv**	CGGCTGCAGTTTGGAGAAGGAGCCAGTCTCTC	Cloning *lin-17p* reporter
**oCR157 wrm-1 fw**	GATGTTCTTCCGACTGAATGC	Sequencing and genotyping *wrm-1(ne1982ts)*
**oCR158 wrm-1 rv**	CTTGTGCTCCACCCATTTG	Sequencing and genotyping *wrm-1(ne1982ts)*
**pLG7F**	ACGCGTCGACGTGAAAACATAGTGTTTCCCAGTAC	*sox-2* antisens construct
**pLG7R**	GCTCTAGAGATATTATACATATTTCATAAAGCCAAC	*sox-2* antisens construct
**oSKS-233**	ATGAACTATACAAAGCTTCGAATTCTGCAGTCGACA TGTCTTCTGCTCGTCGTTGC	To amplify cki-1 for construction of pSJ1108
**oSKS-234**	ATTCATGCATAGGCCTGCGGCCGCGCTAGCCTAGTAT GGAGAGCATGAAGATCGAGTTC	To amplify cki-1 for construction of pSJ1108
**oSKS-235**	ATGGACTACAAGGACGACGATGACAAGTAAGGATCC GAATTCGAGCTCC	To insert Flag at the C-terminal of hmg-pop1 in pSJ769, for construction of pSJ1107
**oSKS-236**	ACTCTTATCCCTTCGTTTCTTCGTC	To insert Flag at the C-terminal of hmg-pop1 in pSJ769, for construction of pSJ1107
**oSKS-237**	AACCAAAGTTCTCACTGTCAGAACCA	To delete intron from cki-1 for construction of pSJ1112
**oSKS-238**	CTGTAGAACTCCGGAACACAATTCTCT	To delete intron from cki-1 for construction of pSJ1112
**psj6094sox-2 F**	TCGACATGATGATGGATCCGGATTCAGC	*6XHis::sox-2* construct
**psj6094sox-2 R**	GTGCGGCCGCAAGCTTGGTACC	*6XHis::sox-2* construct
